# Supplementary material for: A multisite randomized controlled trial of an early palliative care intervention in children with advanced cancer: The PediQUEST Response Study Protocol
Source: PLoS One. 2022 Nov 8;17(11):e0277212. doi: 10.1371/journal.pone.0277212 (PMC9642881; doi:10.1371/journal.pone.0277212)
Supplement: S1 Protocol — (PDF) [file pone.0277212.s002.pdf]

Administrative Information

Title

A multisite, parallel, randomized controlled trial to compare the effectiveness of an early palliative care intervention, the Pediatric Quality of Life and Evaluation of Symptoms Technology Response to Pediatric Oncology Symptom Experience (PediQUEST Response), versus Usual Cancer Care in children and adolescents with advanced cancer.

Short Title

The PediQUEST Response Intervention Study

Trial Registration

Clinicaltrials.gov: in process

Protocol Version and Date

Version 17.1   4/14/2022

Funding

R01 grant number 5 R01 NR016720-03 funded by the National Institutes of Health National Institute of Nursing Research

Roles and Responsibilities

Protocol contributors

Veronica Dussel, Liliana Orellana, Madeline Bilodeau, Chris Feudtner, Abby Rosenberg, Justin Baker, Jason Freedman, Ross Hays, Joanne Wolfe.

Veronica Dussel, Associate Research Scientist, Dana-Farber Boston Children's Blood Disorders and Cancer Center, co-investigator.

Liliana Orellana, Associate Professor of Biostatistics, Director Biostatistics Unit, Deakin University, Melbourne, Australia, study primary biostatistician.

Madeline Bilodeau, Research Project Manager, Division of Pediatric Palliative Care, Department of Psychosocial Oncology and Palliative Care, Dana-Farber Cancer Institute

Anne Reed-Weston, Research Assistant, Division of Pediatric Palliative Care, Department of Psychosocial Oncology and Palliative Care, Dana-Farber Cancer Institute

Rachel Holder, Research Assistant, Division of Pediatric Palliative Care, Department of Psychosocial Oncology and Palliative Care, Dana-Farber Cancer Institute

Christina Ullrich, Assistant Professor, Pediatrics, Harvard Medical School, Pediatric Hematology/Oncology, Pediatric Palliative Care, Boston Children's Hospital & Dana-Farber Cancer Institute, co-investigator.

Chris Feudtner, Director, Department of Medical Ethics; Attending Physician & Research Director, Pediatric Advanced Care Team & Integrated Care Service; The Children's Hospital of Philadelphia, co-investigator.

Jason Freedman, Inpatient Medical Director, Attending Physician, Division of Oncology, The Children's Hospital of Philadelphia, co-investigator.

Abby Rosenberg, Assistant Professor, Pediatrics; Division of Hematology/Oncology, University of Washington; Palliative Care and Resilience Program, Seattle Children's Research Institute, Medical Director, Adolescent and Young Adult Oncology, Seattle Children's Hospital Cancer and Blood Disorders Center, co-investigator.

Ross Hays, Medical Director, Pediatric Advanced Care Team, Seattle Children's Hospital Cancer and Blood Disorders Center, co-investigator.

Justin Baker, Chief, Division of Quality of Life and Palliative Care, Director, Hematology/Oncology Fellowship Program, Associate Member, Department of Oncology, St Jude Children's Research Hospital, co-investigator.

Marie Bakitas, PhD, Professor & Endowed Chair, University of Alabama School of Nursing; Associate Director, University of Alabama Center for Palliative and Supportive Care, co-investigator

Stefan Friedrichsdorf, MD, FAAP, Medical Director, Department of Pain Medicine, Palliative Care & Integrative Medicine, Children's Hospitals and Clinics of Minnesota

Cynthia Gerhardt, PhD, Director of Center for Biobehavioral Health, Nationwide Children's Hospital, co-investigator

Mary Cooley, PhD, Nurse Scientist, Phyllis F. Cantor Center for Research in Nursing and Patient Care Services, Dana-Farber Cancer Institute, co-investigator

Hasan Al-Sayegh, Biostatistician, Dana-Farber Boston Children's Blood Disorders and Cancer Center, Study Programmer

Joanne Wolfe, Division Chief, Pediatric Palliative Care, Department of Psychosocial Oncology and Palliative Care, Dana-Farber Cancer Institute, Principal investigator.

Authors' contributions

JW, VD, and LO conceived the study and wrote the protocol. VD, MBi, RHo, and ARW developed study materials. CU, CF, JF, AR, RHa, JB, MBa, SF, and CG contributed with aspects of study implementation. LO provided statistical expertise and will conduct the primary statistical analysis while HA-S will collaborate with statistical analysis. All authors contributed to refinement of the study protocol and approved this version.

Study sponsor information

Trial Sponsor: Joanne Wolfe, MD MPH

Address:

Dana-Farber Cancer Institute
Division of Pediatric Palliative Care
Department of Psychosocial Oncology and Palliative Care
DA2-012
450 Brookline Avenue
Boston, MA 02215
Telephone: 617-632-5286

Email: Joanne_Wolfe@dfci.harvard.edu

Role of study funder

Dr. Wolfe assumes overall responsibility for the design, conduct, and report of the trial.

The funding source had no role in the design of the study and will have no role during its execution, analyses and interpretation of the data, or decision to submit results.

Study administration structure

The proposed study will have a Steering Committee (SC) which will act as the study governing body. The sites' Institutional Review Boards (IRBs) will review and monitor research ethical issues. A Trial Coordinating Unit will be established at the Dana-Farber Division of Pediatric Palliative Care whereas Local Units will be established at each of the participating sites. The trial coordinating unit will also act as the Study Data Center (SDC). A Data and Safety Monitoring Board (DSMB) will be established.

	**Members – Role in Study**	**Roles and responsibilities**
**Principal Investigator and Research Associate**	Wolfe, Joanne<br>Dussel, Veronica	Design and conduct of PediQUEST Response<br>Preparation of protocol and revisions<br>Members of the Trial Coordinating Unit<br>Publication of study reports
**Steering Committee**	Wolfe, Joanne (PI)<br>Dussel, Veronica<br>Ullrich, Christina (PI-DFCI),<br>Orellana, Liliana<br>Baker, Justin (Co-investigator-St Jude's)<br>Feudtner, Chris (PI-CHOP)<br>Freeman, Jason (Co-PI-CHOP)<br>Rosenberg, Abby (PI-Seattle Children's Hospital(SCH))<br>Hays, Ross (Co-PI-SCH)	Agreement of final protocol<br>Participate in meetings every other month to review trial progress and data quality and if necessary proposing changes to the protocol and/or investigator brochure to facilitate the smooth running of the study.

	Waldman, Elisha (PI-LCHC)<br>Tammy Kang (PI – TCH)	
**Trial Coordinating Unit**	Wolfe, Joanne<br>Dussel, Veronica<br>Orellana, Liliana<br>Project Manager<br>Hasan Al-Sayegh	Study planning<br>Generation of study materials (for participants, training, and PediQUEST web)<br>Development and maintenance of PediQUEST web<br>Responsible for trial master file<br>Assist sites with ethics committee continuing reviews and protocol amendments<br>A*dverse events* and protocol violation reporting to IRBs<br>Budget administration and contractual issues with individual centers<br>Organization of SC, SDC, DSMB, and biweekly study meetings<br>Acts as the <u>Study Data Coordinating Center</u><br>• Develop study SOPs<br>• Provide data management plan<br>• Audit data quality and trial progress reports and provide feedback and advice to local PIs<br>• Send reports to DSMB and SC<br>• Data verification<br>• Transcription of audio files<br>• Maintenance of trial IT system and data entry
Local Research Units	Local PIs (see members above)<br>Site Research Coordinators (RCs)	<u>*Local PIs*</u> will oversee the site's investigation activities and liaise with PI<br>Participate in SC and SC's quarterly meetings<br>Assist with coordination of training at the sites<br>Participate in study promotion activities<br>Participate in listserv activities<br>Report any adverse event or protocol violation to the SDC and PI<br><u>*RCs*</u> will be responsible for identification, recruitment, data collection and completion of CRFs, along with follow up of study patients and adherence to study protocol and investigator SOPs

		Compile and report to PI any adverse event or protocol violation<br>Prepare and follow-up IRB documentation
Expert Palliative Care (PC) ListServ	Friedrichsdorf, Stefan<br>Nurse Practitioner expert<br>Social work expert<br>Chaplain expert<br>PI<br>Local PIs	Design and review of training materials<br>Participate in PC teams Training<br>Participate in PediQUEST Response Listserv discussions<br>Participate in meetings with PI and Trial Coordinating Unit members to provide feedback about the use of listserv
Data and Safety Monitoring Board	Bakitas, Marie<br>Gerhardt, Cynthia<br>Cooley, Mary<br>Orellana, Liliana<br>Dussel, Veronica	Review data quality and trial progress reports.<br>Participate in quarterly meetings and report and make recommendations to the SC regarding:<br>• Data quality and completeness<br>• Patient accrual and retention<br>• Adverse events<br>• Protocol violations or deviations<br>• Review of interim results and recommendations

WHO Summary

DATA CATEGORY	INFORMATION
Primary registry and trial identifying number	ClinicalTrials.gov (NCT03408314)
Date of registration in primary registry	1/24/18
Secondary identifying numbers	1R01NR016720-01 (NINR-NIH Grant Number)
Source(s) of monetary or material support	NINR, NIH
Primary sponsor	*Joanne Wolfe*, MD, MPH<br>Division of Pediatric Palliative Care<br>Dept of Psychosocial Oncology and Palliative Care<br>Dana-Farber Cancer Institute<br>DA2-012<br>450 Brookline Avenue<br>Boston, MA 02215<br>Telephone: 617-632-5286

	joanne_wolfe@dfci.harvard.edu
Contact for public queries	*Joanne Wolfe*, MD, MPH (see contact details above)
Contact for scientific queries	*Joanne Wolfe*, MD, MPH (see contact details above)
Public title (*aka* Short Title)	The PediQUEST Response Intervention Study
Scientific title	*A multisite, parallel, randomized controlled trial to compare the effectiveness of an early palliative care intervention, the Pediatric Quality of Life and Evaluation of Symptoms Technology Response to Pediatric Oncology Symptom Experience (PediQUEST Response), versus Usual Cancer Care in children and adolescents with advanced cancer.*
Countries of recruitment	U.S.
Health condition(s) or problem(s) studied	Pediatric advanced cancer
Intervention(s)	*Active comparator: PediQUEST Response consists* of feedback of electronic patient reported outcomes (child symptoms and quality of life) to providers and families on a weekly basis coupled with involvement of the palliative care team, who should meet with families and providers ideally within three weeks of randomization and follow-up at least monthly as deemed necessary based on PediQUEST feedback reports and other clinical indications.<br>*Control arm:* usual cancer care
Key inclusion and exclusion criteria	Ages eligible for study: ≥2 years old children, adolescent and young adults<br>Sexes eligible for study: both<br>Accepts healthy volunteers: no<br>Inclusion criteria: pediatric oncology patients (and one of their parents) within the above mentioned age range, receiving routine cancer care at one of the participating centers –and are not in remission and off cancer-directed treatment—that have (i) advanced cancer defined as: at least a 2-week history of progressive, recurrent, or non-responsive cancer of any type, or any brainstem tumor, or a grade IV Glioblastoma Multiforme, or decision not to pursue cancer-directed therapy in place, or any other progressive/recurrent solid or brain tumor, and are (ii) palliative care naïve defined as the palliative care team not currently integrated into their regular cancer care.<br>Exclusion criteria: The patient-parent dyad would be excluded if patients are older than 18 years of age and no parent is involved in his/her care, or if patient is under the care of foster parents who do not have legal guardianship, or if both parents do not

	speak English or Spanish, or are unable to understand and complete surveys, or if the patient has a non-brainstem low-grade glioma with localized progression/relapse only, or is expected to receive a stem cell transplant within the next 18 weeks or life expectancy is less than two months.
Study type	Interventional<br>Allocation: randomized<br>Assignment: parallel<br>Masking: not feasible<br>Primary purpose: quality of life improvement
Date of first enrollment	April 2018
Target sample size	136 (target enrollment: 200)
Recruitment status	Actively Recruiting
Primary outcome(s)	*Child Quality of Life:* difference between intervention and control arms of the mean Pediatric Quality of Life Inventory 4.0 (PedsQL) total scores over 16 weeks, as reported by (a) the parent for all enrolled children and (b) the patient (if 5 years of age or older).
Key secondary outcomes	*Child Quality of Life:* difference between intervention and control arms of the mean Pediatric Quality of Life Inventory 4.0 (PedsQL) subscale scores (physical and psychosocial) over 16 weeks, as reported by (a) the parent for all enrolled children and (b) the patient (if 5 years of age or older).<br><br>*Child Symptom Burden:* difference between intervention and control arms of the mean PediQUEST-Memorial Symptom Assessment Scale (PQ-MSAS) total and subscale scores over 16 weeks, as reported by (a) the parent for all enrolled children and (b) the patient, if 13 years of age or older.<br><br>*Parent distress (anxiety, depression)*: difference between trial arms of the mean Spielberger's-State Anxiety Inventory, Center for Epidemiologic Studies Short Depression Scale scores over 16 weeks (measured every four weeks), and symptom related stress (measured at study entry and 16 weeks).<br><br>*Family activation:* change in BRIEF-Cope active coping, planning, and instrumental support scale scores between study entry and 16 weeks, and "symptom treatment activation" over 16-weeks (measured by the use of non-pharmacologic strategies for symptom treatment: total No. of complementary therapies (CT) and No. of different CT reported by parents, and No. of

	documented psychosocial clinician encounters) between intervention and control arms.

Table of Contents

Administrative Information 1
Title 1
Short Title 1
Trial Registration 1
Protocol Version and Date 1
Funding 1
Roles and Responsibilities 1
WHO Summary 5
Table of Contents 9
List of Abbreviations 11
Protocol Summary 12
1. Introduction 14
1.1. Background and Rationale 14
1.2. Study Objectives 18
1.3. Trial Design 19
2. Methods: Participants, interventions, and outcomes 21
2.1. Study Setting 21
2.2. Eligibility Criteria 22
2.3. Interventions 24
2.4. Outcomes 29
2.5. Participant timeline 31
2.6. Sample Size 35
2.7. Recruitment 36
3. Methods: Assignment of interventions 38
3.1. Allocation (sequence generation, concealment, and implementation) 38
3.2. Blinding 39
4. Methods: Data collection, management, and analysis 39
4.1. Data collection methods 39
4.2. Data management 39
4.3. Statistical methods 40
5. Methods: Monitoring 42
5.1. Data Monitoring 42
5.2. Potential Risks and Benefits 44
5.3. Auditing 46
6. Ethics and Dissemination 46
6.1. Research Ethics/IRB Approval 46
6.2. Consent and Assent 47
6.3. Confidentiality 48
6.4. Declaration of Interests 49
6.5. Access to Data 49
6.6. Ancillary and post-trial care 50
6.7. Dissemination policy 50

6.8. Anticipated Problems and Solutions ____ 50
7. Study Timeline ____ 51
8. Importance of the Knowledge to be Gained ____ 51
9. References ____ 51

List of Abbreviations

PediQUEST/PQ: Pediatric Quality of Life and Evaluation of Symptoms Technology
PQ Response: PediQUEST Response to Pediatric Oncology Symptom Experience (intervention)
HRQoL: Health-related Quality of Life
PC: Palliative care
RCT: Randomized Controlled trial
SC: Steering Committee
DSMB: Data and Safety Monitoring Board
SDC: Study Data Center
LDC: Local data centers
PI: Principal Investigator
Co-I: Principal Investigator
PM: Project Manager
RC: Research Coordinator
CRF: Case Report Forms
GCPs: Good Clinical Practices
SOP: Standard Operating Procedures
DFCI: Dana-Farber Cancer Institute
DFBCC: Dana-Farber/Boston Children's Cancer and Blood Disorders Center
CHOP: Children's Hospital of Philadelphia Cancer Center
SCH: Seattle Children's Hospital
LCHC: Ann & Robert H. Lurie Children's Hospital of Chicago
TCH: Texas Children's Hospital
P.O.T.: Primary Oncology Team
e-PROMs: Electronic-Patient Reported Outcomes
IQR: Interquartile range
PedsQL: Pediatric Quality of Life Inventory 4.0
PQ-MSAS: PediQUEST-Memorial Symptom Assessment Scale
S-TAI-State and S-TAI-Trait: Spielberger's-State-Trait Anxiety Inventory State and Trait components
CES-D-10: Center for Epidemiologic Studies Short Depression Scale
CT: Complementary Therapies
16-w: 16-weeks
AYA: Adolescents and Young Adults
EPEC-Pediatrics: Education in Palliative and End-of-life Care for Pediatrics curriculum
MCID: Minimal Clinically Important Difference
MR: Missing at random
PHI: Personal Health Information
IRB: Institutional Review Board

Protocol Summary

Background: Integration of palliative care (PC) has been associated with better health related quality of life (HRQoL) and longer survival in adults and their caregivers. Yet, only a few randomized controlled trials (RCTs) have evaluated whether PC integration improves child and family outcomes. We propose to evaluate the effects of an early PC intervention (PediQUEST Response) for pediatric oncology patients (from here on called *patients*) with advanced cancer on patient and parent outcomes.

Aims: Aim 1 To evaluate whether PediQUEST Response, compared to usual care, improves patient's HRQoL (primary outcome) and symptom burden. Aim 2 To evaluate the impact of the intervention on parent psychological distress and symptom-related stress outcomes. Aim 3 To compare family activation between study entry and 16 weeks, use of non-pharmacologic strategies for symptom treatment, and No. of documented psychosocial clinician encounters between intervention and control arms.

Design: *Multisite, randomized (1:1), controlled, un-blinded, effectiveness trial* comparing: PediQUEST Response (intervention) vs usual cancer care (comparator).

Setting: Dana-Farber/Boston Children's Cancer and Blood Disorders Center (DFBCC), Seattle Children's Hospital, Children's Hospital of Philadelphia Cancer Center, Ann and Robert H. Lurie Children's Hospital of Chicago (LCHC), and Texas Children's Hospital (TCH).

Participants: The target sample size (SS) is 136 patient-parent dyads (N=68/arm) ≥2 years old receiving cancer care at the participating sites and with advanced cancer or any other progressive/recurrent solid or brain tumor who do not have the palliative care team currently integrated into their regular oncology care. To achieve this SS, 200 dyads will be recruited.

Interventions: *PediQUEST Response*: consists of combining a patient-mediated activation intervention (weekly feedback of patient-reported outcomes to families and providers using the online PediQUEST web system) with early integration of the PC team (consisting of an initial meeting with providers and family with subsequent follow-up including a monthly encounter triggered by PediQUEST reports and other clinical indications). PC teams will receive standardized training before starting the intervention and ongoing support through an expert listserv. *Usual Cancer Care (comparator)*: participants in this arm will receive usual cancer care provided at the sites, which can include PC consultation as deemed necessary by oncologist, and will use the PediQUEST web system to complete surveys but no reports will be generated.

Methods: Following enrollment, patient (if older than 5 years) and parents of all enrolled patients will receive weekly PediQUEST-Surveys (patient HRQoL and symptoms). Parents will complete a Baseline Survey Packet. A two-week run-in period will identify and exclude non-responder dyads (i.e. <2 answered PediQUEST-Surveys out of 3 assigned). Responders will be randomized to the intervention or control arms (concealed allocation) and followed up for 16-weeks (16-w). Those assigned to the intervention will begin receiving PediQUEST Response (feedback reports + response team intervention). Parents in both arms will be assigned monthly questionnaires (parental distress, HRQoL, burden, and use of complementary therapies). All surveys will be administered through PediQUEST web. Process measures will be collected throughout the study, and semi-

structured exit interviews conducted in participating patients, parents, and a sub-sample of providers. Participants in the intervention arm will be offered continued care by the PC team once the intervention ceases.

Outcomes: Average difference between the two treatment arms in: *(i) Patient outcomes:* mean patient total HRQoL scores over 16-w, as measured weekly by the Pediatric Quality of Life Inventory 4.0 (PedsQL) reported by a) the parent for all enrolled patients and, b) the patient if older than 5 years (primary study outcomes); we will also look at mean PedsQL subscale scores over 16-w for parent and patient; mean patient symptom burden scores, measured weekly through the PediQUEST-Memorial Symptom Assessment Scale (PQ-MSAS) reported by a) the parent for all enrolled patients, and b) the patient if 13 years old or older; *(ii) Parent distress*: mean anxiety and depression scores over 16-w measured every 4 weeks through Spielberger's-State Anxiety Inventory and the Center for Epidemiologic Studies Short Depression Scale, and change in symptom-related stress score between study entry and 16-w measured with an adapted version of the stress-portion of the Response to Stress Questionnaire-Pain; *(iii) Family activation*: Change in family activation between study entry and 16-w, measured by three Brief-COPE scales, and "symptom treatment activation" (measured by the use of non-pharmacologic strategies for symptom treatment: total No. and No. of different complementary therapies reported by parents, and No. of documented psychosocial clinician encounters (from clinical records) over 16-w).

1. Introduction

Protocol is written following the Standard Protocol Items: Recommendations for Intervention Trials Statement (SPIRIT 2013).[1]

1.1. Background and Rationale

Integration of palliative care (PC) into healthcare through early consultation, education, or symptom monitoring and feedback, has been associated with better health related quality of life (HRQoL) and longer survival in adults[2–4] and their caregivers.[5] A growing number of RCTs in this regard led to the American Society for Clinical Oncology's endorsement of early PC integration for adult patients with "*metastatic cancer and/or high symptom burden*."[6] International and national organizations also have called for early PC integration for children,[7–9] however, very few randomized controlled trials (RCTs)[10,11] have evaluated whether PC improves child and family outcomes. Evidence mostly comes from non-experimental studies.[12–17] Children and teens with advanced cancer endure a high degree of suffering,[18,19] linked to impaired patient[20,21] and family[22,23] survivorship.[13,18,19,24,25] Yet, PC integration is highly variable in terms of availability,[26,27] frequency and timing of PC referrals,[28,29] and limited by primary oncologist and family beliefs.[30] We propose to evaluate the effectiveness of an early PC intervention for pediatric patients with advanced cancer, called the Pediatric Quality of Life and Evaluation of Symptoms Technology Response to Pediatric Oncology Symptom Experience (PediQUEST/PQ Response) intervention.

PediQUEST Response seeks to ultimately improve child symptom burden and consequently their health related quality of life (HRQoL), as well as parental distress and burden. The main proposed mechanism of the intervention is to *activate* both providers and parents through the use of an electronic patient reported outcomes system (PediQUEST web) combined with integration of a PC consulting team (Response team) into cancer care. Using a multisite parallel randomized controlled trial, we will compare PediQUEST Response vs. usual care at five large U.S. pediatric oncology centers.

1.1.1. Preliminary Data.

Under the leadership of Dr. Wolfe, this work began several years ago with bereaved parents' assessments of the quality of end-of-life care for children with cancer.[31–33] Collectively, these studies identified substantial child suffering from cancer-directed therapies and symptoms.[18,34–36]

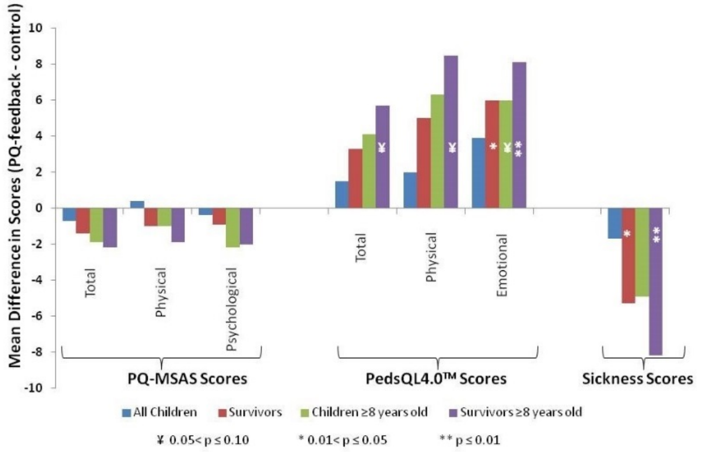

Figure 1. PediQUEST Feedback intervention results (from Wolfe et al., JCO 2014).[23] The figure shows estimated average score differences between intervention and control groups for all children (blue bar), and exploratory subgroup analysis (red, green, and purple bars). *Abbreviations*: PQ-MSAS, PediQUEST-Memorial Symptom Assessment Scale; PedsQL4.0™, Pediatric Quality of Life Inventory4.0™.

1.1.1.1. The PediQUEST RCT Pilot Study[37,38] (Clinicaltrials.Gov: Nct01838564)

This pilot RCT study (1K07 CA096746-01, PI: Wolfe, Dana-Farber Cancer Institute (DFCI) Protocol #:04-321) was carried out at 3 large pediatric oncology centers. The primary aim was to evaluate whether providing electronic-Patient Reported Outcomes (e-PROMs) feedback (symptom and HRQoL summary scores) to families and clinicians of children with advanced cancer, improved child's HRQoL and symptom burden. In both arms, e-PROMs were collected through the initial version of the PediQUEST system, which was completed on a tablet. When symptom or HRQoL scores met pre-defined thresholds that indicated distress, an email was sent to primary providers. Over a 20-week follow-up, 699 surveys were completed with high self-report rates (88% for younger children and 98% for teenagers). As shown in Figure 1, in the intervention arm, *all* scores changed in the expected directions but changes did not reach statistical significance. In post-hoc subgroup analyses looking at older children and those who survived 20 weeks, larger improvements were observed in the intervention group, especially in emotional HRQoL scores. Importantly, parents reported that feedback helped them to understand how their child was feeling (75%). Providers reported that feedback was useful to speak with patients (50%), provided new information about psychosocial (61%) but not as much about physical issues (22%), and contributed to initiating a psychosocial consult at least sometimes (56%). *Taken together these results support the feasibility of collecting e-PROMs in these children, while highlighting the need for a stronger intervention.*

1.1.1.2. The PQ Cohort Study[19,39]

We also used data from the PQ cohort to describe the child's experience.[19,39] Over a 9-month follow-up, 920 surveys were answered, a median of 8 surveys per patient. On average, children reported 3 distressing symptoms (interquartile range (IQR): 1-6) per administration. The four most frequent symptoms were pain (48%), fatigue (46%), drowsiness (39%), and irritability (37%). In multivariable models, receiving high or moderate intensity cancer-directed treatment, disease progression and female sex were associated with an increase in symptom burden. HRQoL was also impaired. The presence of distressing symptoms was significantly associated with lower HRQoL scores. This association persisted after controlling for disease related variables. *The data suggests that symptoms could be intermediary variables by which illness and treatments affect HRQoL, and provide foundation for our intervention hypothesis.*

1.1.2. PediQUEST Response Intervention Development

The intervention was developed and piloted following the Medical Research Council framework.[40] In addition to an extensive review of the literature, we conducted a 2-phase formative research study that informed the design, testing, and revision of the proposed PQ Response intervention.

1.1.2.1. Conceptual Model for the PediQUEST Response Intervention: Wilson and Cleary HRQoL model

We chose the HRQoL model by Wilson and Cleary[41] (later revised by Ferrans)[42] as our conceptual framework. This causal model seeks to explain the different factors that influence HRQoL. It distinguishes five measurement levels, i.e. biological, symptoms, functioning, general health perception, and overall HRQoL. The model shows that both individual characteristics and environmental factors, have significant influence on HRQoL. Our own data, which as explained in 1.1.1.2, suggests that symptoms play an intermediary role in the relationship between biological/physical factors and HRQoL,[39] provides support to the model. Based on these data and the existing literature, we developed an adapted version of the model (Figure 2) specifying what we identified as the "main environmental" influences on the HRQOL of a child with cancer: parents and the health care system.

1.1.2.2. Formative Work Phase I: Understanding symptom management processes

During the first phase, we explored facilitators and barriers through two focus groups, seven in-depth stakeholder interviews, and an expert panel. We subsequently conducted a multiple comparative case study to further understand symptom management processes. To this end we administered weekly web-based PQ surveys with no feedback and when symptom distress was reported we interviewed parents, children, and providers. Of the 33 patients enrolled and registered into the PediQUEST system, 27 provided data for this phase. Two hundred and forty-four symptom distress reports were evaluated. Despite the persistence of distressing symptoms over time, findings highlighted that it is difficult for providers and parents to prioritize quality of life concerns, mainly because physical and emotional symptoms are interpreted as an unavoidable consequence of treatment (and illness). This process of "normalization" seems to impact on symptom management strategies. We identified that pharmacologic treatments predominate over non-pharmacologic interventions and that, no PC consultations were initiated by primary oncologists at this "early" illness stage. Further, during the pilot of the intervention we also identified that this "normalization" could impact on the PC team ability to respond to symptom distress.

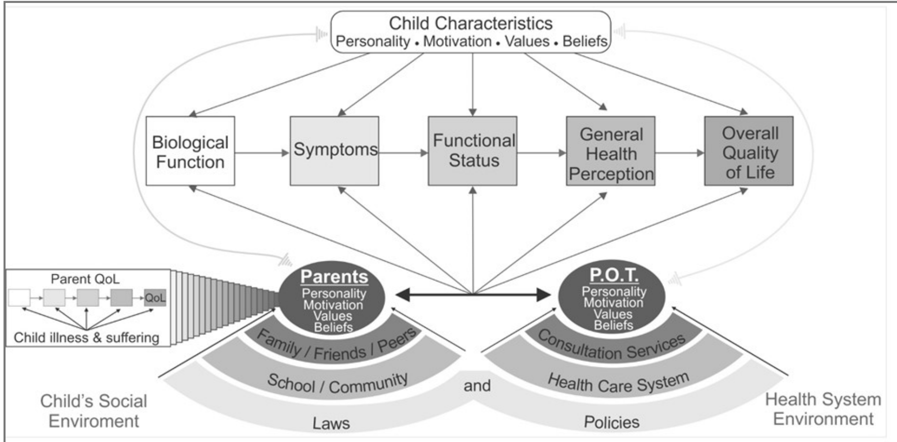

Figure 2. Wilson and Cleary's HRQoL model adapted to the context of pediatric cancer. The model explicates the influence of the own child characteristics and environment on child's HRQoL. Child's parents and primary oncology team (P.O.T.) are shown as the two "main" environmental influences on child's HRQoL. In addition, in the box on the left we illustrate how child's illness and suffering, in turn, play an influential role on parental HRQoL.

1.1.2.3. Rationale for the PediQUEST Response Intervention: Activating the symptom management process

Based on these results, we proposed that PQ Response should "activate," i.e. increase efficacy of symptom management processes through two main pathways (Figure 3): 1) e-PROMs which trigger a patient-mediated activation of family and team ("A" arrows). 2) Early integration of the Response team, which would activate the family-oncologist dyad by providing guidance about comprehensive symptom management ("B" arrow). Thus we propose that the intervention will result in improved child's HRQOL scores (primary outcome) and child symptom burden (aim 1), improved parent's anxiety, depression, burden, and HRQOL (aim 2), ("C" arrows). In addition, we will measure family activation, and other process measures, as intermediary outcomes (aim 3). Use of e-PROMs as an instrument for provider activation and promotion of patient-centered care is innovative in pediatric serious illness. e-PROMs allow for the report of the status of a patient's health condition directly from the patient, and enable systematic data collection and feedback.[43] The proposed RCT will be the first to evaluate the combination of family activation through e-PROMs feedback with response from PC teams in children with advanced cancer.

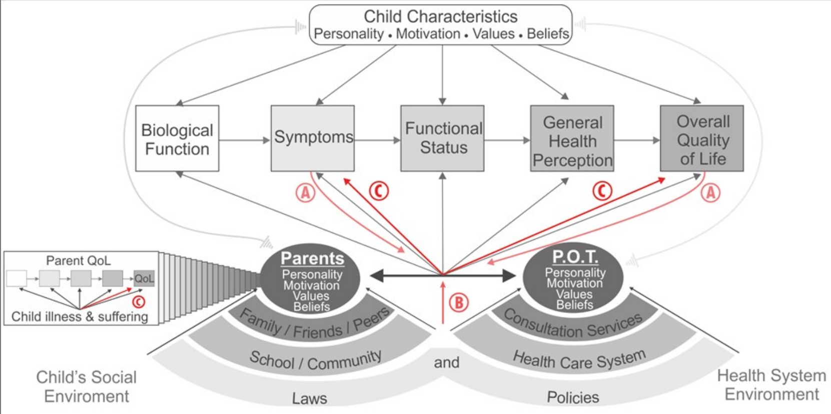

Figure 3. PediQUEST Response intervention and the HRQoL model. PediQUEST Response will interact with the model to ultimately improve child and parent outcomes (red arrows):

- (A) Patient-mediated activation through the PediQUEST web system
- (B) Response team early integration into cancer care
- (C) Expected effects of PediQUEST Response intervention

Patient activation is defined as the extension of self-efficacy into self-management. We chose to target activation because this has been shown to be a changeable characteristic that is associated with improved self-management behaviors[44] and improved psychological wellbeing and HRQoL among adult patients.[45,46] In pediatrics, higher parenting self-efficacy in several different pediatric populations has been associated with better adherence to medications and improved clinical outcomes.[47–50]

1.1.2.4. Formative Work Phase II: Piloting PediQUEST Response

During the second phase of formative research, we piloted the intervention using the same multiple comparative case study design and introduced each component step-by-step. First, to the weekly PQ surveys we added the feedback reports which were sent to parents, providers and patients if older than 8 years old (because younger children do not have their own PQ web account). Next, we introduced the Response team intervention which initially included PQ surveys + feedback+ contact with a PC pediatric nurse practitioner. We uncovered that having only one person delivering the intervention limited the feasibility of the intervention, and therefore, transitioned to a PQ Response team consisting of a PC physician, nurse practitioner and social worker. Of the 33 patients enrolled and registered, 23 received at least one intervention component, and 21 were exposed to the full intervention. During the pilot we substantially modified the way in which the intervention was delivered until we identified strategies that allowed for effective integration into care. Final components of the intervention are presented in section 2.3.1. Patients exposed to the final version of PQ Response showed a promising improvement in symptom distress, and parents and providers reported the

intervention as acceptable. *Taken together, these data provide preliminary evidence about the feasibility and acceptability of the PediQUEST Response intervention.*

1.1.3. Rationale for choice of comparator group

The goal of this study is to evaluate whether PediQUEST Response can improve current practice. For this reason, we chose a "usual cancer care" control group, which we hope will represent the care that patients and families receive on a day-to-day basis. We are aware that this choice involves some potential for bias, as patients and providers may become influenced as the trial develops. However, we believe the risk for bias is low (see section 1.3.1, Design Rationale) and we will also take some actions to minimize the risk.

Summary Our research began by identifying the need for enhanced pediatric PC. Based on these findings, we undertook a systematic approach to intervention development, providing strong foundations for the proposed research plan. Building on our experience of having successfully completed the *first* supportive care RCT among children with advanced cancer, our interdisciplinary research team is leading the way in intervention research to address the PC needs of children with advanced cancer through development of the PQ Response Intervention. If successful, this will be one of the first rigorously tested PC interventions in pediatric advanced cancer, and could have significant clinical and policy implications for these children and families, and the tens of thousands of children living with other serious illnesses.

1.2. Study Objectives

The overall goal of the study is to conduct a **multisite randomized controlled trial** to evaluate whether the **PediQUEST Response intervention improves pediatric oncology patient and parent outcomes** compared to usual care.

Specific study goals include to evaluate the effects of the PediQUEST Response intervention on:

1.2.1. Aim 1: Patient Outcomes

The primary goal of this study is to compare the effect of the intervention vs. usual care on patient's HRQoL, measured through the Pediatric Quality of Life Inventory 4.0 (PedsQL) reported by a) the parent for all enrolled patients and, b) the patient if ≥5 years of age.[51,52] A secondary patient outcome is symptom burden, measured through the PediQUEST-Memorial Symptom Assessment Scale (PQ-MSAS) reported by a) the parent for all enrolled patients, and b) the patient if ≥13 years old.[53,54] All these outcomes will be measured weekly for 16 weeks.

H1: Compared to controls, patients receiving the intervention will have higher PedsQL and lower PQ-MSAS total scores averaged across the 16-week study period.

1.2.2. Aim 2: Parent distress

Secondary goals of the study include evaluating the impact of the intervention on average parent psychological distress, as measured by the Spielberger's-State-Trait Anxiety Inventory (S-TAI)-State component[55] and the Center for Epidemiologic Studies Short Depression Scale (CES-D-10) Scale,[56] measured every 4 weeks for 16

weeks, and symptom-related stress as measured by an adapted version of the stress portion of the Response to Stress Questionnaires-pain[57] (*a*RSQ-pain) measured at study entry and week 16.

H2: Compared to parents of patients in the control group, parents of those receiving the intervention will report better state-anxiety, depression, and symptom-related stress scores.

1.2.3. Aim 3: Family activation

Other secondary study goals focus on assessing the effect of the intervention on family activation. Activation will be measured through an adapted version of the Brief-COPE[58] (at study entry and 16 weeks), and by specifically evaluating "symptom treatment activation," measured by the use of non-pharmacologic strategies for symptom treatment: total No., and No. of different, complementary therapies reported by parents, and No. of documented psychosocial clinician encounters, reported every four weeks.

H3: Families in the intervention group will demonstrate higher levels of active coping, planning, and instrumental support coping styles as well as higher levels of symptom treatment activation.

1.3. Trial Design

We will conduct a *randomized, controlled, un-blinded, multisite, effectiveness trial* with two parallel groups, PQ Response (intervention) vs. usual cancer care (comparator or control). Primary outcomes will be the mean over 16-weeks of the child HRQoL total score reported by (a) the parent and (b) the patient if ≥5 years of age. Randomization will be stratified by site, age, and type of cancer with a 1:1 allocation. We intend to enroll a total of 200 expecting to achieve our target sample size of 136 pediatric oncology patients ≥2 years old with advanced cancer (N=68/arm) from five participating sites over a 36-month period (start times staggered by site, see more in section 2.6 Sample size). The expected start for enrollment is September 2017.

Families (patient and one of the parents) will be enrolled for a total of 18 weeks. After an initial 2-week run-in period, families who answered a total of at least two PediQUEST surveys per participant during the run-in period (responders), will be randomized to the intervention or usual care (Figure 4).

1.3.1. Design Rationale

Because we are testing a model of care, the study is designed with a strong pragmatic emphasis.[59] We use existing PC clinicians to deliver the intervention, do not require extra visits, and have chosen outcomes that are highly relevant to participants. We expect that this pragmatic design will contribute to understanding how to integrate pediatric PC services in real life settings.

We opted for individual randomization as the best strategy, even though there is risk for contamination. Potential sources of contamination include: oncology clinicians having patients in both arms and subsequently becoming "activated," PC response teams becoming better "activators" and thus changing the way they intervene with "usual care" group referrals, or, patients and families "activating" other families. Based on our prior RCT and formative work, we estimate these risks are small. At any given time, most oncology providers will have 1-2 patients on study and these providers are not specifically targeted in this intervention (they will be informed about study procedures but will not receive specific training); neither oncology providers nor PC Response team will receive PediQUEST reports of patients in the "usual cancer care" group, and, patient-to-patient activation was not an issue during the pilot. For these reasons we believe that the possibility of contamination is limited. Further, alternatives are not practicable. Oncologist-level randomization results in the same risks because patients are usually seen by more than one oncologist (and given our outcomes are at the patient level we preferred this alternative). A cluster trial may be the ideal design, but it would necessarily involve a large number of sites significantly increasing the cost/benefit ratio.

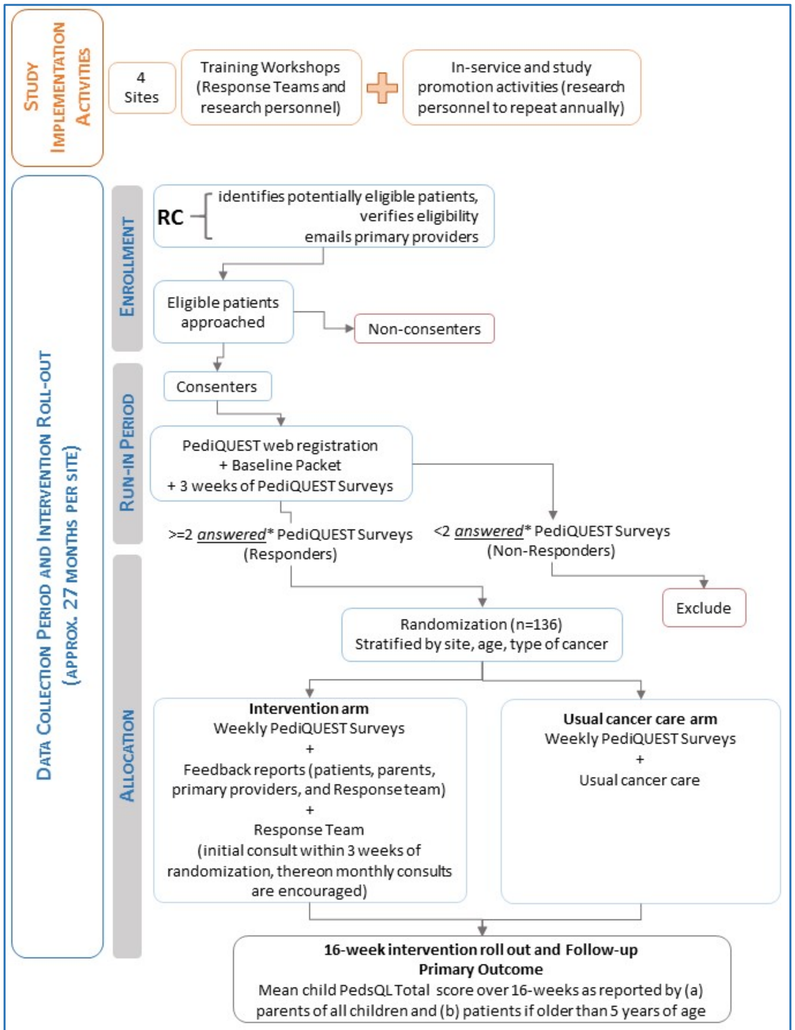

Figure 4. The PediQUEST Response Study Flowchart. * PQ-Surveys are considered *answered* if ≥1 in the dyad (patient or parent) submits a complete or incomplete survey

An additional limitation of the study is the relatively small sample size, mostly determined by the small size of the target population, which despite randomization, carries the risk for imbalance of prognostic factors between arms. To minimize this risk, we will perform a stratified randomization.

The 2-week run-in period is established to identify and exclude non-responder dyads before randomization to decrease the frequency of missing data for the primary outcome. Non-responders are defined as those who of the three PQ-Surveys assigned during the run-in period have <2 *answered*. PediQUEST-Surveys will be considered *answered* if the survey could be scored, i.e. a survey with >50% of the questions answered. Both patient and parent must be responders for the dyad to continue. Pilot data showed that participants who did not register in the PediQUEST web system did not differ from those in the responder group in terms of diagnosis, age, or survival. The 16-week follow-up period was defined for practical reasons. From our prior work we know that (a) short enrollment periods, enhance recruitment and retention; and (b) intermittent attrition increases

over time, especially after 20 weeks. Considering the intra-subject variability of HRQoL scores over time, we chose a 16-week period to allow for a more precise measure of the main outcome while aiming to improve enrollment and intermittent attrition. In addition, pilot data showed the need to allow enough time for intervention effects to be demonstrated (for example a preventive measure only takes effect when the next treatment cycle begins) making shorter follow-up less advisable. Finally, the 16-week period also poses an acceptable burden on PC teams whose workload will be increased without a corresponding increase in staff.

1.3.2. Rationale for using parent and patient reports to measure patient outcomes

The primary study goal is to evaluate the effects of PQ Response on patient HRQoL and symptom burden across a wide age range of children, adolescents, and young adults (AYA). While self-report remains the gold-standard for these type of outcomes, there is an increasing body of evidence suggesting that in the case of children/AYA there is considerable value in having both parent and child/AYA reports.[60] Each informant in the parent-patient dyad provides a unique perspective, influenced by their experience, personal characteristics, role, and environmental aspects, among others.[61] Together, these reports provide a broader picture of the child/AYA's situation.[62] Yet, one of the consequences of using "multiple informants,"[63] is that the information may be non-concordant (divergent).[64,65] The optimal approach to consolidate multiple informant responses into a unique child/AYA outcome is still unresolved.[61] Acknowledging the clinical importance of considering both "voices" (i.e. patient and parent), we will measure patient outcomes (HRQoL and symptom burden) using both parent and patient reports. The chosen instruments allow parental report across the study's full age range, and patient self-report from the age of five for HRQoL, and from the age of 7 for symptom burden (however, because the younger child version of the symptom instrument for ages 7-12 does not measure the full range of symptoms we will not use it as an outcome measure, see section 2.4.2.1 for details). Given the lack of consensus as to how to handle multiple informant information, the effects of the intervention on patient outcomes will be analyzed separately for each of these informants. Of note, the data collected in this trial will also provide the opportunity to explore convergence and divergence of child/AYA and parent reports.

2. Methods: Participants, interventions, and outcomes

2.1. Study Setting

Study centers invited to participate must have an established PC interdisciplinary program with Hospice and Palliative Medicine Board certified physician specialists, treat over newly 200 diagnosed cancer patients per year, and do not already have an early PC integration program. The rationale for these requirements is that the intervention requires a specialized PC team and sufficient patient volume is needed to ensure that the study can be completed in the proposed timeframe. Since the intervention is testing an early integration strategy, teams that already have such a program in place (albeit different from what is proposed) will be excluded to limit contamination.

The participating sites meet the above-mentioned requirements (Table 1): Dana-Farber/Boston Children's Cancer and Blood Disorders Center (DFBCC), coordinating center, Children's Hospital of Philadelphia Cancer Center (CHOP), (PI: Feudtner), Ann and Robert H. Lurie Children's Hospital of Chicago (PI: Waldman), Seattle Children's Hospital (SCH), (PI: Rosenberg), and Texas Children's Hospital (PI: Kang). These clinics are considered among the largest pediatric oncology programs in the country and their PC teams are part of the Pediatric Palliative Care Research Network co-led by Drs. Feudtner (Co-I) and Wolfe (PI). In addition, Deakin University, in Australia (PI: Orellana), will be home to the study's statistical team, and thus, a non-recruiting site.

Table 1. Characteristics of Heme/Onc and PC teams at each participating site

Site	Oncology Service Characteristics		Palliative Care Service Characteristics					
	No. new patients/yr	Interdisciplinary Primary Oncology team (MD, NP, SW)	No. new consults/yr	With cancer	No. of Providers			
					MD*	NP	SW	Other
DFBCC	500	yes	201	25%	6	2	2	6
SCH	280	yes	260	31%	4	1	3	4
CHOP	450	yes	180	19%	7	2	2	8

*Board Certified Hospice and Palliative Medicine physicians

References: *DFBCC*: Dana-Farber/Boston Children's Cancer and Blood Disorders Center; *SCH*: Seattle Children's Hospital; *CHOP*: Children's Hospital of Philadelphia Cancer Center; *MD*: Medical Doctor; *NP*: Nurse Practitioner; *SW*: Social Worker

2.2. Eligibility Criteria

2.2.1. Participants

We will recruit consecutive patients along with their parents until the target sample is reached. One parent will be responsible for answering the parental component of the study. Parent-patient dyads will be selected from the *base population* of **patients** that are:

- ≥ 2 years old,
- receiving routine (ongoing) cancer care at one of the participating sites, AND
- not off cancer-directed treatment and in remission.

All patients from the base population will be screened and included if the dyad meets the following criteria:

- o Patient has advanced cancer defined as: at least a 2-week history of progressive, recurrent, or non-responsive cancer of any type, or any brainstem tumor, or a grade IV Glioblastoma Multiforme, or decision not to pursue further cancer-directed therapy, OR any other progressive/recurrent brain or solid tumor, AND
- o is palliative care naïve, defined as the palliative care team not currently integrated into their regular oncology care. We will include participants with ≤2 prior contacts with the palliative care team if these occurred ≥2 months ago and no plans for continued follow-up are in place. For those with >2 prior encounters with the palliative care team we will include them if there has been no follow-up by the palliative care team in the past 6 months.

Parent-patient dyads will be excluded if any of the following apply:

- the patient,
  - o is older than 18 years of age and none of his/her parents are involved in his/her care, OR
  - o has a non-brainstem low-grade glioma with localized progression/relapse only, OR

  - is expected to receive a stem cell transplant within 18 weeks of enrollment, OR
  - is not expected to survive at least 2 months after enrollment; OR
- both parents,
  - are foster parents who do not have legal guardianship, OR
  - do not speak English or Spanish, OR
  - are unable to understand and complete surveys.

2.2.2. Rationale for eligibility criteria

The base population is defined to ensure that participants have a reasonable risk of presenting symptom distress. The lower age limit and language limitations are related to the age-range and languages covered by PQ validated surveys. The rationale for the upper age exclusion criteria is that the intervention focuses on the patient-parent dyad, thus if parents are not involved in care, most study outcomes cannot be measured. Disease related inclusion criteria were adapted from pediatric PC referral criteria recommended by Center for Advancement in Palliative Care.[66] Additional criteria are in place to ensure that participants are not already currently exposed to PC, and meet legal requirements for consent. Similar to prior studies,[37] this population will mostly be *early* in the advanced cancer course since they are being recruited primarily when cancer has progressed beyond initial treatment and a majority will live beyond one year. We chose to exclude patients who have a localized relapse or progression of low-grade gliomas because of their relatively stable clinical course, with low probability of having significant symptoms and quality of life impairment (we still will include those with localized benign tumors if they are localized in the brainstem); in addition, we propose to exclude patients who are expected to have a stem cell transplant and those that are knowingly close to end-of-life because of their distinctive symptom and quality of life trajectory, which may introduce significant heterogeneity into the sample affecting our ability to interpret study results.

2.3. Interventions

2.3.1. Description of Interventions

Following consent and run-in period, responders will be randomized (1:1) to PediQUEST Response (intervention) or usual cancer care (control) arms (week 0). The PI and Project Manager may together, rarely, decide to randomize a dyad that does not strictly meet the "responder" criteria, if, for example, there were technical or other issues that reasonably prevented them from becoming responders. Participants without home internet (<10%) will be provided a tablet with cellular service during the study.

If participation in the run-in period needs to be paused for any reason, resulting in delayed randomization, participants will be notified and allowed to continue or restart on the study with PI approval.

Figure 5. PediQUEST Response Intervention components, proposed mechanisms of action, and relationship with study aims

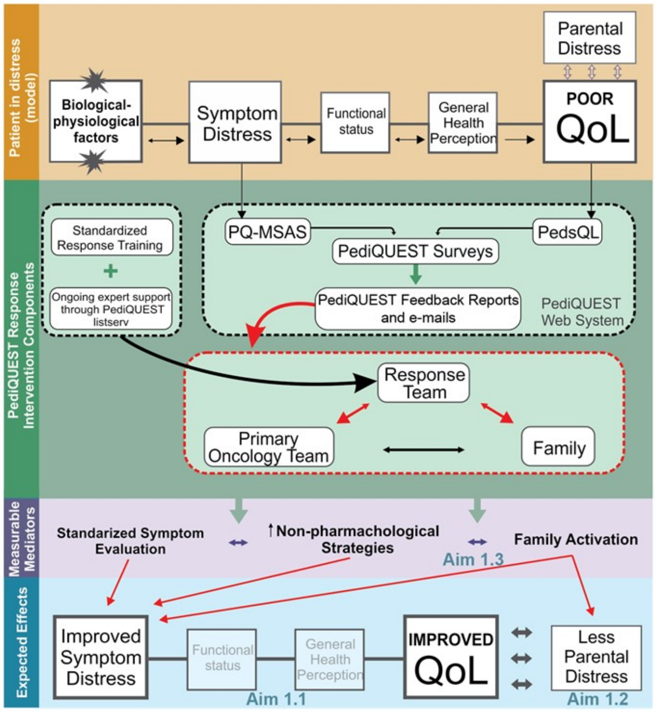

Abbreviations: QoL: quality of life; PQ-MSAS: PediQUEST-Memorial Symptom Assessment Scale; PedsQL: Pediatric Quality of Life Inventory 4.0™

2.3.1.1. PediQUEST Response Intervention

PQ Response is a multilevel intervention that will activate the symptom management process through two core components (see Figure 5 and section 1.1.2 for details on intervention rationale):

PQ web system (Surveys + Reports + email). The PQ web system can be accessed via any electronic device via web or mobile application (App). *After randomization*, families assigned to the intervention will have access to the full system which consists of: (a) PQ-Surveys: measuring symptom burden and HRQoL (see section 2.5.1 for instrument details and respondents for each section). Weekly surveys are automatically assigned and sent 48 hours prior to participant's usual clinic day (research coordinators (RC) can also manually assign a survey if needed); once a survey is assigned, automated reminders (email or App notifications) are sent daily for two days; after 48 hours, unanswered or incomplete surveys are auto-submitted; (b) PQ-feedback report (see Figure 6 and Appendix-Intervention Materials Section): generated automatically after a PQ Survey is answered. The report provides a graphic summary of up to three-months of patient HRQOL and symptom scores, the level of distress for each symptom (low, moderate, high, severe, extreme) and respondent (patient or parent; a brief summary section compares current findings to the prior survey; patient self-report –in children older than 5 years of age—is encouraged (in prior study, self-report was achieved in over 95% of the surveys); in the case of young children (no PQ-Survey available for self-report in children <5 years old, versions for children aged 5 to 12 are shorter) or whenever they do not want to answer, parent proxy responses will populate/complement the report; (c) PQ emails/notifications: once a report is generated a pdf of the report is automatically emailed or sent to the App of designated recipients, including the child if older than 8 years, parents, primary oncology team (including doctor, nurse practitioner and psychosocial clinician), and a designated Response team

member, usually nurse practitioner. The email subject line/notification text will state that a new report from the participant is available and the email body will include a copy of the report's summary and a link to the full report. PQ reports and emails are the vehicle for a patient-mediated activation of the symptom management process.

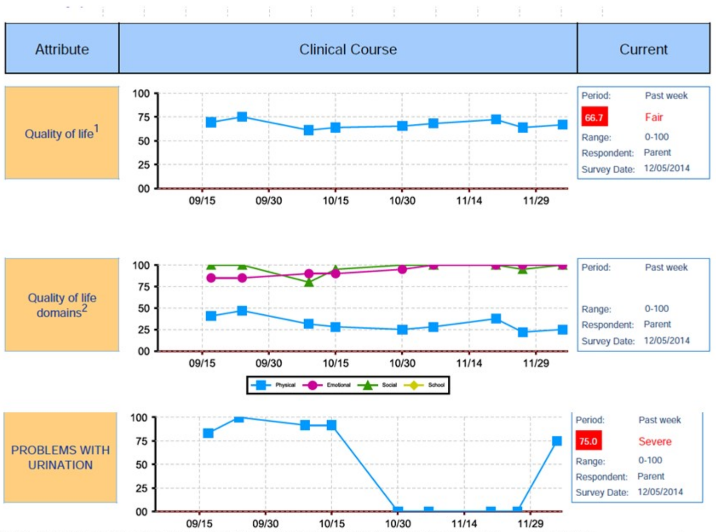

Figure 6. Partial view of a PediQUEST web Feedback report (mock). View of HRQOL scores and one severe symptom. Reports are e-mailed to families and providers immediately after participant completes online survey. Graphs display data from past 3 months. All symptoms reported as present in the last month are included to allow monitoring. A summary section at the end highlights main findings and changes.

Early Integration of the Response Team: In addition to the PQ system, those in the intervention arm will also receive oncology-PC integrated care through the Response team. Clinicians who typically provide PC consultation when invited by the oncology team will serve as the "Response team." All participating Response teams will receive intervention-specific training before the study begins (see below Support for Response teams). *Activities (Figure 7)*: It is recommended that the Response team conduct an initial consultation with the family within 3 weeks of randomization. This consultation will take place either at a hospital location (clinic or ward) or virtually via secure videoconference. Before the consult, a designated medical and psychosocial clinician of the Response team will contact the primary oncologist and psychosocial clinician (phone or face-to-face and when necessary by email) to inform them that the team will meet the family, collect data about the family's history and find out if there are any specific needs with which the Response team can help. During the initial meeting with the family, the Response team will begin with introductions (handout "Your child's quality of life matters", see Appendix-Intervention Materials), identify symptom and HRQoL history and current symptom issues, carry out *parent activation activities* (focused and tailored discussion on attitudes, beliefs and behavior regarding symptoms, their management, and impact on HRQoL; strategies to hold this discussion will be taught during training sessions), provide contact details, and explain to families the proposed follow-up plan; the consult will be documented using site specific documentation practices. Smart text will be provided to ease the capture of process measures. The initial visit will be scheduled using the routine PC team consultation scheduling system at each site (through PC team administrator or equivalent) at a time that is convenient for the family and does not involve an extra visit to the hospital/clinic; the RC will provide information about participants to make scheduling possible. Thereafter through week 16, the Response team will be encouraged to schedule regular meetings with family (at least monthly) to provide interdisciplinary consultative care focused on symptom and quality of life assessment, prevention and treatment that can be delivered directly or through primary team clinicians. Strategies will include, as appropriate, a standardized symptom assessment based on PQ e-PROMs, a multi-step treatment plan including recommendation of evidence- and expert-based pharmacological and non-pharmacological strategies (e.g. complementary therapies and psychosocial services, when indicated), and continued family activation strategies including working with families to increase awareness about existing or potential distress, and transfer of symptom

management skills (especially non-pharmacological strategies). Response teams are expected to maintain fluid and direct communication with the primary oncology team (P.O.T.) via email, phone or face-to-face to discuss their findings and recommendations. Ultimately, the P.O.T. will hold the decision to implement recommendations. Whenever recommendations are made (by P.O.T. or Response team) a designated member of the Response team will follow-up with the family to assess results and make further recommendations/contact P.O.T. as necessary. In the event that after following the proposed plan, there is a lack of adequate response (increase or no change in symptom scores or indication by patient or parent that relief is not sufficient), Response teams are encouraged to meet with local PIs to discuss the case. In addition, the confidential PediQUEST listserv will be available for use by Response team members (see below in Support). All communications and visits will be documented in charts. Once the proposed follow-up is completed, participants in the intervention arm will be offered the opportunity to continue being followed by the PC team (it will be clearly explained that use of PediQUEST web will be discontinued at this point).

At each site, research staff and the Response team will complete a schema to identify Response team members in charge of different aspects of the intervention. Specifically, the schema will indicate who will be alerted about new participants, who will receive the notice to schedule an initial visit, who will be responsible for initiating conversations with the P.O.T., who will receive PQ reports and distribute them to other team members, and who will be in charge of patient follow-up when recommendations are made. We expect that there will be two people in direct contact with the RC, one with a stronger administrative role and one with a clinical role (Figure 7). However, team members can be responsible for one or more tasks, structure may vary according to the organization of PC teams, and should be regularly revised to accommodate team rotation or other changes.

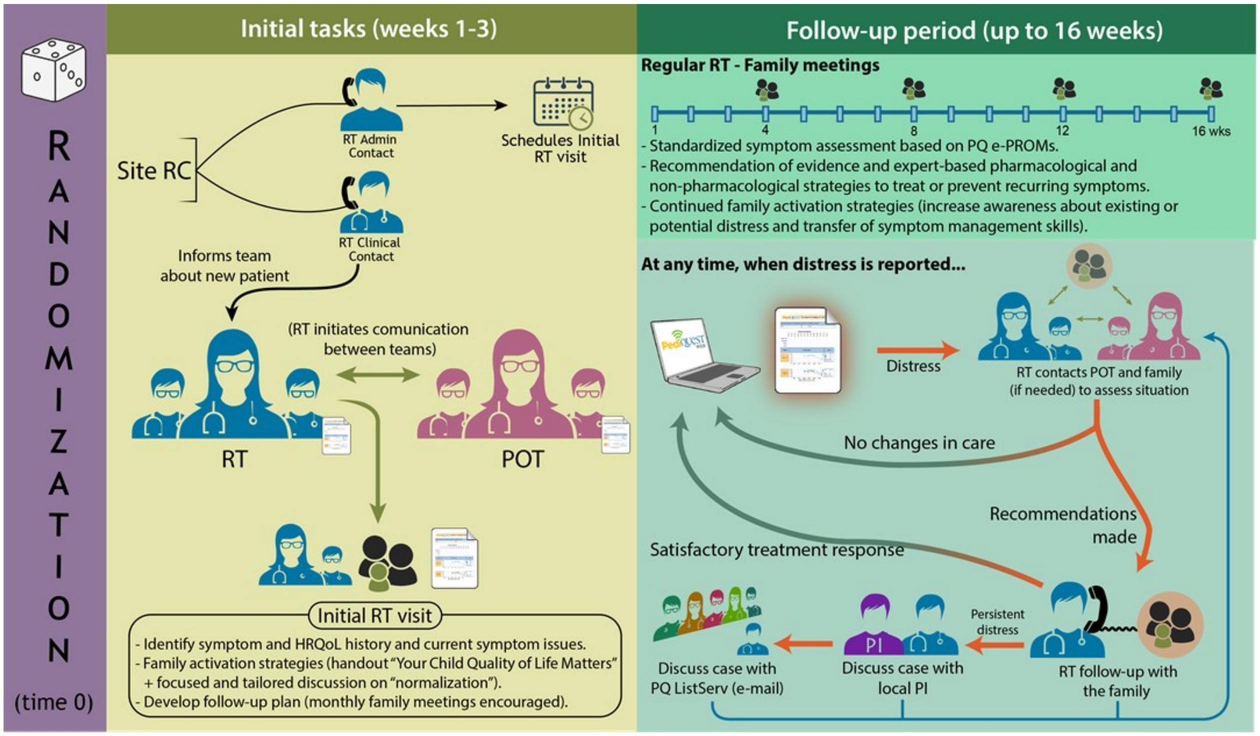

Figure 7. Early Integration of the Response Team Activities. Main activities of early integration component are depicted in the figure. *References*: *RC*: Research coordinator; *RT*: Response Teams; *P.O.T.*: Primary Oncology Team; *PQ e-PROMS*: PediQUEST electronic patient reported outcome measures; *Local PI*: Site Principal investigator; *PQ ListServ:* PediQUEST Listserv (expert listserv).

Support for Response Team: Training and Expert Consult. Response teams will receive a 48-hour online refresher course covering symptom management strategies. We will use material from the Education in Palliative and End-of-life Care for Pediatrics curriculum (EPEC-Pediatrics) developed by Co-I Friedrichsdorf and PI Wolfe.[67] Twelve online EPEC-Pediatrics modules with structured, brief evaluation covering prevalent physical and psychological symptoms will be accessible over the data collection period. Online training will be complemented with a 1-day site-based training that will cover study procedures, team work, and family activation strategies (See Appendix-Intervention Materials, for training agenda). Per the EPEC Pediatrics curriculum, case-discussion and role-play techniques will be used during the face-to-face training to evaluate skill acquisition. Study investigators Wolfe, Friedrichsdorf, Dussel, Baker, who are all EPEC-Pediatrics Master Facilitators, will be responsible for the face-to-face training. In addition, regular meetings of the active Response team members with local PIs are strongly encouraged. The goal of these meetings is to maintain open discussion about the active cases and ensure that therapeutic plans are updated. In addition, a PQ Response listserv including an interdisciplinary roster of national PC experts (see Appendix-Intervention Materials, for expert's roster) will be available to all Response team members and could be used at any point in the care of a study participant. Of note, listerv members will be instructed to comply at all times with HIPAA's privacy and security rules when exchanging patient information. PQ Response listserv member guideline will follow the American Academy of Pediatrics Pediatric Hospice and Palliative Medicine (PHPM) LISTSERV® posting guidelines and the American Academy of Hospice and Palliative Medicine "Connect" Access Agreement.

2.3.1.2. Usual Cancer Care

Participants randomized to the control arm (week 0), will receive the **usual cancer care** provided at the participating sites and will complete **weekly PQ-Surveys**. Participants in this arm will not have access to the full PediQUEST system, i.e. no reports will be generated. They will not meet the Response team but can receive regular palliative care consultations following the site's usual referral procedures. In general, children with cancer at participating institutions are cared for by a primary oncologist (attending physician and/or nurse practitioner), inpatient and outpatient nurses, and have access to psychosocial clinician care throughout the illness course. PC referrals are typically made at the discretion of the primary oncologist, often closer to end-of-life. For example, at DFCI during FY15, of all cancer deaths (n=64), 64% were consulted by the PC team, and the first contact occurred a median of 90 days before death. We have not found substantial differences in the way disease-directed, supportive, and palliative care is provided across participating sites.

2.3.2. Adherence

We plan to carefully monitor intervention fidelity following NIH Behavior Change Consortium's recommendations.[68] Indicators will measure successful provider training, Response teams' adherence to study procedures, and participants' adherence. The full plan to monitor fidelity has been developed during the initial phase of the grant by the Study Data Center, and has been discussed with the Data and Safety Monitoring Board. The final list of indicators of intervention fidelity is presented below in Table 2. The sources we will use to build the indicators are the patient's medical records and PediQUEST Reports. To aid the Response Teams documentation processes, a set of smart text phrases will be provided so that they can be used when completing notes.

Response teams' adherence to intervention delivery will be promoted during the initial training session and thereafter, during their regular meetings with local PIs (section 2.3.1.1). Key intervention points will be monitored by the PIs and

the Data and Safety Monitoring Board to aid local PIs in this task. In addition, participant PC teams will receive a monetary incentive equivalent to $1,500 per subject assigned to the intervention. Teams will be given the choice of receiving incentives as a lump sum (for the group) or divided proportionally among team members. Site PIs proposed that providing the choice may increase motivation. Team incentives are based on activities to be performed beyond usual care, including PediQUEST Response training, review of weekly patient PediQUEST reports (estimated to be 2-3/week during study period), augmented communication with primary oncology teams and families and, participation in meetings with local PI and use of PediQUEST ListServ (estimated to take place 2 times/month).

Participants' adherence to study procedures will be facilitated by a relatively short follow-up, flexibility in scheduling interviews, and working with experienced RCs (in at least two of the sites the RCs have already worked in prior or related projects of this group). Adherence with the PQ system will be enhanced in both arms through the automated emails/notifications sent to participants reminding them to fill in their assigned surveys. In addition, when a participant/family does not answer a PQ-Survey or declines to meet with Response team, RCs will contact the family over their preferred route of contact (phone or email) to discuss any difficulties and their willingness to continue participation. As a token of appreciation, the patient and one parent will each receive a $40 Amazon gift card for every 4 weeks of study participation. $20 dollars will be provided to patient and one parent for participating in the two-week run-in period. Participants' adherence will be monitored by the PM during study meetings (recurrence of these meetings will be flexible, with at least a monthly meeting). If either parent or child misses four consecutive PediQUEST surveys, the dyad will be removed from the study. Rare exceptions to this criterion may be granted at the discretion of the PI and PM.

Table 2. Measures of Intervention Fidelity

Process		Indicators	Source	Period	Target
Intervention component	Initial RT meeting	1. No. Times Visit Occurs within 3 wks randomization/ No. randomized to intervention	MR*	Monthly	>90%
		2. No. Times Symptom history addressed* in initial or next visit / No. randomized to the intervention			>90%
		3. No. Times "Your Child QOL matters" material handed to family and discussed* / No. randomized to the intervention			>80%
		4. No. Times RT communication with primary team documented*/ No. randomized to intervention			>90%
	Follow-up	1. No. of contacts of RT w/family or POT after a report of distress/ No. episodes of distress/month	MR + PQ Reports	Monthly	>80%
		2. No. of RT notes containing at least 1 recommendation (treatment or preventive, pharmacologic or non-pharmacologic), or otherwise providing the basis for not recommending anything / No. of distress episodes/month	MR + PQ Reports		>90%
		3. No. Times PC team communication with primary team documented/ No. episodes of distress/month	MR + PQ Reports		>75%
**Response Team Training and clinical support**		No. Providers with online + face-face training + evaluation/Total No. Providers/ site	Training logs	Once	>80%
		No. consults to Listserv /No. episodes of persistent distress/month	PQ Reports + Listserv logs	Monthly	>80%

Abbreviations: MR: Medical record; RT: Response Team.
*The Study Data Center will provide a set of smart text phrases to facilitate the documentation of intervention processes of local Response teams on the medical record. All palliative care notes from patients in the intervention arm will be abstracted and saved as pdf. Indicators will be built using pdfs.

Qualitative fidelity evaluation: All patients and parents, and a convenient sample of providers will be invited to participate in *exit interviews* that will inform fidelity assessment *(see section 2.5.3)*. Providers selected to do these exit interviews will receive a $50 gift card.

To monitor intervention fidelity, site RCs will collect all palliative care notes on intervention arm participants on a monthly basis, convert them to pdf, and send them to the PM using a send secure system (or a shared secure server such as Dropbox). The PM will receive monthly reports on the number of distress episodes reported through PediQUEST, among patients in the intervention arm, collect information on the use of listserv, and communicate with local PIs on an as needed basis to clarify information. With this information, the PM and VD (co-I) will build the indicators

and send monthly progress reports to the DSMB and SC (see section 5.1.2). Reports will be analyzed by the Data and Safety Monitoring Board to detect patterns of non-adherence or protocol deviations. If deviations in intervention processes are detected, they will be discussed with the local PI to decide whether retraining or site visits are indicated.

2.3.3. Rationale for intervention's "dose," frequency, and administration:

Weekly PQ-Surveys: Surveys will be administered weekly to capture distress in real time. A longer interval may make the intervention less relevant if surveys miss the distress event or participants answer about distress that occurred longer ago. Ideally, one would want participants to report distress on an "as needed" basis, however this approach is challenging from a design point of view as controls may have less incentive to report. For this reason, we decided to collect weekly measures in both arms. Weekly reporting allows the Response team to intervene and propose prevention strategies if symptoms are thought to be recurrent. During the pilot, adherence to weekly surveys was good; over 75% of participants answered 50% of weekly surveys or more, and 60% answered 75% of weekly surveys or more.

Response team intervention: Response teams will contact the families: (i) on a regular basis over the intervention period (we encourage at least monthly encounters), (ii) to attend to PQ reports, and (iii) to monitor treatment recommendations. Regular contacts are proposed as a means of standardizing the intervention across patients with varying experiences of distress.

Response teams will be informed of criteria for potential distress including:

a. a PQ-MSAS total score of 3.2 or higher (for children using PQ-MSAS 7-12 (see section 2.5.1.) the equivalent threshold value is 9) or individual symptom scores of 50 or more, or
b. PedsQL total score below 60.

A PQ-MSAS score of 3.2 (or 9) was chosen as a threshold because it is the minimum score that includes one severe symptom (i.e. that participant reported at least two of the following characteristics for a given symptom: it is very severe, or happens almost all the time, or bothers him/her a lot or very much). It could also represent a report with five mild or moderate symptoms, which also warrants evaluation. In addition, teams will be advised that any symptom score of 50 or more may indicate current or past distress. Similarly, the score of PedsQL of 60 denotes a poor quality of life (this represents approximately one standard deviation below the average PedsQL score in pediatric cancer populations). These threshold scores will promote optimal Response team intervention. Further, this approach helps standardize the response to distress and as such increases replicability of the intervention.

2.4. Outcomes

2.4.1. Primary Outcome Measures (aim 1)

Child Quality of Life: Difference between the two treatment arms in the mean over 16-weeks (16-w) of PedsQL total score as reported by (a) the parent for all enrolled patients, and (b) the patient if 5 years of age or older. PedsQL scores range from 0-100 (higher better) and are calculated as the average of all 22 items (total score). For each weekly administration, scores are calculated if >50% of the PedsQL items are answered.[69] Treatment success will be defined as a difference of at least the minimal clinically important difference (MCID) between groups. The MCID for PedsQL total score is 4.4 points.[52]

2.4.2. Secondary Outcome Measures

2.4.2.1. Patient-level outcomes (aim 1)

Child Quality of Life: Difference between the two treatment arms in the mean over 16-weeks (16-w) of PedsQL sub-scale scores (physical and psychosocial) as reported by (a) the parent for all enrolled patients, and (b) the patient if 5 years of age or older. PedsQL subscale scores are calculated as the average of 7 physical items and 15 psychosocial items respectively. The same scoring ranges and rules as explained for total score apply.[69] MCID for PedsQL for the physical and psychosocial subscale scores is 6.7 and 5.3 points respectively.[52]

Symptom burden scores: Difference between the two treatment arms in the mean over 16-w of PQ-MSAS total and sub-scale scores (PHYS and PSYCH sub-scales) as reported by (a) the parent of all enrolled patients and (b) the patient when 13 years old or older. PQ-MSAS scores range from 0-100 (lower better) and are calculated as the average of all 26 individual symptom scores (total score), 8 physical and 6 psychological items for the sub-scale scores. Individual symptom scores are derived as the average of each symptom's reported frequency, severity and extent of bother scores. For each weekly administration, PQ-MSAS scores are calculated if >50% of the symptoms are answered.[53] As mentioned in 1.3.2, PQ-MSAS has an age-adapted instrument from the age of 7 (PQ-MSAS 7-12). However, as compared to the PQ-MSAS 13-18 (self-report for teens) and the parent versions, PQ-MSAS 7-12 is much shorter (only asks about 8 symptoms) and uses a different response scale. The data on PQ-MSAS 7-12 will be collected and used for feedback reports but not for hypothesis driven outcome analysis; for this age group, only parent reported symptom burden will be analyzed. Child-reported data will be used to conduct exploratory analysis.

2.4.2.2. Parent-level outcomes (aims 2 and 3)

Parent Psychological Distress: Difference between the two treatment arms in the mean over 16-w of anxiety and depression scores measured on a monthly basis by S-TAI-State and CES-D-10; and on symptom-related stress measured at study entry and at week 16 by *a*RSQ-Stress. Scoring details are presented in section 2.5.1.

Family activation: Difference between the two treatment arms in (a) Change in active coping, planning, and instrumental support Brief-COPE scale scores between week 16 and baseline, ordinal variables; (b) Total No. and (c) No. of different complementary therapies used for symptom treatment (reported by parents every 4 weeks), and (d) use of psychosocial services, as measured by total No. of psychosocial clinician encounters documented in the medical record, over the 16-w study period.

2.5. Participant timeline

Participants' timeline is presented in Figure 8. Study participants will be assessed using the PediQUEST web system. At all sites, immediately following consent, the RC will help families register in the system and download the mobile App. All participating parents will be provided with a PQ account, linked to an email of choice. In the case of children aged 2 to 4 years old, only parents will answer PediQUEST Surveys. In the case of children aged 5 to 7 years old, parents and children answer PediQUEST Surveys which are sent to the parent's account; it is expected that parents read the questions out loud to the child. Patients ≥8 years old will be provided with a PQ account of their own (will need a different email than the one provided by their parent) and will answer PQ-Surveys on their own; in addition, their parents will answer proxy versions (see section 2.5.1). Once a PQ-Survey is assigned, a link is sent by email/notification to the corresponding PQ account owner. In addition, questionnaires can be handed over to participants using the research team tablet for completion, or answered over the phone (administered by the RC) if the participant prefers. PQ Reports will only be available for participants assigned to the intervention arm during the 16 weeks after randomization. After registration, the RC will manually assign the *Baseline Packet* (30 minutes, online parental survey, preferably answered at the clinic or ward at the time of enrollment). Over a total of 18 weeks (2-week run-in period and 16-weeks post-randomization follow-up), parents and patients ≥5 years old in both arms will be automatically assigned weekly *PediQUEST surveys* (10 minutes each, online self-administered surveys answered from any location, provided there is access to internet connection). In addition, parents of both arms will be assigned *Monthly Parent Questionnaires* evaluating distress, burden, and use of complementary therapies every 4 weeks (15 minutes, online surveys answered independently), beginning at the time of randomization as indicated in Figure 8. *Parent Activation Surveys* will also be administered at weeks 8 and 16 (the measure at week 8 will be used for an exploratory mediation analysis described in the analysis section 4.3.2.5). *Medical records* will be abstracted by RCs every four weeks. All patients ≥8 years old and parents who completed the 16-w follow-up, dropped out, and parents of children who died, and a convenient sample of providers, will be invited to participate in an *Exit Interview* (in-depth face-to-face or phone interview) administered by the RCs to evaluate study processes, care experience, and intervention fidelity (among those in the intervention arm). There are two points in the timeline with potential for delays. Technical issues, such as troubleshooting access to PediQUEST with participants, or other technical mishaps (which we expect to be rare events), may prolong the run-in period by one or two weeks. In addition, the scheduling of exit interviews will always occur out of the 16-week follow-up period, most frequently within the next week after the last survey is answered, but adapting to the family availability. When a patient dies within the study, the invitation for the exit interview, as explained below, is sent 12-weeks after the death. For these reasons, total enrollment time will be 18 weeks in most cases, but there will be exceptions. This will be explained during the enrollment process.

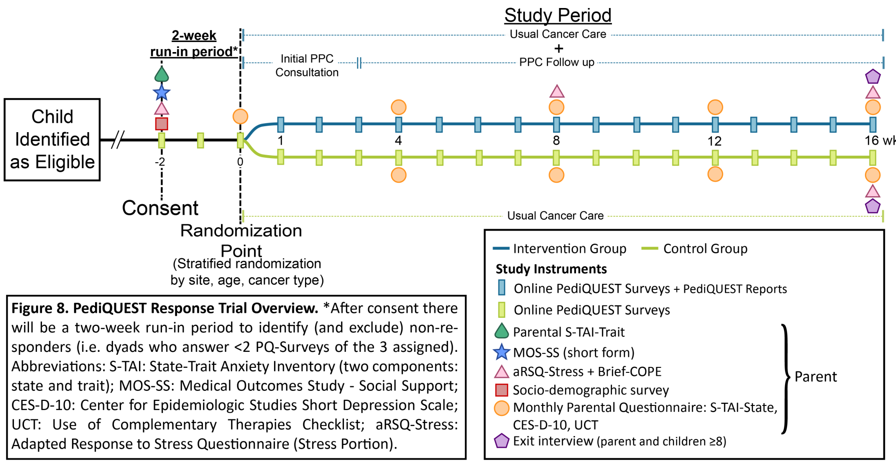

Figure 8. PediQUEST Response Trial Overview. *After consent there will be a two-week run-in period to identify (and exclude) non-responders (i.e. dyads who answer <2 PQ-Surveys of the 3 assigned). Abbreviations: S-TAI: State-Trait Anxiety Inventory (two components: state and trait); MOS-SS: Medical Outcomes Study - Social Support; CES-D-10: Center for Epidemiologic Studies Short Depression Scale; UCT: Use of Complementary Therapies Checklist; aRSQ-Stress: Adapted Response to Stress Questionnaire (Stress Portion).

2.5.1. Study Instruments (see Appendix for sample surveys)

Table 3 below shows study instruments that will be administered to all study participants, unless otherwise indicated. Most tools were used in prior studies or are otherwise extensively validated (e.g. parent distress and HRQOL). Information about instrument's psychometric properties is provided when available. All instruments have English and Spanish versions. During the pilot, parent and child burden from completing study instruments was evaluated as minimal.

Table 3. Study Instruments

Baseline Packet (week -2, right after consent)			
Most baseline tools will help characterize the population attended.			
**Respondent**	**Construct**	**Instrument**	**Description**
**Enrolled Parent**	**Demographics**	Demographic section of the Survey about Caring for Children with Cancer (SCCC), originally developed by study investigators[37]	Age, gender, race, marital status, number of siblings (of participant child), religion, religiousness, education,zip code.
	**Household Material Hardship**	Household Material Hardship Survey[70]	Self-report family income, child's health insurance, health literacy, evaluation of household hardship (housing and transportation, utilities, food insecurity, and financial strain)
	**Social Support**	Medical Outcomes Study Social Support-Short Form[71]	4 domains: emotional/ informational, tangible, affectionate, and positive social interaction; 8-items. High reliability (α>0.91) and stability over time.
	**Anxiety**	Spielberger's State-Trait Anxiety Scale (S-TAI)-Trait subset[55]	Measures trait anxiety (20 items); evaluates relatively stable aspects such as calmness, confidence, and security. Response options: frequency of feelings "in general:" 1) almost never, 2) sometimes, 3) often, and 4) almost always.<br>Scoring: Item scores are added. Scoring is reversed for anxiety-absent items. Range of scores for S-TAI-Trait is 20–80 (higher indicates greater anxiety). Validity and reliability are high. High stability over time.
	**Symptom-related stress**	Stress portion of the Response to Stress Questionnaire-Pain(aRSQ-stress)[57,72]	Adapted version including 11 items that evaluate parental stress in past month related to uncertainty, meaning, treatment adherence, and effects on daily life of child symptom distress; and 1 item assessing perceived control over these problems.
	**Activation**	Brief-COPE[58]	Adapted version (15 items) of the Brief-COPE scale used to evaluate parent's use of five coping strategies relevant to symptom management using two items each: active coping, planning, instrumental support, acceptance, and self-blame. Five additional items will evaluate emotional support, religion, positive reframing, behavioral disengagement, and denial. Scores on each scale (or item) range from 2 to 8, with higher scores indicating greater use of that strategy. Use of selected subscales encouraged by author. Use of single items decided after piloting instrument and finding no variability with using the 2-item scales.

PediQUEST Survey (weekly, from week -2 to week 16)

For these aims we will use the English and Spanish **PQ-Surveys** which have 5 versions (respondent- and age-adapted) that collect **both** parent and child reports (child from the age of five).¥ Children will answer the same survey version throughout the 18 weeks according to the age at time of enrollment.

Instruments included	PQ-Memorial Symptom Assessment Scale (PQ-MSAS)[19,53,54,73]			Pediatric Quality of Life Inventory Generic Core Module (PedsQL 4.0™)[51,52]							Overall Well-being[37]		
Construct measured	Symptom Burden			HRQOL							General Health Perception		
Instrument Characteristics	Measures presence, severity, frequency, and extent of bother in past weeks for 26 physical, psychological and 3 "other" symptoms			Assesses how much of a problem has each item been in past week (evaluates physical (7 items), emotional, social, and school (5 items each) performance)							1-item ("Overall, how have you been feeling during the past week?" Anchors: not well at all; extremely well)		
Versions	Proxy (Parent)	Self-report		Proxy (Parent)				Self-Report			Proxy (Parent)	Self-report	
		7-12ƚ	13-18	2-4	5-7	8-12	13+	5-7	8-12	13+		5-6	>6
Response Types‡	L-5	L-4	L-5	L-5				FS-3	L-5		VAS	FS-3	VAS
PQ Versions 2-4													
PQ Versions 5-6													
PQ Versions 7													
PQ Versions 8-12													
PQ Versions 13+													
Validation data	high reliability (α=0.81)§			high reliability (α=0.93 parent and 0.88 child report)							Spearman Correlation with PQ-MSAS =- 0.56§		
Scoring range	0-100 (100=worse)			0-100 (100=better)							0-100 (100=better)		

¥The collection of both parent and child data will be used only to analyse concordance. PediQUEST reports only provide feedback on child answers (or parents when children are young or do not want to answer); ƚ PQ-MSAS 7-12 evaluates shorter time frame (past two days) and only 8 symptoms; ‡Response types: L-5: 5-option Likert type; L-4: 4-option Likert type; FS-3: 3-option Faces scale; VAS: Visual analogue scale (100 mm); §Data from PQ RCT

Monthly Parent Questionnaires (weeks 0-4-8-12-16)

Respondent	Construct	Instrument	Description
Enrolled Parent	**Anxiety**	Spielberger's S-TAI-State subset[55]	State-anxiety (20 items) (sensitive to transient changes) measures presence and severity of current symptoms of anxiety. Scoring: Item scores are added. Scoring is reversed for anxiety-absent items. Range of scores for S-TAI-State is 20–80 (higher indicates greater anxiety). Suggested cut-point: 39-40. Validity and reliability are high (α=0.86-0.95)
	**Depression**	Center for Epidemiologic Studies– Short Depression Scale (CES-D-10)[56]	Assesses frequency of occurrence during the past week of depressive symptoms (10 items). Response options: (0-3): Rarely or none of the time (less than 1 day); Some or a little of the time (1-2 days); Occasionally or a moderate amount of the time (3-4 days); Most or all of the time (5-7 days). Scoring: Item scores are added (for two positive items scores are reversed). Score is not calculated if <9 items answered. Range of scores is 0-30 (higher indicates depressed mood). Suggested cut-point: 10. High reliability (α=0.88-0.91)
	**Symptom-related stress**	aRSQ-Stress[57,72]	See above. Measured at weeks 8 (for exploratory analysis see 4.3.2.5) and 16
	**Activation**	Brief-COPE[58]	See above. Measured at weeks 8 (for exploratory analysis see 4.3.2.5) and 16

		Use of complementary therapies checklist	A*d hoc* checklist of complementary therapies; parents will report use (yes/no) in past four weeks and reasons for use.

2.5.2. Medical Record Abstraction

The following variables will be abstracted from the medical records at week -2 (study entry): child diagnosis, cancer-directed treatments, and symptom treatments.

From week 0 to 16 (i.e. randomization point to end of follow-up), information will be abstracted every four weeks by RCs summarizing events that occurred in the four-week window including: disease status (stable, progression, remission), date of change in status when appropriate, cancer-directed and symptom treatments, formal PC referrals, hospitalizations, No. of psychosocial clinician encounters and No. of PC encounters documented. Chart abstraction forms will be entered into PediQUEST web.

2.5.3. Exit interviews

Children 8 years old or older who completed data collection through week 16 (end of follow-up) or dropped out and their parents, and parents of patients who died will be invited to participate in an exit in-depth interview to understand more about their experience in the study. The interview will be administered by the RC by phone, or face-to-face, and audio-recorded. The exit interviews will ideally take place within a week of answering the last PediQUEST Surveys. If this is not feasible, RC will schedule it at the next most convenient time. In addition, a sample of approximately 18 oncology providers per site will participate in exit interviews after their patient completes the study. A convenient sample of PC providers will be interviewed throughout the study.

The goal of these interviews is to collect information that will help understand **processes (of the study and of care)** in both arms and **intervention delivery/fidelity in intervention arm,** as well as identify the "active ingredients" of the intervention, and potential for adoption and dissemination (See Appendix-Study Instruments). Key topics to be addressed: reasons for participation, burden/facilitators to keep participating, symptom processes, and in the case of participants in intervention arm, intervention delivery, and potential for adoption (characteristics, likes/dislikes).

When a participant communicates the decision to drop out, the RC will ask permission to record and conduct a brief exit interview mainly focused on understanding reasons for dropout. If participant agrees, will discuss about the other topics mentioned above (symptom processes and intervention delivery if participant was on intervention arm).

If the patient died during the study, the research coordinator will send a letter (see Appendix-Recruitment Materials section) to the parents 12 weeks after the death to invite them to participate in a phone or face-to-face interview. The letter will contain an opt-out card. Parents will be instructed to return the opt-out card if they are unwilling to participate in the survey. Parents who do not return the opt-out card will receive up to three follow-up telephone calls by an investigator, with a maximum of two phone messages left, starting approximately a week after the letter is sent and repeated weekly to ascertain whether or not they have received the letter. If they have not received the letter, the researcher will ask if they are willing to receive it and will verify the mailing address. If parents agree to take part, the RC will schedule a convenient time to

conduct the exit interview and discuss whether they prefer to do it by phone or in person (at the clinic or hospital). Parents who do not send back the opt-out card and are unreachable by phone will be considered a refusal to participate. In the exit interviews, in addition to the key topics mentioned above, parents will also be prompted to provide their perspective on end of life care.

2.6. Sample Size

The target sample size (SS) is N=136 participants 68 in each arm. Texas Children's Hospital will have a slightly lower accrual goal of 34 total dyads, as opposed to 50 dyads, since they will collect data for a shorter period of time. Based on prior work,[38] we estimate that there will be approximately 32 eligible patients/site/year, representing a total of 288 eligible patients over the 36 months the study will be available for recruitment. Pilot data suggest an enrollment rate of 70% which translates into approximately 200 patient-parent dyads, our enrollment target. After the two-week run-in period, non-responders will be excluded. Assuming that about 25% will be non-responders, a conservative estimate based on pilot data, there would remain 150 patients to be randomized to either the intervention (n=75) or usual cancer care (control, n=75). Based on previous work we estimate an attrition of approximately 10%, mainly due to dropouts. We therefore expect to collect complete data on a minimum of 136 dyads, 68 per group, a sample size that achieves sufficient power for all outcomes.

2.6.1. Power considerations

Although the SS is mainly driven by pragmatic considerations (target population is small), power calculations were conducted and indicate that our projected minimal SS of 68 children per group provide sufficient power for both primary and secondary outcomes for clinically meaningful effect sizes. All power calculations presented below were computed with two sample independent one-side tests, α=0.05 unless otherwise specified. For Aims 1 and 2 power estimates are conservative because they only use one observation per subject (average across time) without accounting for the repeated measures within subject.

Aim 1 *HRQoL (primary) and Symptom (secondary) outcomes*: Standard deviations for these outcomes were estimated using data from our PQ study (39 weeks follow up, surveys answered when attending clinic or once a month).[19,39] For each child, we identified all 16-w periods with ≥6 surveys and calculated mean PedsQL total and mean PQ-MSAS total scores for each 16-w period. We randomly selected one period per child and estimated the standard deviation of 16-week PedsQL means (SD). Strategy was repeated 10 times and a pooled estimate was calculated. A sample size of 136 children (68 per group) achieves 85% power to detect a 4.4 point increase (MCID) in the mean over 16-w PedsQL Total score (SD for both groups 9.5), and a 95% power to detect a 3-point decrease in mean over 16-w PQ-MSAS total score (SD = 5.3). These power calculations are conservative as they are based on mixed responses coming from parents and children, while in the current proposal responses from different informants will be considered in separate analyses and are expected to have less variability.

Aim 2 *Parental anxiety (S-TAI-State), and depression (CES-D-10) (secondary outcomes)*: A sample size of 68 parents per group achieves more than 80% power to detect a moderate to large effect size (Cohen's d = 0.43). For example, this effect size would correspond to a difference of 5.2 points in mean S-TAI-State (SD=12.2).[74]

Aim 3 *Family Activation outcomes (secondary outcomes)*: Change in parent activation level between baseline and 16-w. The target sample size achieves 80% power to detect a change in any of the coping scale scores of size 0.5. This effect sizes correspond, for example, to a score change of 0.75 points between baseline and 16-w (estimating a SD = 1.5, consistent with literature reports).[75] The sample size has 82% power to detect a mean increase of 2 encounters with psychosocial clinicians between trial arms in 16-w (SD=4.5, from pilot data). Finally, for mean No. of CT used for symptom treatment over 16-w, our sample size has 80% power to detect an effect size of 0.43.

2.7. Recruitment

We will recruit consecutive patients at each of the outpatient oncology clinics or inpatient wards of the five participating sites or remotely. Based on prior work, we expect to enroll between 1-2 patients/site/month; considering that the target population is 23-34 patient-parent dyads/site and that enrollment rates will vary over time, we presume that the enrollment period will last approximately 27 months/site and the data collection period an additional four months. Recruitment will start in a staggered fashion, adding a new site every three months, ending with a total enrollment time of 36 months across the five sites (see section 7, Study timeline). We will enroll one parent per patient, as designated by the family, recommending that it be the parent who holds more of the primary caregiving role.

For all eligible patients, the recruitment protocol will be similar to that used in our prior prospective study and pilot.[38] At each center, RCs will generate a list of potentially eligible patients by reviewing clinic rosters and e-mail sign-outs on a daily basis, establishing direct communication with primary oncology teams, and attending clinic and tumor board conferences. When we reach a plateau in identifying new patients, clinic roster and sign-out screen will be conducted on a weekly basis. Study promotion activities will consist of short in-service meetings at study launch ((with additional meetings conducted later if recruitment is low).

To generate the list of potentially eligible patients, all patients belonging to the *base population* (see section 2.2.1), i.e. ≥ 2 years old AND seen by the outpatient oncology clinic or inpatient oncology service will be entered into a *pool list* excel file. The *pool list* file will have automated fields and allow for a quick pre-screening process that renders a patient either potentially eligible or not eligible. For all patients identified as potentially eligible, RCs will proceed to screening using the "Eligibility Form" in REDCap, and eligibility will be verified by reviewing patient's medical chart (Appendix-Recruitment Materials) and validated by a designated investigator. Investigator will send an email through REDCap to RC confirming patient's eligibility status. After eligibility is verified by the local PI, RCs will collect potential participant's contact information, race/ethnicity, English proficiency, and main diagnosis from the medical record. This data will be entered on the "Subject and contact information form" in REDCap to help the approach process and better understand selection bias. In order to monitor the identification and pre-screening process, every two months, the PI will review medical records of 5% of participants identified as not eligible during the pre-screening phase. If the error rate is larger than 5%, RCs will be re-trained and participants screened again.

A HIPAA waiver is requested for the whole identification process (see section 6.2.2). The RC will then email the primary oncologist (MD or nurse practitioner) to inform him/her that the RC will approach the patient during

the next visit, giving the provider the opportunity to refuse the approach (See email template in Appendix-Recruitment Materials). RC will register the provider opt-out process outcome in REDCap.

The RC will next approach the family when in clinic or on the ward, if the patient is admitted, and will provide a brief overview of the study, will hand the parent and patient age-appropriate study brochures (see Appendix- Recruitment Materials), and conduct a tablet demonstration of PQ web. If families are interested in participating, the RC will move forward with the consent process providing more details or schedule a convenient time to do so. If preferable for the family, this second conversation may take place over the phone and the family may bring a completed copy of their consent form to their next visit. If after being introduced to the study and having had the opportunity to ask questions, the child and parent(s) are willing to participate, the parent and patient if older than 10 years of age will be asked to review and sign the informed permission/ assent documents covering the patient's participation (more details in section 6.2). A separate consent document will cover caregiver's participation. If families do not make a decision right away, the RC will develop a follow-up plan with the family. All the approach process will be documented in REDCap's "Approach Form." If more than a week has elapsed, disease-related eligibility criteria will be re-verified.

In addition to in-person recruitment, the RC may also conduct the consent process remotely. For these procedures, email addresses, mailing addresses, and phone numbers will be obtained from the patient's medical record. Eligible participants will be mailed or emailed an information sheet that outlines the details of the study and elements of informed consent, along with study brochures and an opt-out opportunity. If a participant decides to opt-out, they will be prompted to answer a non-participant questionnaire within the document for opt-out instructions. If no opt-out occurs, the RC will then follow up with a phone call after a few days to ensure that the documents have been received and gauge the family's interest in learning more. If the family declines participation, they will be prompted to complete the non-participant questionnaire verbally over the phone. If the RC has maximized outreach attempts to enroll the family, at this point they will be classified as a passive decline and will be asked to complete the non-participant survey. These 'passive declines' will receive a unique opt-out document with language specific to their situations. If the family expresses interest in participating, the RC will provide details on the study, walk through the consent form to ensure that all elements of informed consent are provided, and offer the family the opportunity to ask questions. If the parent and child (when applicable by age) decide to participate, they will be sent the appropriate consent/assent documents to sign and return via mail or email. Alternatively, parents and children can also provide verbal consent/assent in place of written documentation of informed consent. In cases of verbal consent/assent, parents and children older than 18 will provide verbal consent. Children older than 10 but younger than 18 will provide verbal assent with the parent present, and the parent will provide verbal consent on behalf of the child. For these cases, we request a waiver of documentation of consent/assent for parents and children based on the following considerations: (i) participating in the study constitutes "no more than minimal risk"; (ii) the rights and welfare of the subjects will not be adversely affected, since parents and children will be given all elements of informed consent both in writing and verbally over the phone; (iii) increased study feasibility: we would like to minimize the burden that may result from returning a consent form via mail or email. Once a participant signs the consent, the RC will register participant in PediQUEST web, assign study status as "enrolled participant," create the corresponding PQ accounts as explained in 2.5, manually assign the Baseline Packet and baseline PediQUEST Surveys, and email the patient's primary oncology team. Study identification numbers have been generated by the Study data center and are embedded in each site's pool list. All participants will be registered in DFCI's Clinical Trial Management System Oncore following

procedures specified in DF/HCC SOPs REGIST-101 and REGIST-104. Randomization procedures are explained in the next section.

Strategies to enhance recruitment include a direct family approach, flexibility in scheduling recruitment interviews, and working with experienced RCs (in at least two of the sites the RCs have already worked in prior or related projects of this group). Some study characteristics such as its relatively short duration, facilitation of PediQUEST web's registration process by the RC, and the small non-monetary incentives provided (section 2.3.2), should also boost recruitment and retention rates. Finally, the advertising and in-service sessions with providers should help reduce any potential barriers to identify/refer patients to the study.

Should a participant reach the age of majority while participating in the study, study interventions will continue. We are requesting a waiver of documentation of consent for these patients given the consent documents and study procedures for adolescents and young adults are identical and adolescents must provide assent to participate. The continued collection of data during this period presents no more than minimal risk, and halting study procedures would likely lead to loss of valuable study data. An information sheet (appendix) which contains all elements of informed consent will be provided to participants who reach the age of majority at the earliest opportunity. If the participant is not scheduled for a clinic visit within a month of their birthday, the RC will call to review the sheet over the phone.

With the purpose of studying selection bias, and understand better the characteristics of the enrolled sample, i.e. how well enrolled patients represent the pool of eligible patients, we will collect vital status (Dead/Alive) at 6 months of all patients identified as eligible. For deaths of patients who were randomized, we will also collect date of death as it will be used in the exploratory analysis. We will also collect information on race, ethnicity, and English proficiency of all approachable families, including those that decline participation. Families that decline participation will be asked four structured questions on their race, ethnicity, and English proficiency and two brief open-ended questions to understand their reasons for non-participation and whether virtual methods constitute a barrier to participation (see Non-Participation Survey). These questions will be asked during the approach or emailed to families no later than 3 days after a declination or opt out. Families will be reminded that they can decline to answer any questions.

3. Methods: Assignment of interventions

3.1. Allocation (sequence generation, concealment, and implementation)

Participants will be randomly assigned to the intervention or control group with a 1:1 allocation as per a computer-generated randomization sequence created by study statistician, co-I Orellana, and stratified by center, age (2-7, 8-12, ≥13 years old), and type of cancer (hematological, non-hematological malignancies). Study personnel will not have access to the sequences. Random sequences will be embedded in REDCap by informatics personnel, ensuring best practice standards for allocation concealment. Two weeks after enrolling, at week 0, if participant meets the criteria of being a responder, i.e. having answered two or more PQ-surveys, regardless of who in the dyad answered them, the RC will be able to see study arm assignment in REDCap. The RC will begin intervention procedures by turning on the PQ-feedback report and email features of PQ web,

notifying the primary team and linking the account to the corresponding providers (primary oncologist, primary nurse, primary psychosocial clinician, and PC Response team key person) so they start to receive reports too, and letting the PC Response team administrative designated person know of the assignment so they can schedule the initial visit with the Response team (as explained in 2.3.1 and Figure 7).

3.2. Blinding

After randomization, participants, PC team, oncologists, and research staff will be unblinded to group assignment, because the intervention is not amenable for blinding.

4. Methods: Data collection, management, and analysis

4.1. Data collection methods

Research material for this study primarily involve patient reported outcomes and parent reports collected and stored electronically through the PQ web system. In addition, RCs will collect data for tracking purposes (with electronic (REDCap) and/or paper and pencil checklists and personal notes), do the medical record abstraction (online), and conduct the semi-structured exit interviews. Data collection protocols are identical for both arms and were described in section 2.5, participant timeline, together with instrument's psychometric properties. A sample of study instruments is presented in the Appendix.

REDCap is a web-based application that allows for the creation and management of online databases. We will run the study's enrollment tracking system and randomization module on this platform. PQ web is a web-based application that allows for the use of PROMs, includes scheduling, reminder, and scoring and feedback capabilities. The PQ Response's Baseline Packet, PQ-Surveys, Monthly Parent Surveys, and the Medical Record Abstraction will be collected through this system. Participants can use the PQ mobile application to answer surveys and view reports; providers can also download the App and use it to view patient reports. Both systems can handle multicenter studies and easy transfer to common statistical packages. The semi-structured exit interviews will be conducted face-to-face or over the phone and audio-recorded.

All participating sites will designate an RC who will be trained in all study procedures (9 hours including role play), participate in monthly meetings, generate reports of research activities, prepare and maintain IRB documentation, enroll participants, and be in charge of the communication with oncologists and Response teams regarding subjects' participation. Strategies to enhance retention have been described in section 2.3.2, adherence.

4.2. Data management

The Dana-Farber Cancer Institute in Boston will serve as the Study Data Center (SDC), and will be responsible together with the study Data and Safety Monitoring Board (DSMB) for providing a specific data collection and monitoring system, including the design of the Data Management Plan, Standard Operating Procedures (SOPs), Data Validation Plan, study data collection and completion guidelines for investigators, supervising data collection procedures, assuring maintenance of high quality databases, and arranging an efficient and safe

transfer of non-electronic study data, in compliance with Good Clinical Practices (GCPs). RCs and local PIs will be responsible for the local data centers (LDC) at each site and will communicate with the SDC on a routine basis. RCs will be trained in data management procedures and GCPs before starting enrollment.

Each eligible participant (child and parent) will be assigned a unique study identification number. REDCap will be used to collect data during participants' recruitment (see 2.7). Most study data will be collected and stored electronically through REDCap and the PQ web system. All other source documents, exit interviews' audio files and transcripts, *pool list* and other tracking forms, with the exception of consent documents, will be created as or converted to electronic format (e.g. audio files will be mpg4, transcripts of interviews .doc, palliative care clinical notes will be saved as pdf files). Data managers and study statisticians will always use de-identified datasets when generating study reports or conducting interim and final analysis. Details about data storage and how we plan to preserve subjects' confidentiality together with security features are explained in section 6.3.

A comprehensive tracking system will be implemented at the local and study data centers. Data quality reports will be generated on a weekly basis. Whenever an inconsistency is detected, it will be solved by the site RC and entered into the database by the PM at the SDC. The PM will audit inclusion, recruitment, and medical record abstraction processes by comparing inclusion / eligibility forms, and data abstracted from charts against medical records on a random sample of 10% of all screened subjects.

At the end of the study, and after a comprehensive quality check and assurance, the final data validation will be run. If there are no inconsistencies, a pre-lock checklist will be used and completion of all data management activities will be confirmed. Once the approval for locking is obtained from all key project stakeholders, including the study statistician, the database will be locked and clean data will be extracted for statistical analysis. The database will not be changed in any manner after locking.

4.3. Statistical methods

4.3.1. Overview

All statistical analysis will be conducted on an intention-to-treat basis, i.e. all randomized participants with baseline and at least one post-baseline measurement will be analyzed by original treatment assignment, regardless of adherence. Baseline characteristics will be described and compared between groups using summary measures (e.g. means, medians, rates) and tests selected based on variable distribution. Analyses will be adjusted for factors used in the stratified randomization (center, age group and type of cancer). In addition, if, despite efforts to standardize intervention delivery (section 5.1), we identify substantial differences in fidelity across sites, we will evaluate effect modification by site including the site × intervention interaction in the models. The False Discovery Rate criterion will be used to correct for multiple testing in non-planned analyses. Co-I Orellana has been the primary biostatistician for all prior PQ analyses and is best suited to head the PQ Response's analysis, along with her team at Deakin University. The PI and Co-I Dussel will be part of the leadership team. While all Co-Is will be involved in analysis and manuscript writing, some may take a more prominent role according to their specific expertise (e.g. Gerhardt, Rosenberg with aim 2, and Bakitas with aim 3). DFCI will coordinate data cleaning and programming.

4.3.2. Analysis Plan

4.3.2.1. Comparison of patient-level outcomes (HRQoL and Symptom distress) - Specific Aim 1

The effect of the intervention on child quality of life and symptom burden outcomes (see section 2.4) will be estimated using mixed linear models, with patient as a random effect and group, time (categorical) and group x time interaction and the stratification factors as fixed effects. . The same analysis will be undertaken for the respective domain subscales of PedsQL (physical and psychosocial) and PQ-MSAS (physical and psychological).

4.3.2.2. Comparison of parent-level outcomes (anxiety, depression, HRQoL, and burden) - Specific Aim 2

The same analytical approach described for patient-level outcomes will be followed to analyze parent S-TAI-State, CES-D-10 mean scores and aRSQ symptom-related scores. In S-TAI-State analysis, we will additionally adjust by baseline S-TAI-Trait score. We will also compare depression (CES-D-10 ≥10) over time between the two groups using generalized linear mixed models with logit link and binomial distribution. As gender is a predictor of these outcomes,[76] it will be included in these models if imbalanced between arms.

4.3.2.3. Comparison of family activation outcomes - Specific Aim 3

Mixed models, similar to those described for patient-level outcomes, will be fitted to estimate the effect of the intervention on change between week 16 and baseline in Brief COPE active coping, planning, and instrumental support Scores, Total No. of, and No. of different, complementary therapies, and No. of psychosocial clinician encounters over the 16-w study period. Link and distribution for these models will be defined based on the distribution of the outcome variable. We will explore whether change in parent's activation score is modified by parent/child characteristics (e.g. parent's gender, S-TAI-Trait, CES-D-10 scores; and child's diagnosis, among others) including the corresponding interaction terms.

4.3.2.4. Additional analyses

We will also conduct a thorough *process evaluation* that includes results from: (a) intervention fidelity indicators (section 2.3.2 and 5.1); and (b) exit interviews. Quantitative data will be reported using descriptive techniques; Qualitative data will be transcribed verbatim and personal identifiers removed. Transcripts will be analyzed with a thematic analysis[77] approach using MAXQDA software. Coding will be inductive and will start with open-coding followed by further iterations to fit the data using the constant comparison method, until theoretical saturation is reached. Differences will be solved by reflective discussion within the study team. Once consensus is reached, a coding dictionary will be developed and the data recoded independently by two researchers. There will be two analysis levels: 1. individual and 2. cross-case analysis (i.e. grouped by age and site). In individual analysis we will triangulate patient and parent outcome and qualitative data with provider views to better understand whether and how processes were influenced (or influenced) the intervention. We will use cross case analyses to explore similarities and differences between cases and compare processes to explain differences in outcomes in relevant subgroups.

Finally, we will also conduct several *exploratory analyses* including: (a) Mediation analysis: We propose that the intervention effect on patient's HRQoL is mediated by parental activation (PA) (Figure 5). If we find a positive intervention effect on patient's HRQoL we will explore PA mediation (measured at 8 and 16 weeks) using the counterfactual framework.[78] The PA mediated (indirect) effect will be computed through G-estimation[79,80] incorporating confounders of the mediator-outcome association, e.g. time since diagnosis or parent S-TAI-Trait

score. A similar approach will be used to explore whether PA mediates intervention's effect on parent HRQoL. These exploratory analyses, albeit limited because of the small sample size, could shed light on PQ response's mechanisms and help further refine the intervention; (b) Age as an effect modifier: we will evaluate whether the effect of the intervention on mean PedsQL and PQ-MSAS total scores over 16-w is modified by age. (c) Subgroup analysis: if sample size permits, we will assess the effect of PQ-Response on symptom burden as reported by children 7-12 years old using the same analytical approach described for patient level outcomes; (d) Parent-patient reports relationship: we will explore correlation (Spearman), convergence and divergence between parent and patient (whenever available) HRQoL and symptom burden reports for total, subscale, and specific item scores.

4.3.3. Considerations regarding missing data

Effective data collection at scheduled times will be monitored and intermittent attrition will be minimized using the strategies described in section 2.3.2. PediQUEST web is designed to avoid involuntary non-completion of items, and incomplete surveys have been less than 1% in prior studies. For the purposes of scoring PedsQL and MSAS we will proceed as suggested by the authors of the scales by imputing the mean to the missing items if more than 50% of the scales have been completed. Exit interview information will inform type of missing data/drop-out process and missing data handling approach. For outcomes where missing completely at random is a plausible assumption, we will do complete case analysis. If missing at random (MR) is highly likely, we will use multiple imputation or inverse probability weighting, depending on the statistical model being considered. For not MR data we will use sensitivity analyses. In all cases we will assess robustness of the estimates to missing data assumptions.

5. Methods: Monitoring

5.1. Data Monitoring

5.1.1. Formal committee

This is a small multisite clinical trial of a supportive care intervention with a risk profile that is comparable to usual cancer care. As such, data monitoring will be primarily carried out by the Study Data Center at DFCI, and a small Data and Safety Monitoring Board (DSMB). A 5-member DSMB composed of five co-investigators representing different disciplines and expertise will be established (see table 4). Three members do not participate in the study (MB, MC, CG) and will act as Independent Monitors. The committee will be convened at the beginning of the study via conference calls, to provide input and

Table 4. Data and Safety Monitoring Board (DSMB)

Member	Title/Discipline	Expertise				Role
		PCR	CTR	BR	QR	
**Liliana Orellana**	PhD, Biostatistics	X	X			Study Lead Statistician
**Marie Bakitas**	DNsC, Nursing	X	X		X	Independent Monitor
**Mary Cooley**	PhD, Nursing	X	X			Independent Monitor
**Cynthia Gerhardt**	PhD, Psychology	X		X	X	Independent Monitor
**Veronica Dussel**	MD, MPH, Medicine	X	X		X	Co-investigator

References: *PCR*: Palliative care Research; *CTR*: Clinical trials research; *BR*: Behavioral research; *QR*: Qualitative research

guidance on study evaluation, including quality assurance and safety issues, as well as data management activities and interim analysis stopping rules.

The DSMB will be in charge of monitoring: 1) data quality for completeness, timeliness, and accuracy including conformance with informed consent requirements; 2) participants' accrual and retention; 3) adverse events; 4) participants' withdrawals, compliance issues or any complaints about the research; 5) intervention fidelity indicators; 6) compliance with data management procedures; 7) reviewing any protocol modifications/deviations that may occur between DSMB review periods; and, 8) reviewing interim analysis and making recommendations if appropriate. All data reviewed by the DSMB will be blinded (except for interim analysis results as explained in 5.1.3). DSMB virtual meetings will take place at least quarterly by videoconference calls. DSMB members will provide quarterly input and feedback to the Steering Committee, via e-mail and conference calls.

5.1.2. Reports

During the 36-month data collection period, the SDC will generate a number of reports to monitor data quality and trial progress. Specifically, the SDC will produce:

- Weekly data monitoring reports informing about the recruitment process (number of participants screened, identified as eligible, and enrolled),
- Monthly reports assessing data quality (completeness, missing and inconsistent data), follow-up (participants' response rate, number of dropouts and reasons), and process measures (to determine whether the intervention is being delivered as expected, e.g. number of participants who did not have the initial meeting with the Response team within three weeks of randomization and other intervention fidelity indicators (see section 2.3.2).
- An annual report summarizing recruitment progress, data quality, protocol violation, and adverse events will be produced to be sent to the local IRBs.

Reports will present figures for the entire study and stratified by site.

5.1.3. Interim Analysis

A blinded primary end-point interim analysis will be conducted by LO at months 12 and 24 after the study opens. The DSMB will determine a priori stopping rules for efficacy and/or occurrence of serious events (the latter is described under Safety Monitoring, section 5.2.3). For efficacy endpoint, if the stopping rule is reached the DSMB will be presented with unblinded results (i.e. they will be able to ascertain which results correspond to the intervention arm). Based on these results, the DSMB will present recommendations to the Steering Committee regarding early stopping or continuation of the trial. Given the minimal risk involved, the modest benefit anticipated, and characteristics of the intervention, which make it unlikely for there to be a concurrent breakthrough result from another study, no changes in the course of this study are expected.

5.2. Potential Risks and Benefits

As a result of participating in the study, we anticipate that enrolled subjects (patient and parent) or participating providers will be exposed to the following risks and benefits:

5.2.1. Potential Risks for Enrolled Subjects (Patients and/or Parents)

Burden from answering study surveys: Expected to be minimal. PQ weekly surveys take on average 10 minutes to be completed and parental surveys between 15 and 30 minutes (for monthly and baseline surveys respectively). Parents can choose to answer monthly parent surveys online or using another method of their preference (phone or in-person). PQ Surveys have been administered already over 1200 times. Only three minor adverse events have been reported (during our pilot) related to the malfunction of the web system which resulted in parents receiving repeated emails. The system was immediately brought down until the glitch was repaired. None of the episodes resulted in a subject withdrawing from the study, and were reported as acceptable burden and understood as part of what the pilot entailed. Also, these malfunctions lead to a permanent safeguard build in the system that blocks the delivery of multiple emails. No further events were reported after this safeguard was implemented. Participants may also be slightly inconvenienced by the exit interviews. We will schedule them at their convenience.

There is a minor risk of emotional reactions such as sadness, anxiety, depression, and fear as a result of completing any surveys or interviews. Notably, no significant psychological adverse events were reported during the pilot. Should a patient or parent become upset as a result of completing online study instruments, face-to-face or phone interviews, they may stop answering. If at any time, the RC finds that a parent or patient appears upset by study procedures, they will notify the local PI who will contact the participant within 24 hours to follow-up. If deemed necessary, the local PI may suggest to the parent or patient that s/he contact their psychosocial provider or, if they have not been in contact with one, the PI can arrange a consultation with someone from the Pediatric Psychosocial Oncology Program.

Risk resulting from randomization: As a result of randomization, subjects may not receive the most efficacious treatment. For example, not being exposed to the Response intervention (control arm) may result in patients having persistent distress, however given that these patients receive usual care, it is expected that if persistent distress is identified it will be addressed by primary oncology teams as usual. On the other hand, being exposed to the Response intervention may result in side effects related to medications or complementary therapies recommended for symptom management, that may not have otherwise been prescribed; however, all participating PC teams include Hospice and Palliative Medicine board certified physicians who have extensive experience with symptom management. Furthermore, Response Team recommendations will be funneled through the primary oncology team. *None of the risks are above and beyond what a patient receiving cancer care might experience anyway*.

Potential for Confidentiality breach: Because of the safeguards in place, we believe that subjects' confidentiality will not be affected and the risk of serious breaches is extremely low. See Section 6.3 for a description of the data protection procedures in place.

5.2.2. Potential Risks for Health Care Providers

An additional potential burden of this study is the time commitment of staff providers. Primary oncology teams will be contacted to identify patients, and some will participate in an in-depth interview (about 20 minutes long). For those having patients in the intervention arm, the integration with the Response team may slightly increase the time dedicated to the patient because of reading, answering emails, or needing to contact or monitor a patient in distress. This burden is expected to be similar to what is typical for when a patient participates in a cancer-directed therapy clinical trial. Also, given that we will typically conduct less than one interview per provider and the relatively small numbers of subjects at each site at any given time (maximum of 5), it is unlikely the burden will fall on any single provider. On the other hand, palliative care (Response) team providers will increase their workload somewhat. They will consult on subjects who may not have been consulted otherwise, which implies time for the visit, writing notes and other coordination activities that may result from the consult. Palliative care teams rotate their on-call providers frequently, so this extra burden will be distributed across providers. Further, as early integration is a goal that most palliative care teams aspire to, the study is seen as an acceptable burden as it may help teams understand staffing needs, should PQ Response result in a positive effect. We will make every effort to conduct provider interviews at times that are convenient for them and minimize staff burden in general.

5.2.3. Safety Monitoring

Safety monitoring will be the responsibility of the SDC and the DSMB. Information on all potential types of adverse events will be collected at all assessment points and recorded on standard forms. In preparation for their quarterly feedback, the DSMB will analyze the rates of adverse events and evaluate the possibility that the intervention is causing harm. If at any point the stopping rules for adverse events are reached (such as a death, suicide or serious consideration of suicide, or a change in mental health requiring hospitalization, that is felt to be related to a recommendation of the Response team), we will suspend study activities and try to determine if there is any link between study procedures and the adverse event and determine if any modification is advised or if the study should be stopped. If an adverse event is detected, the PI will be responsible for promptly reporting it consistent with Dana-Farber Harvard Cancer Center, local sites, and funding agency policies.

5.2.4. Potential Benefits

Although the study is conducted under conditions of equipoise, participants in both study arms will complete weekly PQ Surveys, which based on our original study, may help some patients and parents increase awareness about and discuss distress with their provider. In our prior study, about 40% of children and parents found that filing out the surveys (regardless of receiving feedback) helped them talk to their providers at least sometimes. Subjects randomized to the intervention, may further benefit from receiving feedback (along with their providers) and by being followed by the Response teams. Parents may also experience improved psychological wellbeing as a result of having a more active role regarding their child’s care and possibly due to improvement in the child’s HRQoL.

5.3. Auditing

A schedule for the monitoring plan is presented in Table 5. Compliance with the monitoring plan will be ensured through the SDC's staff close supervision. Electronic data captured through REDCap and PediQUEST web will be subject to electronic validation and will also be visually cross-validated by our staff on a daily basis for complex errors and completeness. SDC staff will also regularly validate electronic versions of source documents. Regular site monitoring will include biweekly calls between the PM and sites' RCs to oversee recruitment and protocol adherence (recurrence of these meeting will be flexible adjusted to study needs). Site RCs will gather required information on an ongoing basis and send reports to the PM. Reports will be reviewed by the PM and the DSMB (over email) and by the Steering Committee during their quarterly meetings. Whenever data quality or trial progress problems, or protocol violations are detected, the local PI will be contacted and, if the problem is severe or persistent, a site visit will be scheduled. The funding agency and intervening IRBs will be notified in compliance with each IRB's regulations.

In addition, SDC staff will conduct a minimum of an annual visit to each site during their enrollment period (more visits will be scheduled if there is concern about protocol adherence). During visits, study staff will review individual participant records, including supporting data, to ensure protection of study participants, compliance with the protocol, and accuracy and completeness of data forms. Site visits will also include review of regulatory files to ensure that regulatory requirements are being followed. The Local PI and RC will make study documents, e.g., consent forms and pertinent clinical records readily available for inspection.

Table 5. Data Monitoring Tasks and Schedule

Responsible	***Task***	***Minimum frequency***
**Local Data Centers (local PI and RC)**	Fill in recruitment report data	Weekly
	Collect data to fill in data quality, follow-up, and process data	Monthly
**Study Data Center (PM and VD)**	Conference call with sites' RCs	Bi-weekly
	Site visits	Annual
	Generation of recruitment reports	Weekly
	Generation of data quality, follow-up, and process reports	Monthly
**DSMB**	Email review of reports	Monthly
	Conference call to analyze data and make recommendations to SC	Quarterly
	Conference call to analyze interim results	Annual
**Steering Committee**	Analyze trial progress and make necessary decisions	Quarterly
**IRB**	Approval of continuing review	Annual

6. Ethics and Dissemination

6.1. Research Ethics/IRB Approval

Protocol, template informed consent forms, participant's materials, and any other requested documents as well as all subsequent modifications, will be reviewed and approved by the sponsor and the applicable Institutional Review Boards with respect to scientific content and compliance with applicable research and human subjects' regulations.

6.2. Consent and Assent

6.2.1. Sites Agreement to Participate

All participating sites have agreed in advance to participate in the study.

6.2.2. Informed Consent at the Participant Level

For all eligible patients, the recruitment protocol will be similar to that used in our prior prospective study and pilot. Recruitment processes were described in section 2.7. As in our prior studies, we ask site IRBs to provide a waiver of individual authorization for disclosure of personal health information to identify eligible subjects.

Patient assent and parental written permission will be secured by the RC before formally entering the study. In both intervention and control arms, participants will provide consent to collect data and to be treated by the Response teams in the event that they are randomized to the intervention arm. Study brochures and informed permission/assent document will contain all the required elements of informed consent including the purpose of the study and procedures, and potential risks and benefits of participation. In all cases, informed consent procedures will be administered by study personnel. If needed, interpreters will be used to aid with the consent process for Spanish speaking families. The forms will be available in English and Spanish. At least one parent will be asked to review and sign the informed permission/ assent documents covering the patient's participation. With regard to minors, for children between 5 and 9 years of age the study brochure will be considered the assent document and verbal assent will be sought whenever possible and if developmentally appropriate. Children and adolescents between 10 and 17 years of age will be invited to review and sign the assent document (there are two assent documents, one for children 10-12 years old and one for teenagers and young adults). Patients who are 18 years or older will be invited to review and provide written consent covering their participation (the assent/permission document is prepared to be signed by an adult patient-participant). A separate consent document will cover caregiver's participation. As described in Section 2.7, verbal consent/assent may also be secured in place of written documentation of consent/assent.

The informed consent document is only a small component of the informed consent process. Adequate time will be provided for describing the study and fielding questions from the patient and/or parents. Fair balance will be maintained while describing the risks and benefits of participation in the study, and no undue pressure will be placed on the patient to enroll in the trial. It will further be explained that lack of participation will not affect the usual and anticipated standard of care. Participants will be encouraged to seek clarification regarding the intervention, and efforts will be made to ascertain whether they understand the information.

6.2.3. Informed Consent at the Health provider level

All health providers working in the palliative care teams and who are eligible to be Response team members will be invited to receive the training. During their participation in training we will inform providers that they may choose not to participate in delivering the intervention, and that declining participation will not affect their job in any way. As for oncology providers, they will be informed through study staff about the purpose and procedures of the study using forums such as meetings, posters, or internal newsletters. With regards to the subset of palliative care and oncology providers who are invited to participate in the exit interview, the elements

of informed consent will be provided on the invitation email (see Appendix-Recruitment Materials), including a statement about how information will be handled and stored.

We request a waiver of documentation of consent for PC and oncology providers based on the following considerations: (i) participating in the study, by either delivering the intervention and/or participating in an interview to reflect on their experience with study patients and procedures, constitutes “no more than minimal risk”; (ii) the rights and welfare of the subjects will not be adversely affected: providers wil be presented with all elements of informed consent, in training, through study staff, and through invitation emails (in the case of exit interviews); (iii) increased study feasiblity: we would like to reduce the burden of participating for providers. Further, in the case of provider interviews, confidentiality is unlikely to be breached given that tapes and transcripts will be coded, securely stored, and de-identified as explained in section 6.3.

6.3. Confidentiality

To carry out the study it will be necessary to collect and store some personal health information (PHI) including:

- Contact information (names, addresses, email addresses, and phone numbers) necessary to identify subjects initially and ask for consent, send the mail surveys throughout the study, and conduct the post-death surveys when applicable. Medical record numbers needed to verify eligibility and abstract medical information.
- Patient’s dates of birth and death, to calculate their age and time since death if appropriate.
- Parent’s date of birth, to calculate their age.

These data will be collected during the recruitment process and through REDCap and PediQUEST web. During recruitment, PHI data will be located in the *pool list, REDCap, and PediQUEST.* The *pool list* will be an excel file stored in RC’s computers or each site’s network and accessible only to the study team. The pool list is programmed to generate a de-identified report that RCs will share weekly with the SDC. Consent documents will be stored in locked cabinets and accessible only to study team members. All research staff collecting PHI will have HIPAA Certification and the training mandated by the Institutional Review Board.

PHI collected through REDCap and PediQUEST web is protected by robust security features including secure user authentication, password encryption in both front and back ends, and role-based access controls that prevents users from accessing data that they are not authorized to see (e.g. patients cannot see other patients’ data and local RCs, Project Manager, or PIs cannot see other sites information; only authorized technical personnel who provide support can have access to each of the system’s back end data). These GCP and HIPAA compliant systems validate the identity of trusted partners using digital certificates and keep a full audit trail of all transactions. Access to PQ-reports for parents, children, and providers outside DFCI’s firewall occurs through a send secure system that requires a one-time registration if the system is accessed via web. If the PQ system is accessed through the App, data travels encrypted from and to the server. REDCap and PediQUEST back end data are stored on secured servers specifically allocated to the study with access limited to authorized research and technical personnel only and with authorization from DFCI’s IS personnel. Secure web access (HTTPS) protocols are used to encrypt data in transit to/from remote electronic devices and the server. The REDCap study database was developed and will be supported by VD (co-I) and

her "REDCap team." The PediQUEST web system was developed in collaboration with DFCI's Clinical Informatics Team and Atrium Technologies, which continues to provide support for the system. All operations are conducted under the auspices of confidentiality agreements developed by DFCI's legal office.

Participants' names will not be directly linked in any way with audio files and interview transcripts will have all personal references removed and will only be identified by study ID. All non-PediQUEST electronic files will be stored as encrypted files on a password protected web-based repository, accessible only to the LDC and SDC. Access to all data will be limited to study personnel on a "need to know" basis.

In addition to these confidentiality safeguards, all study desktops and hand-held computers will be within the sites firewall. All participating centers are licensed cancer/health centers whose information technology groups adhere to policies and practices under the HIPAA regulations, therefore, creating a very tight computing environment, which makes it difficult for individuals external to the study to access the databases.

Identifying information will be stored until the study is closed and then destroyed. In the case of subjects deemed ineligible for the study, all PHI obtained for screening purposes will be destroyed when accrual is met. For the purposes of analysis, DFCI will share with the statistical team at Deakin University a limited dataset containing the following PHI information: patient's dates of birth and death, treatment dates, parent's date of birth, diagnosis, and zip code. These variables are needed to create de-identified variables to be used in the analysis. The only identifying information that will remain in the closed research databases are the child's vital status at 6 months, and date of death in the case of patients who were randomized and died, diagnosis, and zip code, as these will be used in the analysis. De-identified files will be kept indefinitely. We have extensive experience with protecting patients' confidentiality using these methods.

Because of the safeguards in place, we believe that subjects' confidentiality will not be affected, and the risk of serious breaches is extremely low. No study information will be released to any other party except to DFCI or site IRBs and local regulatory authorities, if requested.

6.4. Declaration of Interests

None of the participating investigators declares a conflict of interest concerning this study.

6.5. Access to Data

The SDC will oversee the intra-study data sharing process, with input from the DSMB. While on study, local PIs will have access to their site data and to summary reports of trial progress. Once the database is consolidated, all investigators that are interested in leading a particular analysis, will be given access to the final password-protected de-identified data sets. Out of study investigators may be allowed access to the datasets after a formal analysis proposal is approved by the steering committee. Our statistical team (Co-I Orellana and team at Deakin University) developed the analysis plan and will receive the limited dataset described above for analysis.

6.6. Ancillary and post-trial care

As mentioned in section 2.3.1, all participants in the intervention arm will be offered continued follow-up by the PC team once the intervention ceased to ensure continuity and the same level of care. No access to the PediQUEST system will be provided after the study period. No other specific ancillary care needs are expected.

6.7. Dissemination policy

Analysis and reports of the PediQUEST Response trial results will include the full sample, unless scientifically justified. The Steering Committee will make recommendations regarding when and what material should be submitted for publication. Each paper will be reviewed and approved by the SC members prior to submission. The SC will work to reduce the interval between end of data collection and release of the study results. We expect to take about 4 to 6 months to compile and submit the main paper. Study results will be released to the participating physicians and referring physicians through publications in peer-reviewed journals, congress abstracts, and oral presentations, whereas specific materials will be produced to disseminate results among study participants and the general public.

6.8. Anticipated Problems and Solutions

Anticipated potential problems could include <u>failure to meet sample target</u>, <u>high attrition</u>, <u>contamination</u> in usual care arm, <u>feasibility</u>, and <u>social desirability bias</u>. Given the strategies presented earlier, we expect strong recruitment rates. From prior experience attrition due to death and drop-out are expected to be low and were accounted for in power calculations. However, we will collect socio-demographic data and reasons for exit which will help understand both recruitment and attrition drivers. As we successfully did in prior studies,[38] PI and Co-Is will closely monitor recruitment and retention rates and characteristics. If concern arises, we will revise strategies together and problem solve. As discussed earlier, contamination risk is expected to be very low. Even so, we will monitor the proportion of clinicians who provided care to trial participants in both arms and evaluate behavior shifts during exit interviews, to understand whether contamination was an issue. We believe that the PQ Response intervention is highly feasible: (a) It builds on our prior successful research work; (b) all participating sites have long-standing working relationships and very similar oncology care and PC team approaches; and, (c) intervention has been carefully piloted and suggests feasibility. Social desirability bias is a common issue in un-blinded studies. We plan to minimize this risk by not emphasizing the specific outcomes we are targeting, and by careful training and supervision of RCs.

7. Study Timeline

The proposed study will require 5 years to complete and will include three phases: 9-months of study implementation activities (trial set-up), 36-months intervention and data collection (start times staggered by site), and 18-months of data analyses and manuscripts preparation and dissemination. The Table below presents the timeline and key activities of the proposed research.

	YR1				YR2				YR3				YR4				YR5			
Activities	Q1	Q2	Q3	Q4	Q1	Q2	Q3	Q4	Q1	Q2	Q3	Q4	Q1	Q2	Q3	Q4	Q1	Q2	Q3	Q4
**Study Implementation Activities**																				
Hiring of personnel																				
IRB Protocol submission (at sites)																				
Design of training and study materials, PediQUEST Database																				
Adaptation + deployment of PediQUEST web t sites, debugging																				
Training of Pall care teams + RC																				
**Conduct RESPONSE trial**																				
Site 1																				
Site 2																				
Site 3																				
Site 4																				
**Monitoring and analysis**																				
Monitoring of intervention fidelity				X	X	X	X	X	X	X	X	X	X	X	X	X				
Interim data analysis (design, program and run)																				
Full data analysis (design, program and run)																				
Manuscript submission																				
New grant preparation																				

8. Importance of the Knowledge to be Gained

The potential risks for study subjects may be considered comparable to those of receiving usual cancer care and counterbalanced by the knowledge gained and the prospect of direct benefit. The information gained may elucidate strategies for early integration of palliative care in children with advanced cancer and for enhancing quality of life for children and their families. As such, the risk/benefit balance for this study appears favorable. Further, this knowledge may be extended to children with other life-threatening conditions. These outcomes have been designated as high priorities by the National Institutes of Health.

9. References

1. Chan A-W, Tetzlaff JM, Altman DG, Laupacis A, Gøtzsche PC, Krle A-Jerić K, Hrobjartsson A, Mann H, Dickersin K, Berlin JA, Dore CJ, Parulekar WR, Summerskill WSM, Groves T, Schulz KF, Sox HC, Rockhold FW, Rennie D, Moher D. SPIRIT 2013 Statement: defining standard protocol items for clinical trials. Rev Panam Salud Publica Pan Am J Public Health. 2015 Dec;38(6):506–514. PMID: 27440100
2. Temel JS, Greer JA, Muzikansky A, Gallagher ER, Admane S, Jackson VA, Dahlin CM, Blinderman CD, Jacobsen J, Pirl WF, Billings JA, Lynch TJ. Early palliative care for patients with metastatic non-small-cell lung cancer. N Engl J Med. 2010 Aug 19;363(8):733–742. PMID: 20818875
3. Bakitas M, Lyons KD, Hegel MT, Balan S, Brokaw FC, Seville J, Hull JG, Li Z, Tosteson TD, Byock IR, Ahles TA. Effects of a palliative care intervention on clinical outcomes in patients with advanced cancer: the Project ENABLE II randomized controlled trial. JAMA. 2009 Aug 19;302(7):741–749. PMCID: PMC3657724
4. Basch E, Deal AM, Kris MG, Scher HI, Hudis CA, Sabbatini P, Rogak L, Bennett AV, Dueck AC, Atkinson TM, Chou JF, Dulko D, Sit L, Barz A, Novotny P, Fruscione M, Sloan JA, Schrag D. Symptom Monitoring With Patient-Reported Outcomes During Routine Cancer Treatment: A Randomized Controlled Trial. J Clin Oncol Off J Am Soc Clin Oncol. 2015 Dec 7; PMID: 26644527
5. Dionne-Odom JN, Azuero A, Lyons KD, Hull JG, Tosteson T, Li Z, Li Z, Frost J, Dragnev KH, Akyar I, Hegel MT, Bakitas MA. Benefits of Early Versus Delayed Palliative Care to Informal Family Caregivers of Patients With Advanced Cancer:

Outcomes From the ENABLE III Randomized Controlled Trial. J Clin Oncol Off J Am Soc Clin Oncol. 2015 May 1;33(13):1446–1452. PMCID: PMC4404423

6. Razzak M. Palliative care: ASCO provisional clinical opinion. Nat Rev Clin Oncol. 2012 Apr;9(4):189. PMID: 22371134
7. World Health Organization. Strengthening of palliative care as a component of integrated treatment throughout the life course [Internet]. 2013 Dec. Report No.: EB134/28. Available from: http://apps.who.int/gb/ebwha/pdf_files/EB134/B134_28-en.pdf
8. American Academy of Pediatrics. Committee on Bioethics and Committee on Hospital Care. Palliative care for children. Pediatrics. 2000;106(2 Pt 1):351–7.
9. Nass S. J., Patlak M., National Cancer Policy Forum, Board on Health Care Services, Institute of Medicine, The National Academies of Sciences E and Medicine; Comprehensive Cancer Care for Children and Their Families: Summary of a Joint Workshop by the Institute of Medicine and the American Cancer Society [Internet]. Washington, DC: National Academy of Sciences; 2015. Available from: http://www.nap.edu/download.php?record_id=21754
10. Lyon ME, Garvie PA, Briggs L, He J, Malow R, D'Angelo LJ, McCarter R. Is it safe? Talking to teens with HIV/AIDS about death and dying: a 3-month evaluation of Family Centered Advance Care (FACE) planning - anxiety, depression, quality of life. HIVAIDS Auckl NZ. 2010;2:27–37. PMCID: PMC3218704
11. Lyon ME, Jacobs S, Briggs L, Cheng YI, Wang J. A longitudinal, randomized, controlled trial of advance care planning for teens with cancer: anxiety, depression, quality of life, advance directives, spirituality. J Adolesc Health Off Publ Soc Adolesc Med. 2014 Jun;54(6):710–717. PMID: 24411819
12. Hays RM, Valentine J, Haynes G, Geyer JR, Villareale N, Mckinstry B, Varni JW, Churchill SS. The Seattle pediatric palliative care project: Effects on family satisfaction and health-related quality of life. J Palliat Med. 2006;9(3):716–28.
13. Wolfe J, Hammel JF, Edwards KE, Duncan J, Comeau M, Breyer J, Aldridge SA, Grier HE, Berde C, Dussel V, Weeks JC. Easing of suffering in children with cancer at the end of life: is care changing? J Clin Oncol Off J Am Soc Clin Oncol. 2008 Apr 1;26(10):1717–1723. PMID: 18375901
14. Zhukovsky DS, Herzog CE, Kaur G, Palmer JL, Bruera E. The impact of palliative care consultation on symptom assessment, communication needs, and palliative interventions in pediatric patients with cancer. J Palliat Med. 2009 Apr;12(4):343–349. PMID: 19327071
15. Vollenbroich R, Duroux A, Grasser M, Brandstätter M, Borasio GD, Führer M. Effectiveness of a pediatric palliative home care team as experienced by parents and health care professionals. J Palliat Med. 2012 Mar;15(3):294–300. PMID: 22216782
16. Kassam A, Skiadaresis J, Alexander S, Wolfe J. Differences in end-of-life communication for children with advanced cancer who were referred to a palliative care team. Pediatr Blood Cancer. 2015 Aug;62(8):1409–1413. PMID: 25882665
17. Friedrichsdorf SJ, Postier A, Dreyfus J, Osenga K, Sencer S, Wolfe J. Improved quality of life at end of life related to home-based palliative care in children with cancer. J Palliat Med. 2015 Feb;18(2):143–150. PMID: 25401507
18. Wolfe J, Grier HE, Klar N, Levin SB, Ellenbogen JM, Salem-Schatz S, Emanuel EJ, Weeks JC. Symptoms and suffering at the end of life in children with cancer. N Engl J Med. 2000 Feb 3;342(5):326–333. PMID: 10655532
19. Wolfe J, Orellana L, Ullrich C, Cook EF, Kang TI, Rosenberg A, Geyer R, Feudtner C, Dussel V. Symptoms and Distress in Children With Advanced Cancer: Prospective Patient-Reported Outcomes From the PediQUEST Study. J Clin Oncol Off J Am Soc Clin Oncol. 2015 Jun 10;33(17):1928–1935. PMCID: PMC4451175
20. Schrag NM, McKeown RE, Jackson KL, Cuffe SP, Neuberg RW. Stress-related mental disorders in childhood cancer survivors. Pediatr Blood Cancer. 2008;50(1):98–103.
21. Zeltzer LK, Recklitis C, Buchbinder D, Zebrack B, Casillas J, Tsao JCI, Lu Q, Krull K. Psychological status in childhood cancer survivors: a report from the Childhood Cancer Survivor Study. J Clin Oncol Off J Am Soc Clin Oncol. 2009 May 10;27(14):2396–2404. PMCID: PMC2677925
22. Kreicbergs U, Valdimarsdóttir U, Onelöv E, Björk O, Steineck G, Henter J-I. Care-related distress: a nationwide study of parents who lost their child to cancer. J Clin Oncol Off J Am Soc Clin Oncol. 2005 Dec 20;23(36):9162–9171. PMID: 16172455
23. Rosenberg AR, Baker KS, Syrjala K, Wolfe J. Systematic review of psychosocial morbidities among bereaved parents of children with cancer. Pediatr Blood Cancer. 2012 Apr;58(4):503–512. PMCID: PMC3270147
24. Heath JA, Clarke NE, Donath SM, McCarthy M, Anderson VA, Wolfe J. Symptoms and suffering at the end of life in children with cancer: an Australian perspective. Med J Aust. 2010 Jan 18;192(2):71–75. PMID: 20078405

25. Hechler T, Blankenburg M, Friedrichsdorf SJ, Garske D, Hübner B, Menke A, Wamsler C, Wolfe J, Zernikow B. Parents' perspective on symptoms, quality of life, characteristics of death and end-of-life decisions for children dying from cancer. Klin Pädiatr. 2008 Jun;220(3):166–174. PMID: 18478489
26. Johnston DL, Nagel K, Friedman DL, Meza JL, Hurwitz CA, Friebert S. Availability and use of palliative care and end-of-life services for pediatric oncology patients. J Clin Oncol Off J Am Soc Clin Oncol. 2008 Oct 1;26(28):4646–4650. PMID: 18824711
27. Feudtner C, Womer J, Augustin R, Remke S, Wolfe J, Friebert S, Weissman D. Pediatric palliative care programs in children's hospitals: a cross-sectional national survey. Pediatrics. 2013 Dec;132(6):1063–1070. PMID: 24190689
28. Johnston DL, Vadeboncoeur C. Palliative care consultation in pediatric oncology. Support Care Cancer Off J Multinatl Assoc Support Care Cancer. 2012 Apr;20(4):799–803. PMID: 21479523
29. Brock KE, Steineck A, Twist CJ. Trends in End-of-Life Care in Pediatric Hematology, Oncology, and Stem Cell Transplant Patients. Pediatr Blood Cancer. 2015 Oct 29; PMID: 26513237
30. Wentlandt K, Krzyzanowska MK, Swami N, Rodin G, Le LW, Sung L, Zimmermann C. Referral practices of pediatric oncologists to specialized palliative care. Support Care Cancer Off J Multinatl Assoc Support Care Cancer. 2014 Sep;22(9):2315–2322. PMID: 24671435
31. Wolfe J. Suffering in children at the end of life: recognizing an ethical duty to palliate. J Clin Ethics. 2000 Summer;11(2):157–63.
32. Wolfe J, Klar N, Grier HE, Duncan J, Salem-Schatz S, Emanuel EJ, Weeks JC. Understanding of prognosis among parents of children who died of cancer: impact on treatment goals and integration of palliative care. JAMA. 2000 Nov 15;284(19):2469–2475. PMID: 11074776
33. Dussel V, Kreicbergs U, Hilden JM, Watterson J, Moore C, Turner BG, Weeks JC, Wolfe J. Looking beyond where children die: determinants and effects of planning a child's location of death. J Pain Symptom Manage. 2009 Jan;37(1):33–43. PMCID: PMC2638984
34. Mack JW, Joffe S, Hilden JM, Watterson J, Moore C, Weeks JC, Wolfe J. Parents' views of cancer-directed therapy for children with no realistic chance for cure. J Clin Oncol Off J Am Soc Clin Oncol. 2008 Oct 10;26(29):4759–4764. PMCID: PMC2653133
35. Dussel V, Joffe S, Hilden JM, Watterson-Schaeffer J, Weeks JC, Wolfe J. Considerations about hastening death among parents of children who die of cancer. Arch Pediatr Adolesc Med. 2010 Mar;164(3):231–237. PMID: 20194255
36. Ullrich CK, Dussel V, Hilden JM, Sheaffer JW, Lehmann L, Wolfe J. End-of-life experience of children undergoing stem cell transplantation for malignancy: parent and provider perspectives and patterns of care. Blood. 2010 May 13;115(19):3879–3885. PMID: 20228275
37. Wolfe J, Orellana L, Cook EF, Ullrich C, Kang T, Geyer JR, Feudtner C, Weeks JC, Dussel V. Improving the care of children with advanced cancer by using an electronic patient-reported feedback intervention: results from the PediQUEST randomized controlled trial. J Clin Oncol Off J Am Soc Clin Oncol. 2014 Apr 10;32(11):1119–1126. PMCID: PMC3970170
38. Dussel V, Orellana L, Soto N, Chen K, Ullrich C, Kang TI, Geyer JR, Feudtner C, Wolfe J. Feasibility of Conducting a Palliative Care Randomized Controlled Trial in Children With Advanced Cancer: Assessment of the PediQUEST Study. J Pain Symptom Manage. 2015 Jun;49(6):1059–1069. PMCID: PMC4530789
39. Rosenberg AR, Orellana L, Ullrich C, Kang T, Geyer JR, Feudtner C, Dussel V, Wolfe J. Quality of Life in Children with Advanced Cancer: A Report from the PediQUEST Study. J Pain Symptom Manage. 2016 May 21; PMID: 27220948
40. Craig P, Dieppe P, Macintyre S, Michie S, Nazareth I, Petticrew M, Medical Research Council Guidance. Developing and evaluating complex interventions: the new Medical Research Council guidance. BMJ. 2008;337:a1655. PMCID: PMC2769032
41. Wilson IB, Cleary PD. Linking clinical variables with health-related quality of life. A conceptual model of patient outcomes. JAMA. 1995 Jan 4;273(1):59–65. PMID: 7996652
42. Ferrans CE, Zerwic JJ, Wilbur JE, Larson JL. Conceptual model of health-related quality of life. J Nurs Scholarsh Off Publ Sigma Theta Tau Int Honor Soc Nurs Sigma Theta Tau. 2005;37(4):336–342. PMID: 16396406
43. Basch E, Abernethy AP. Supporting clinical practice decisions with real-time patient-reported outcomes. J Clin Oncol. 2011 Mar 10;29(8):954–6.
44. Hibbard JH, Mahoney ER, Stock R, Tusler M. Do increases in patient activation result in improved self-management behaviors? Health Serv Res. 2007 Aug;42(4):1443–1463. PMCID: PMC1955271

45. Shigaki C, Kruse RL, Mehr D, Sheldon KM, Bin Ge null, Moore C, Lemaster J. Motivation and diabetes self-management. Chronic Illn. 2010 Sep;6(3):202–214. PMID: 20675362
46. Donald M, Ware RS, Ozolins IZ, Begum N, Crowther R, Bain C. The role of patient activation in frequent attendance at primary care: a population-based study of people with chronic disease. Patient Educ Couns. 2011 May;83(2):217–221. PMID: 20598825
47. Warren JS, Brown CR, Layne CM, Nelson PL. Parenting self-efficacy as a predictor of child psychotherapy outcomes in usual care: a multi-dimensional approach. Psychother Res J Soc Psychother Res. 2011 Jan;21(1):112–123. PMID: 21331978
48. Bollinger LM, Nire KG, Rhodes MM, Chisolm DJ, O'Brien SH. Caregivers' perspectives on barriers to transcranial Doppler screening in children with sickle-cell disease. Pediatr Blood Cancer. 2011 Jan;56(1):99–102. PMID: 20842753
49. Hommel KA, Denson LA, Baldassano RN. Oral medication adherence and disease severity in pediatric inflammatory bowel disease. Eur J Gastroenterol Hepatol. 2011 Mar;23(3):250–254. PMCID: PMC3073772
50. Nicholson O, Mellins C, Dolezal C, Brackis-Cott E, Abrams EJ. HIV treatment-related knowledge and self-efficacy among caregivers of HIV-infected children. Patient Educ Couns. 2006 Jun;61(3):405–410. PMID: 16246515
51. Varni JW, Burwinkle TM, Katz ER, Meeske K, Dickinson P. The PedsQL in pediatric cancer: reliability and validity of the Pediatric Quality of Life Inventory Generic Core Scales, Multidimensional Fatigue Scale, and Cancer Module. Cancer. 2002 Apr 1;94(7):2090–2106. PMID: 11932914
52. Varni JW, Burwinkle TM, Seid M, Skarr D. The PedsQL 4.0 as a pediatric population health measure: feasibility, reliability, and validity. Ambul Pediatr Off J Ambul Pediatr Assoc. 2003 Dec;3(6):329–341. PMID: 14616041
53. Collins JJ, Byrnes ME, Dunkel IJ, Lapin J, Nadel T, Thaler HT, Polyak T, Rapkin B, Portenoy RK. The measurement of symptoms in children with cancer. J Pain Symptom Manage. 2000 May;19(5):363–377. PMID: 10869877
54. Collins JJ, Devine TD, Dick GS, Johnson EA, Kilham HA, Pinkerton CR, Stevens MM, Thaler HT, Portenoy RK. The measurement of symptoms in young children with cancer: the validation of the Memorial Symptom Assessment Scale in children aged 7-12. J Pain Symptom Manage. 2002 Jan;23(1):10–16. PMID: 11779663
55. Spielberger CD. Manual for the State-Trait Anxiety Inventory. Palo Alto: Consulting Psychologists Press Inc.; 1983.
56. Radloff LS. The CES-D Scale: A Self-Report Depression Scale for Research in the General Population. Appl Psychol Meas. 1977 Jun 1;1(3):385–401.
57. Compas B. Responses to Stress Questionnaire (RSQ) [Internet]. Stress and Coping Research Lab. Vanderbilt University. [cited 2017 Nov 27]. Available from: http://vkc.mc.vanderbilt.edu/stressandcoping/rsq/
58. Carver CS. You want to measure coping but your protocol's too long: consider the brief COPE. Int J Behav Med. 1997;4(1):92–100. PMID: 16250744
59. Thorpe KE, Zwarenstein M, Oxman AD, Treweek S, Furberg CD, Altman DG, Tunis S, Bergel E, Harvey I, Magid DJ, Chalkidou K. A pragmatic-explanatory continuum indicator summary (PRECIS): a tool to help trial designers. J Clin Epidemiol. 2009 May;62(5):464–475. PMID: 19348971
60. Upton P, Lawford J, Eiser C. Parent-child agreement across child health-related quality of life instruments: a review of the literature. Qual Life Res Int J Qual Life Asp Treat Care Rehabil. 2008 Aug;17(6):895–913. PMID: 18521721
61. Eiser C, Varni JW. Health-related quality of life and symptom reporting: similarities and differences between children and their parents. Eur J Pediatr. 2013 Oct;172(10):1299–1304. PMID: 23715654
62. Varni JW, Limbers CA. The pediatric quality of life inventory: measuring pediatric health-related quality of life from the perspective of children and their parents. Pediatr Clin North Am. 2009 Aug;56(4):843–863. PMID: 19660631
63. Kraemer HC, Measelle JR, Ablow JC, Essex MJ, Boyce WT, Kupfer DJ. A new approach to integrating data from multiple informants in psychiatric assessment and research: mixing and matching contexts and perspectives. Am J Psychiatry. 2003 Sep;160(9):1566–1577. PMID: 12944328
64. De Los Reyes A, Thomas SA, Goodman KL, Kundey SMA. Principles underlying the use of multiple informants' reports. Annu Rev Clin Psychol. 2013;9:123–149. PMCID: PMC4103654
65. Kaurin A, Egloff B, Stringaris A, Wessa M. Only complementary voices tell the truth: a reevaluation of validity in multi-informant approaches of child and adolescent clinical assessments. J Neural Transm Vienna Austria 1996. 2016 Aug;123(8):981–990. PMID: 27118025
66. Kaye EC, Friebert S, Baker JN. Early Integration of Palliative Care for Children with High-Risk Cancer and Their Families. Pediatr Blood Cancer. 2015 Nov 18; PMID: 26579997

67. EPEC Pediatrics : Education in Palliative and End-of-Life Care: Feinberg School of Medicine: Northwestern University [Internet]. [cited 2017 Jan 19]. Available from: http://bioethics.northwestern.edu/programs/epec/curricula/pediatrics.html
68. Bellg AJ, Borrelli B, Resnick B, Hecht J, Minicucci DS, Ory M, Ogedegbe G, Orwig D, Ernst D, Czajkowski S, Treatment Fidelity Workgroup of the NIH Behavior Change Consortium. Enhancing treatment fidelity in health behavior change studies: best practices and recommendations from the NIH Behavior Change Consortium. Health Psychol Off J Div Health Psychol Am Psychol Assoc. 2004 Sep;23(5):443–451. PMID: 15367063
69. Varni JW, Katz ER, Seid M, Quiggins DJ, Friedman-Bender A, Castro CM. The Pediatric Cancer Quality of Life Inventory (PCQL). I. Instrument development, descriptive statistics, and cross-informant variance. J Behav Med. 1998 Apr;21(2):179–204. PMID: 9591169
70. Bona K, London WB, Guo D, Frank DA, Wolfe J. Trajectory of Material Hardship and Income Poverty in Families of Children Undergoing Chemotherapy: A Prospective Cohort Study. Pediatr Blood Cancer. 2016 Jan;63(1):105–111. PMID: 26398865
71. Sherbourne CD, Stewart AL. The MOS social support survey. Soc Sci Med 1982. 1991;32(6):705–714. PMID: 2035047
72. Compas BE, Boyer MC, Stanger C, Colletti RB, Thomsen AH, Dufton LM, Cole DA. Latent variable analysis of coping, anxiety/depression, and somatic symptoms in adolescents with chronic pain. J Consult Clin Psychol. 2006;74(6):1132–1142.
73. Drake R, Frost J, Collins JJ. The symptoms of dying children. J Pain Symptom Manage. 2003 Jul;26(1):594–603. PMID: 12850642
74. Barrera M, D'Agostino NM, Gibson J, Gilbert T, Weksberg R, Malkin D. Predictors and mediators of psychological adjustment in mothers of children newly diagnosed with cancer. Psychooncology. 2004 Sep;13(9):630–641. PMID: 15334531
75. Hagan TL, Fishbein JN, Nipp RD, Jacobs JM, Traeger L, Irwin KE, Pirl WF, Greer JA, Park ER, Jackson VA, Temel JS. Coping in Patients With Incurable Lung and Gastrointestinal Cancers: A Validation Study of the Brief COPE. J Pain Symptom Manage. 2017 Jan;53(1):131–138.
76. Kearney JA, Salley CG, Muriel AC. Standards of Psychosocial Care for Parents of Children With Cancer. Pediatr Blood Cancer. 2015 Dec;62 Suppl 5:S632-683. PMID: 26700921
77. Boyatzis RE. Transforming qualitative information: thematic analysis and code development [Internet]. Thousand Oaks, CA: Sage Publications; 1998. Available from: http://www.loc.gov/catdir/enhancements/fy0656/97045405-d.html
78. Robins, J. M., Richardson, T. S., Ornstein K. Alternative graphical causal models and the identification of direct effects. In: Shrout PE, editor. Causality Psychopathol Find Determinants Disord Their Cures Am Psychopathol Assoc [Internet]. Oxford; New York: Oxford University Press; 2011 [cited 2016 Jan 28]. Available from: http://public.eblib.com/choice/publicfullrecord.aspx?p=665423
79. Vansteelandt S. Estimating direct effects in cohort and case-control studies. Epidemiol Camb Mass. 2009 Nov;20(6):851–860. PMID: 19806060
80. Valeri L, Vanderweele TJ. Mediation analysis allowing for exposure-mediator interactions and causal interpretation: theoretical assumptions and implementation with SAS and SPSS macros. Psychol Methods. 2013 Jun;18(2):137–150. PMCID: PMC3659198
