# Supplementary material for: A multisite randomized controlled trial of an early palliative care intervention in children with advanced cancer: The PediQUEST Response Study Protocol
Source: PLoS One. 2022 Nov 8;17(11):e0277212. doi: 10.1371/journal.pone.0277212 (PMC9642881; doi:10.1371/journal.pone.0277212)

PediQUEST Response
Randomized Controlled Trial

Consent Documents Compiled

Research Consent Form

for Social and Behavioral Research

Dana-Farber/ Harvard Cancer Center

BIDMC/BCH/BWH /DFCI/MGH/Partners Network Affiliates

OHRS 09.23.16a

Protocol Title: The PediQUEST Response Intervention Study

DF/HCC Principal Research Investigator / Institution: Joanne Wolfe, DFCI

DF/HCC Site-Responsible Research Investigator(s) / Institution(s): Christina Ullrich, DFCI/BCH

PARENT WRITTEN CONSENT FORM

INTRODUCTION AND KEY INFORMATION

Research is a way of gaining new knowledge. All research is voluntary. It is your choice whether you take part in this research or not. If you decide to participate, please sign and date at the end of this form. We will give you a copy and you can refer to this consent form at any time.

The following is a short summary of this research study to help you decide whether you would like to participate. More detailed information is provided later in this form.

For the purposes of this research, you will be referred to as a "participant" and we will use the word child to refer to your son or daughter.

1. **Why am I being invited to take part in a research study?**

We are inviting you to take part in a research study called "The PediQUEST Response Intervention Study." You were selected as a potential participant because your child is under a treatment for a type of cancer or tumor that may cause symptoms and discomfort.

2. **Why is this research being done?**

DFCI Protocol Number:	TBD	Approved Date (DFCI IRB Approval):	TBD
Date Posted for Use:	TBD	Expiration Date (Invalid for use on or after):	TBD
OnCore Version Date:	TBD	Content Last Revised with OHRS Review:	TBD

We know from previous studies that children, teens, and young adults with cancer or tumors can experience physical and emotional symptoms that bother them, even though there are available treatments for these symptoms that may help.

For this reason, we developed the PediQUEST Response intervention, which is a new way of providing care that intends to improve quality of life for young patients with cancer or tumors and their parents, like you. In this study we want to learn whether PediQUEST Response helps children, teens, and young adults feel better than those receiving usual care.

3. Who is supporting this research?

The National Institute of Nursing Research (NINR) from the National Institutes of Health is providing the funding for this study.

4. What does this research study involve and how long will it last?

This research study involves completing surveys about how you and your child are doing over a period of approximately 18 weeks. We will also collect some information from your child's medical records. At the end of the study, the research team will ask you to participate in an interview to share your thoughts about the study.
If you are assigned to receive the PediQUEST Response intervention you will also receive a summary of your and your child's answers to the surveys and will meet with a team of doctors, nurse practitioners, and social workers who specialize in symptom management and strategies to optimize quality of life. Here, at DF/HCC, this team is called the *Pediatric Advanced Care Team (PACT)*.

It is expected that about 400 people (200 children, teens, and young adults, and 200 parents) from five different research sites will take part in this study.

5. What are the risks to participating in this study?

There are risks to taking part in any research study. We want to make sure you know about a few key risks right now. More detailed information is provided in the "What are the risks or discomforts of the research study?" section.

Risks to participating in this research study may include:
- Your child may potentially not receive the treatment that is best for their quality of life. This is because we do not know whether the PediQUEST Response intervention is better than usual care, which is why we are doing the study.
- Potentially becoming upset by completing the questionnaires or meeting with the PediQUEST Response Team

6. Will this study benefit me in any way?

Taking part in this research study may or may not benefit you and/or your child. We hope the information learned from this research study will provide sound evidence about how to best care for children with cancer or tumors and their families.

7. What are my options?

Taking part in this research study is voluntary. Instead of being in this research study, you can decide not to participate in this research study and that your child continues to receive usual care

Your child's oncologist is aware that we are inviting you and your child to participate in this study. However, you will decide whether to participate. If you choose not to participate, are not eligible to participate, or withdraw from this research study, this will not affect your child's present or future care and will not cause any penalty or loss of benefits to which you are otherwise entitled.

In the next sections of this research consent form we will explain more about why this research study is being done, what is PediQUEST Response, what is involved in participating in this study, possible risks and benefits of participation, alternatives to participation, and your rights as a research participant.

We encourage you to take some time to think this over and to discuss it with other people and to ask questions now and at any time in the future.

A. WHY IS THIS RESEARCH STUDY BEING DONE?

As we mentioned above, we know that children, teens, and young adults with cancer or tumors experience physical and emotional symptoms that can bother them, even though there are available treatments for these symptoms that may help.

We developed a software called PediQUEST (which stands for Pediatric Quality of Life and Evaluation of Symptoms Technology). PediQUEST asks questions about a child/teen/young adult's symptoms and quality of life. The system is also able to create a report summarizing the answers.

In the PediQUEST Response Study, we are testing a system of care that includes:

1) Receiving *PediQUEST Reports* sent to families and providers each time you and/or your child answer a PediQUEST survey and,

2) Meeting regularly with the *PediQUEST Response Team*, an interdisciplinary team made up of doctors, nurse practitioners, and social workers who specialize in symptom management and strategies to optimize quality of life. At each institution, the teams working as the "PediQUEST Response Team" may be called differently. Here, at DF/HCC, it is called the *Pediatric Advanced Care Team (PACT)*. The Response team will also receive PediQUEST Reports and will be in close communication with your child's primary oncology team so that they can work together to help your child feel better when needed.

We have evaluated PediQUEST Response in a small number of patients and are now at the point of doing a randomized trial to find out whether PediQUEST Response improves quality of life in a larger group of children, teens, and young adults.

In this trial, we will compare PediQUEST Response to the usual current care provided where your child receives treatment.

B. WHAT OTHER OPTIONS ARE THERE?

Taking part in this research study is voluntary. Instead of being in this research study, you have the following options:

- Decide not to participate in this research study and that your child continues to receive usual care

C. WHAT IS INVOLVED IN THE RESEARCH STUDY?

If you and your child agree to participate, both of you will be asked to fill out online PediQUEST surveys every week for a total of 18 weeks (see below for more on these Surveys). We will ask you to answer some other questionnaires as well. Over the next pages we will explain in detail what will happen during the study, starting with a **figure in the next page that summarizes study activities.**

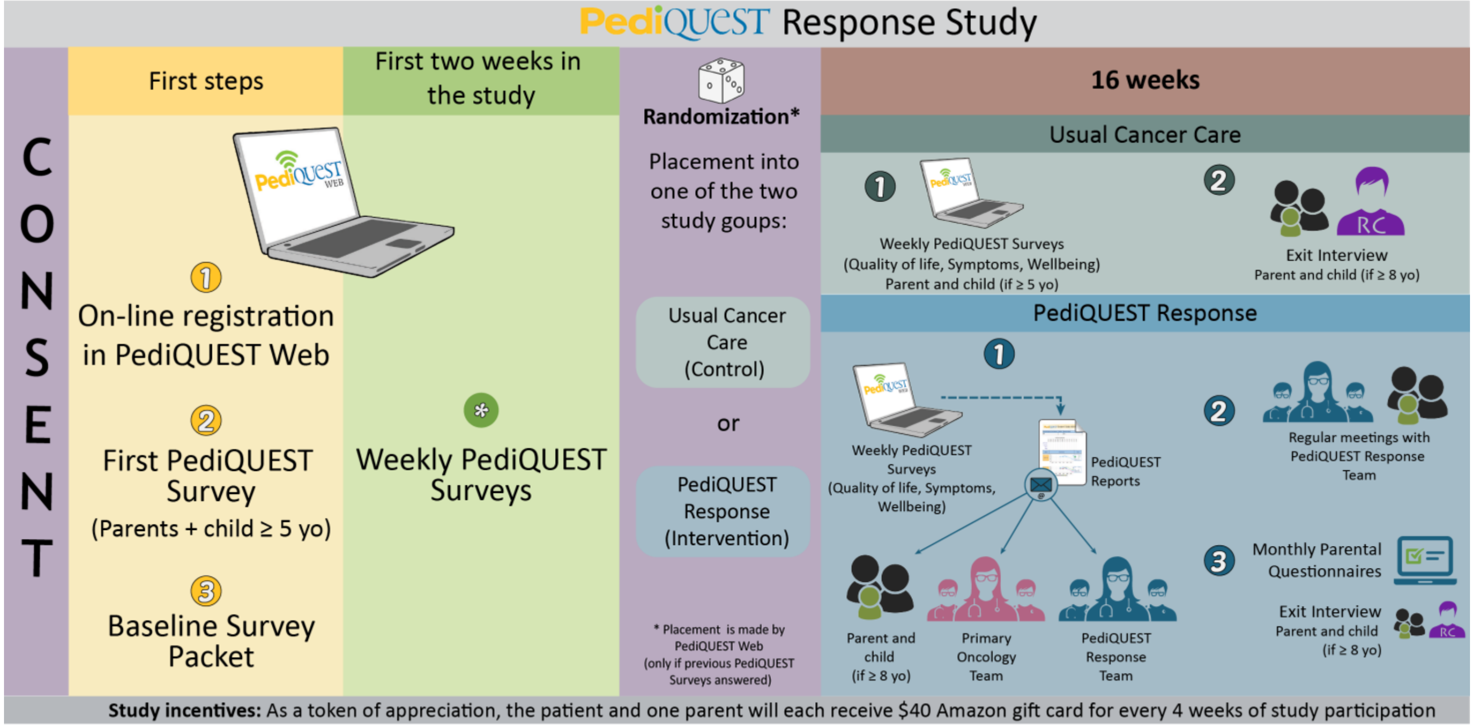

DFCI Protocol Number: TBD	Approved Date (DFCI IRB Approval): TBD
Date Posted for Use: TBD	Expiration Date (Invalid for use on or after): TBD
OnCore Version Date: TBD	Content Last Revised with OHRS Review: TBD

Right after signing the consent:

- The research coordinator will work with you to complete ***on-line registration in PediQUEST web***: You will be registered as a PediQUEST user. If your child is 8 years old or older, s/he will have a separate PediQUEST account. An email address is required to create each PediQUEST web account. We can assist you in setting up a new email account if needed. You can access the PediQUEST system through a computer or mobile App (for tablet or cell phones). A research coordinator can help you to set-up the app on your device if needed.
- You and your child (depending on age) will be assigned the first ***PediQUEST survey***. The research coordinator will walk you through the survey and help you become familiar with it. More details about this survey are explained below.
- You will also be assigned the ***Baseline Parent Packet***: this 15-minute online questionnaire contains questions about you and your child (for example, age, race, education, how you are feeling, your supports, and how you feel about certain aspects of your child's care).

First two weeks in the study:

- During the first two weeks, we ask that you answer ***weekly PediQUEST Surveys***.

PediQUEST Surveys ask how your child has been feeling, for example if pain, nausea, or other symptoms are bothersome. They also ask about how your child is feeling about other things in life, such as school and friends. PediQUEST Surveys can be answered on any electronic device from anywhere that you can connect to the internet. Whenever a new survey is available for you to answer, PediQUEST web will send an email with a link to the survey or a notification if you are using the App.

Who will answer a PediQUEST Survey?

- Regardless of your child's age, you will receive an email or notification in *your account* asking you to answer a PediQUEST survey every week. A PediQUEST Survey usually takes about 10-15 minutes.

- If your child is *between 5 and 7 years old*, in addition to your survey, there will also be some questions for *your child and we will ask you to read the questions out loud to your child*. Your child's part should take between 5 to 10 minutes.
- If your child is *8 years old or older*, your child will receive a separate PediQUEST Survey to answer through your child's account.

You can answer all the questions at once or you can pause the survey if you need a break or stop filling out the survey at any point. None of the answers to a PediQUEST Survey will directly become part of your child's medical record.

We expect you to answer each survey within 2 days of receiving it. If you don't answer, an email reminder or notification will be sent to you daily. If after 2 days you have not answered, that survey will no longer be available and a new survey will become available the next week.

Placement into one of the study groups (randomization)

- If you or your child have answered at least two surveys during the first two weeks of the study, you and your child will be placed into one of two groups, the PediQUEST Response (intervention) or the usual care (control) group. The group assignment is randomly made by the PediQUEST web system (you cannot choose which group you and your child will be in and nor can we). If you have not answered at least two surveys, you will not continue to be in the study.
- At this time, the first *Monthly Parent Questionnaire* will be assigned. See below for more information on these surveys.

From randomization up to end of study (16 weeks)

- If you are in the ***intervention group***:
  - You will continue to receive and fill out *weekly PediQUEST Surveys* and will start receiving *PediQUEST Reports (through email or the App)*. If your child is older than 8 years of age, each of you will receive the reports through your own accounts. PediQUEST Reports summarize your and/or your child's answers

(depending on who answered the survey) in a graphical manner. If you wish to open the report from your computer, you will need to set up an account with the *send secure system*, a system that protects your privacy (this is done only once and we can show you how to do this).

The primary oncology team (doctor, nurse practitioner, nurse and psychosocial clinician) and the PediQUEST Response Team will also receive the report via email or App. No one other than clinicians and research staff will have direct access to these reports.

- You and your child will also meet with the *PediQUEST Response Team* as soon as this can be scheduled. The research coordinator will let the Response Team know that you have been assigned to the intervention. Somebody from that team will contact you to *schedule your first meeting with the Response Team*. After this first meeting, it is recommended that you continue to meet monthly until the end of the study. Follow-up meetings, however, may be different for different patients and will depend on what you, your child, and the Response Team arrange. During these meetings, the PediQUEST Response Team will focus on understanding more about how your child is doing and what, if anything, might be bothering them, so that they can make recommendations to help him/her feel as well as possible.

  As with any other medical consultation, the PediQUEST Response Team will be in close communication with your child's primary oncology team and together will figure out what may be done to help your child feel better. If s/he is already feeling better by the time you meet with the PediQUEST Response team, they may be able to provide recommendations to prevent symptoms from coming back as strongly. They may also recommend that your child sees another specialist.

  If one of the reports shows that your child is in distress, or if the clinical team made some recommendations to ease your child's distress, a PediQUEST Response Team member may call to check in about how s/he is feeling. All interactions with the Response team will be documented in your child's medical

record. Also, they may communicate with the oncology team face-to-face, by phone or by email.

- If you are in the ***control group***:
  - Your child will continue to receive *care as usual*
  - You and your child will continue to receive and fill out *weekly PediQUEST surveys*, but neither you nor your child, or his/her providers will receive reports. You will *not* have contact with the PediQUEST Response team as part of the study.
- ***Regardless of the arm*** of the study in which you are in,
  - You will fill out *Monthly Parent Questionnaires* every four weeks, beginning right after randomization. The PediQUEST web system will send an email or notification indicating surveys are available to answer. These surveys take between 15 and 25 minutes and ask about how you have been feeling, about some treatments that your child may have received during the past month, and how you feel regarding certain aspects of your child's care.
  - Members of the research staff will regularly review your child's medical chart to collect information about his/her illness and treatments during the study period.

End of Study (Week 18)

- At the end of the study, we will invite you to participate in an exit interview, which will be audio-recorded, so that we can learn more about your views on participating in the study.

Token of Appreciation

- As a token of appreciation for your participation in the study, we will provide both you and your child with a $20 gift card at the time of enrollment, covering the first

two weeks of participation and, from there on, each of you will receive $40 gift cards (e.g. Amazon) every four weeks you remain in the study

D. HOW LONG WILL I BE IN THIS RESEARCH STUDY?

You will be in this research study for about eighteen weeks.

You may be taken off the research study for reasons such as:

- It is considered in your or your child's best interest
- The study procedures are found to be unsafe or ineffective
- There is any problem with following study procedures (for example, if you do not answer PediQUEST Surveys during the first two weeks in the study)
- Your child has a stem cell transplant while enrolled in the study
- There are any problems with research funding
- Or for any other reason

If you are removed from the research study, an Investigator will explain to you why you were removed.

In addition, you can stop participating in the research study at any time (see section G).

E. WHAT ARE THE RISKS OR DISCOMFORTS OF THE RESEARCH STUDY?

There are risks to taking part in any research study. There are no anticipated physical risks from participating. You may become upset by completing the questionnaires or meeting with the PediQUEST Response Team. If this happens, you can stop answering one or more surveys and/or meeting with the Response team. There are several support services available to discuss your feelings. You can talk with your child's physician, nurse, or psychosocial clinician. You can also reach the head of the study, Dr. Joanne Wolfe (617-632-5286) who can help arrange support from your clinician in the Pediatric Psychosocial Oncology Program. If you have not been in contact with one, Dr. Wolfe can arrange a consultation with someone in that program.

Another risk is that, because there are two groups in the study, your child may not receive the most effective treatment. There is no way to know upfront which of the two approaches is more effective, which is why we are doing the study. If during the research study, there is new information that may affect your child's health or your willingness to participate, we will let you know. You may be asked to sign a new consent form that shows that you have been told new information related to this research study.

A third risk is loss of confidentiality. We will use several security measures to protect confidentiality. They are explained in detail in section K.

F. WHAT ARE THE BENEFITS OF THE RESEARCH STUDY?

Taking part in this research study may or may not benefit you and/or your child. We hope the information learned from this research study will provide sound evidence about how to best care for children with cancer or tumors and their families.

G. CAN I STOP BEING IN THE RESEARCH STUDY AND WHAT ARE MY RIGHTS?

You have the right to choose not to sign this form. If you decide not to sign this form, you and your child cannot participate in this research study.

You can stop being in the research study at any time. Tell the research doctor or research coordinator if you are thinking about stopping or decide to stop. Leaving the research study will not affect your child's medical care. You can still get your child´s medical care as you used to.

If you choose to not participate, or if your child is not eligible to participate, or if you withdraw from this research study, this will not affect your child's present or future care and will not cause any penalty or loss of benefits to which you or your child are otherwise entitled.

H. WHAT ARE THE COSTS?

Taking part in this research study may or may not lead to added costs to your or your child's insurance company. Your child's insurance company will be charged for portions of their care during this research study that could be considered usual care, including the PediQUEST Response Team visits. You may be responsible for co-payments and deductibles that are standard for your child's insurance coverage.

You can use your own computer, tablet or phone with internet access to complete PediQUEST Surveys and look at reports. If you are at the hospital or clinic, a member of the study team can provide you with a tablet to use if you prefer. Let them know. If you do not have a computer or tablet at home, the study can provide you with temporary use, while you are enrolled in the study, of a tablet and internet access to complete PediQUEST questionnaires.

If you have questions about your insurance coverage, or the items you might be required to pay for, please call financial services for information. The contact information for financial services:

- Boston Children's Hospital: (617) 355-7188
- Dana-Farber Cancer Institute: (617) 632-3455

The National Cancer Institute provides an online resource to help people participating in cancer clinical trials understand which services their insurance company is required by law to pay. This can be found at the website below.

http://www.cancer.gov or 1-800-4-CANCER (1-800-422-6237)

I. WHAT HAPPENS IF I AM INJURED OR BECOME SICK BECAUSE I TOOK PART IN THIS RESEARCH STUDY?

If you think you or your child have been injured as a result of taking part in this research study, tell the person in charge of this research study as soon as possible. The research doctor's name and phone number are listed in this consent form.

The treating hospital will offer you or your child the care needed to treat injuries directly resulting from taking part in this research. These treatments will be billed to your or your child's insurance company. You will be responsible for deductibles and co-payments. There are no plans to pay you or give you other compensation for the injury. You do not give up your legal rights by signing this form.

J. FUTURE USE OF DATA AND SPECIMENS

Your personal information and/or data collected during this study may be stored and used for future research. Any personal identifiers will be removed, before the data are shared, so that the information cannot be linked back to you.

K. WHAT ABOUT CONFIDENTIALITY?

We will use several measures to protect the privacy and security of all your or your child's personal information, but we cannot guarantee complete confidentiality of study data.

Information created by this study will be securely stored in your child's study file. Paper study documents will be stored in locked cabinets at the Dana-Farber Cancer Institute. Handling of electronic information will be protected by strong security measures (including the use of passwords and encryption of data for transmission) and will be stored on servers specifically allocated to the study which use secure web access (HTTPS) protocols. In the case that we record an interview, audio files will only be identified by a unique study code, no direct links to your or your child's name will be

used; when audio files are transcribed, all personal references will be removed. Study data including your names and other identifying information will not be accessible to anyone outside of the study team (more details about how and with whom we will share information can be found in section N). Some pieces of the study will become part of your child's medical record (for example medical notes written by the PediQUEST Response Team).

The results of this research study may be published. You or your child will not be identified in publications without your permission.

A description of this clinical trial will be available on http//:www.ClinicalTrials.gov, as required by U.S. Law. This web site will not include information that can identify you or your child. At most, the website will include a summary of the results. You can search this website at any time.

L. WHOM DO I CONTACT IF I HAVE QUESTIONS ABOUT THE RESEARCH STUDY?

If you have questions about the study, please contact the research Investigator or study staff as listed below:

Dana-Farber Cancer Institute

- Joanne Wolfe, MD, MPH: 617-632-5286 or page at 617-632-3352. (Principal Investigator)
- Madeline Bilodeau: 617-632-3248 (Study Project Manager)

For questions about your rights as a research participant, please contact a representative of the Office for Human Research Studies at DFCI (617) 632-3029. This can include questions about your participation in the study, concerns about the study, a research related injury, or if you feel/felt under pressure to enroll in this research study or to continue to participate in this research study.

M. CERTIFICATE OF CONFIDENTIALITY (COC)

To help protect your privacy, we have been issued a Confidentiality Certificate from the National Institutes of Health (NIH). With this Certificate, the researchers on this study cannot be forced (for example, by court subpoena) to disclose information that may identify you in federal, state, or local civil, criminal, administrative, legislative or other proceedings.

Disclosure will be necessary upon request of a United States federal or state government agency sponsoring the project that will be used for audit or program evaluation purposes or to meet the requirements of the federal Food and Drug Administration (FDA).

You should understand that a Certificate of Confidentiality does not prevent you or a member of your family or even the research doctor from voluntarily releasing information about yourself or your involvement in this research. If an insurer, medical care provider, or employer learns about your participation, and obtains your consent to receive research information, then we cannot use the Certificate of Confidentiality to withhold this information. This means that you and your family must actively protect your own privacy.

The Certificate of Confidentiality cannot be used to prevent disclosure to state or local authorities when there is a duty to report concerns of abuse, neglect, self-harm or a danger to others.

N. PRIVACY OF PROTECTED HEALTH INFORMATION

Federal law requires Dana-Farber/Harvard Cancer Center (DF/HCC) and its affiliated research doctors, health care providers, and physician network to protect the privacy of information that identifies you or your child and relates to your or your child's past, present, and future physical and mental health conditions ("protected health information"). If you enroll in this research study, your and your child's "protected health information" will be used and shared with others as explained below.

1. What protected health information about me or my child will be used or shared with others during this research?

- Contact information (names, addresses, and phone numbers)
- Your and your child's date of birth.
- Child medical record numbers, existing medical records, including mental health records.
- New health information created from PediQUEST Web questionnaires and PediQUEST Response visits.

2. Why will protected information about me or my child be used or shared with others?

The main reasons include the following:

- To conduct and oversee the research described earlier in this form;
- To ensure the research meets legal, institutional, and accreditation requirements
- Other reasons may include for intervention, payment, or health care operations.

3. Who will use or share protected health information about me or my child?

- Clinicians participating in the intervention and, DF/HCC and its affiliated researchers and entities participating in the research will use and share your protected health information. In addition, other DF/HCC offices that deal with research oversight, billing or quality assurance will be able to use and share your protected health information.

4. With whom outside of DF/HCC may my or my child's protected health information be shared?

While all reasonable efforts will be made to protect the confidentiality of your protected health information, it may also be shared with the following entities:

- Outside individuals or entities that have a need to access this information to perform functions relating to the conduct of this research on behalf of DF/HCC and its affiliates (for example, data storage companies, insurers, or legal advisors).
- The sponsor(s) of this study, and its subcontractors.
- Other research doctors and medical centers participating in this research
- Federal and state agencies (for example, the Department of Health and Human Services, the Food and Drug Administration, the National Institutes of Health, and/or the Office for Human Research Protections), or other domestic or foreign government bodies if required by law and/or necessary for oversight purposes. A qualified representative of the FDA and the National Cancer Institute may review your child's medical records.
- Hospital accrediting agencies.
- A data safety monitoring board organized to oversee this research.

Some who may receive your protected health information may not have to satisfy the privacy rules and requirements. They, in fact, may share your information with others without your permission.

5. For how long will protected health information about me or my child be used or shared with others?

- Because research is an ongoing process, there is no scheduled date at which your protected health information that is being used or shared for this research will be destroyed.

6. Statement of privacy rights:

- You have the right to withdraw your permission for the researchers and participating DF/HCC entities to use or share your or your child protected health

information. We will not be able to withdraw all the information that already has been used or shared with others to carry out related activities such as oversight, or that is needed to ensure quality of the study. To withdraw your permission, you must do so in writing by contacting the researcher listed above in the section: "Whom do I contact if I have questions about the research study?"

- You have the right to request access to your and your child's protected health information that is used or shared during this research and that is related to your or your child's treatment or payment for treatment, but you may access this information only after the study is completed. To request this information, please contact the researcher listed above in the section: "Whom do I contact if I have questions about the research study?"

O. PERMISSION FOR FUTURE CONTACT:

Sometimes, after analyzing the results of a study, additional research questions come up. This is especially frequent in studies like this, where you/your child and other participants share with us a lot of high quality and in-depth information. Should this happen, we may want to contact you/your child later to find out how you are doing or ask questions related to your participation in this study. Your participation in these new research studies is voluntary, and you will not be penalized or lose any benefits if you refuse to participate or decide to stop.
Please indicate whether you want to be contacted if future research questions came up:

☐ Yes Initials_____________ Date___/___/_____

☐ No Initials_____________ Date___/___/_____

P. DOCUMENTATION OF CONSENT

My signature below indicates:

- I have had enough time to read the consent and think about participating in this study;
- I have had all of my questions answered to my satisfaction;
- I am willing to participate in this study;
- I have been told that my participation is voluntary and I can withdraw at any time
- I agree to be audio-recorded if participating in an exit interview.

______________________________ _________________________

Signature of Participant Date

DFCI Protocol Number: TBD	Approved Date (DFCI IRB Approval): TBD
Date Posted for Use: TBD	Expiration Date (Invalid for use on or after): TBD
OnCore Version Date: TBD	Content Last Revised with OHRS Review: TBD

Adult Participants

To be completed by person obtaining consent:

The consent discussion was initiated on ___________________ (date).

Signature of individual obtaining consent: ____

Printed name of above: ____

Date: _______________

☐ A copy of this signed consent form will be given to the participant or legally authorized representative, or, where the participant is a minor, the participant's parent or legal guardian.

For Adult Participants

☐ 1) The participant is an adult and provided consent to participate.

☐ 1a) Participant (or legally authorized representative) is a non-English speaker and signed the translated Short Form in lieu of English consent document:

As someone who understands both English and the language spoken by the participant, I interpreted and/or witnessed, in the participant's language, the researcher's presentation of the English consent form. The participant was given the opportunity to ask questions.

Signature of Interpreter/Witness: ____

Printed Name of Interpreter/Witness: ____

Date: _______________

☐ 1b) Participant is physically unable to sign the consent form because:

☐ The participant is illiterate.

☐ The participant has a physical disability.

☐ Other (please describe): ____

The consent form was read to the participant who was given the opportunity to ask questions and who communicated agreement to participate in the research.

Signature of Witness: ____

Printed Name of Witness: ____

Date: ________________

☐ 2) The participant is an adult who lacks capacity to provide consent and his/her legally authorized representative:

☐ 2a) gave permission for the adult participant to participate

☐ 2b) did not give permission for the adult participant to participate

Dear <name>,

You are receiving this letter because you and your child are eligible for our research study, PediQUEST Response and we would like to invite you to be part of it. We know from previous studies that children, teens, and young adults experience physical and emotional symptoms that can bother them, even though there are available treatments for these symptoms that may help. PediQUEST Response is a new way of providing care that aims to improve quality of life for young patients with cancer and their parents. We want to learn whether children, teens, and young adults receiving PediQUEST Response do in fact feel better than those receiving usual care.

Below you will find additional information about the study. If you do not wish to participate in the study or be further contacted about it, please complete the opt-out document attached and email it back to pediquestweb@dfci.harvard.edu. If we do not receive the opt-out email, a member of our study team will contact you via phone to provide additional details about the study and see if you and your child are willing to participate. Your child's oncologist is aware that we are inviting you and your child to participate in this study.
The decision to participate is yours!

Who is supporting this research?
The National Institute of Nursing Research (NINR) are supporting this research study by providing funding.

What is involved in this research study?
If you and your child agree to participate, you will fill out online PediQUEST surveys (on a computer, tablet or phone) every week for a total of 18 weeks. The graphic below summarizes study activities, and additional information can be found below.

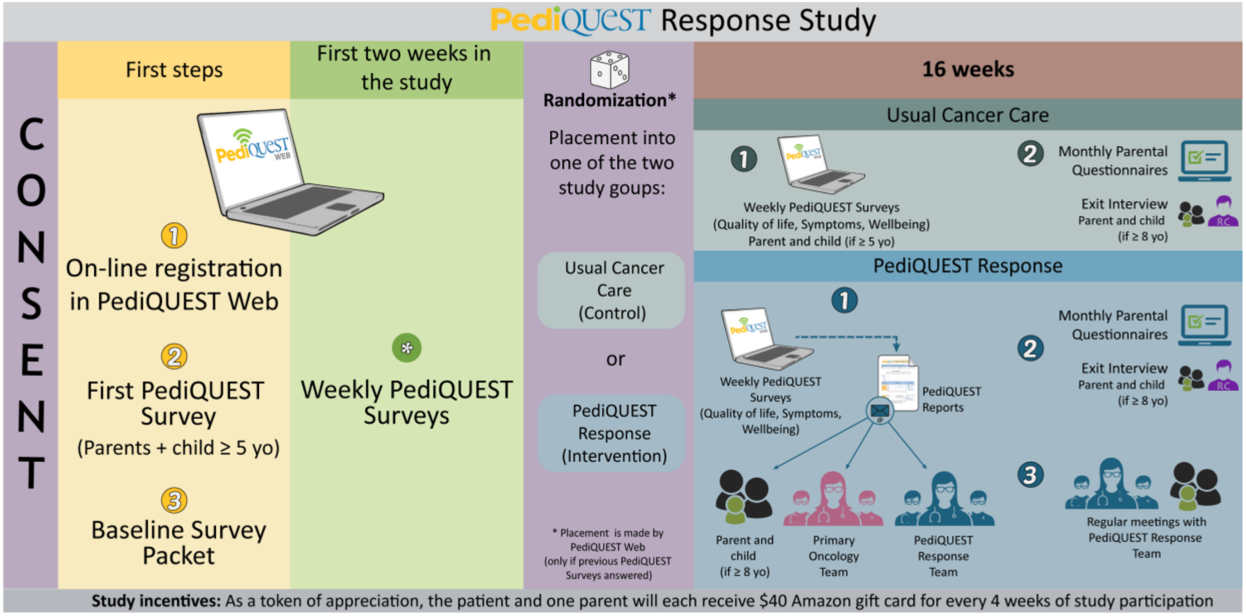

Dana-Farber Cancer Institute | Boston Children's

Dana-Farber/Boston Children's Cancer and Blood Disorders Center

Registration: If you decide to participate, a research coordinator will work with you to complete online registration using PediQUEST web, the system where you will complete weekly surveys.

Completing weekly surveys: Each week, you and your child (if older than 5) will complete weekly surveys through either the PediQUEST website or mobile app.

- These surveys take about 10-15 minutes to complete. They ask about how your child has been feeling and any symptoms s/he may be having. It also asks about other aspects of life such as school or friends.

Parent Surveys: As a parent, you will also receive a baseline survey and five additional monthly surveys throughout the entire study that ask about how you have been feeling and how you feel about certain aspects of your child's care. These surveys take between 15 and 25 minutes to complete.

Placement into one of the study groups (randomization): After two weeks of completing weekly surveys, you and your child will be placed into one of two groups, the PediQUEST Response (intervention) or the usual care (control) group. The group assignment is randomly made by the PediQUEST web system (you cannot choose which group you and your child will be in and nor can we). If you have not answered at least two surveys, you will not continue to be in the study.

- *Intervention group:* If you are in the intervention group, you will continue to receive and fill out weekly PediQUEST Surveys and will start receiving PediQUEST reports via e-mail or the App. Your child's primary oncology team will also receive these reports. Reports summarize your child's and your answers using graphs. You will also meet the Response Team, at the hospital or virtually. Response is an interdisciplinary team of doctors, nurse practitioners, and social workers who specialize in symptom management and strategies to optimize quality of life. They will also receive PediQUEST reports and work with you, your child, and your child's oncology team to help you all feel better when needed.
- *Usual care group:* If you are in the usual care group, you and your child will answer PediQUEST surveys and s/he will continue to receive care as usual, but neither you nor your child, or his/her providers will receive PediQUEST reports. You will not have contact with the Response Team as part of the study.
- *For all participants:* Regardless of which group you are in, members of the research staff will regularly review your child's medical chart to collect information about his/her illness and treatments during the study period.

End of study: At the end of the study, we will invite you to participate in an audio-recorded exit interview so that we can learn more about your experiences in the study.

Token of appreciation: As a token of appreciation, you and your child will each receive a $40 Amazon gift card for each month you remain in the study.

What are the benefits, risks, or discomforts of the research study?

Taking part in this research study may or may not benefit you and/or your child. We hope the information learned from this research study will help us to understand the experience of children, adolescents, and young adults with cancer. It is possible that you or your child may become upset answering the questionnaires or meeting with the Response Team. If this happens, you can skip any questions that you do not wish to answer or skip surveys, and/or stop meeting with the Response team. You can also stop being on the study at any point. Support services are available to you if it would be helpful to discuss any feelings that arise due to your participation in this study. You can also talk with your child's physician, nurse or psychosocial clinician. Another risk is that, because there are two groups in the study, your child may not receive the most effective treatment. There is no way to know upfront which of the two approaches is more effective and this why we are doing the study. If during the research study there is new information that may affect your child's health or your willingness to participate, we will let you know.

Confidentiality and Future Use of Data

We will take measures to protect the privacy and security of all your and your child's personal information, but we cannot guarantee complete confidentiality of study data. Paper study documents will be stored in locked cabinets at the Dana-Farber Cancer Institute. Handling of electronic information will be protected by strong security measures (including the use of passwords and data encryption) and will be stored on secure servers. In the case that we record an interview, audio files will only be identified by a unique study code and no direct links to your or your child's name will be used; when audio files are transcribed, all personal references will be removed. Study data including your names and other identifying information will not be accessible to anyone outside of the study team.

Alternatives

Participation in this study is completely voluntary. If you choose to participate, you may stop participating at any time. Your decision to participate or not participate will not impact the quality or type of medical care you or your family receives at Dana-Farber or Boston Children's Hospital. If you decide to participate in this study, we may want to contact you again for future research.

This project has been approved by the institutional Review Board of Dana-Farber Cancer Institute. If you have further questions, you may contact Madeline Bilodeau, Project Manager, at 617-632-3248, or Joanne Wolfe, Principle Investigator, at 617-632-5286. If you have any questions regarding your rights as a research participant or any research-related questions or concerns, please contact the Office for Human Research Studies at Dana-Farber at 617-632-3029.

Thank you for considering participation in this study. We believe it will provide us with important insight into the experiences of children, adolescents, and young adults so that we can better care for patients like your child in the future.

Research Consent Form

for Social and Behavioral Research

Dana-Farber/ Harvard Cancer Center

BIDMC/BCH/BWH /DFCI/MGH/Partners Network Affiliates

OHRS 09.23.16a

Protocol Title: The PediQUEST Response Intervention Study

DF/HCC Principal Research Investigator / Institution: Joanne Wolfe, DFCI

DF/HCC Site-Responsible Research Investigator(s) / Institution(s): Christina Ullrich, DFCI/BCH

ADOLESCENT WRITTEN PERMISSION/ASSENT AND YOUNG ADULT CONSENT FORM

INTRODUCTION AND KEY INFORMATION

Research is a way of gaining new knowledge. All research is voluntary. It is your choice whether you take part in this research or not. If you decide to participate, please sign and date at the end of this form. We will give you a copy and you can refer to this consent form at any time.

The following is a short summary of this research study to help you decide whether you would like to participate. More detailed information is provided later in this form.

1. **Why am I being invited to take part in a research study?**

We are inviting you to take part in a research study called "The PediQUEST Response Intervention Study." You were selected as a potential participant for this study because you are a teen, or young adult that is under a treatment for a type of cancer or tumor that may cause symptoms and discomfort.

2. **Why is this research being done?**

We know from previous studies that children, teens, and young adults with cancer or tumors can experience physical and emotional symptoms that bother them, even though there are available treatments for these symptoms that may help.

For this reason, we developed the PediQUEST Response intervention, which is a new way of providing care that intends to improve quality of life for patients like you and their parents. In this study, we want to learn whether PediQUEST Response helps children, teens, and young adults feel better than those receiving usual care.

3. **Who is supporting this research?**

The National Institute of Nursing Research (NINR) from the National Institutes of Health provides the funding for this research study.

4. **What does this research study involve and how long will it last?**

You and one of your parents will complete surveys that ask how you are feeling over a period of approximately 18 weeks. We will also collect some information from your medical records. At the end of the study, the research team will ask you to participate in an interview to share your thoughts about the study.
If you are assigned to the group that receives the PediQUEST Response intervention you will also receive a summary of your and your parent's answers to the surveys and will meet with a team of doctors, nurse practitioners, and social workers who specialize in symptom management and strategies to make your quality of life better. Here, at DF/HCC, this team is called the *Pediatric Advanced Care Team (PACT)*.

In the PediQUEST Response Study it is expected that about 400 people (200 children and 200 parents) from five different research sites will take part.
Your oncologist is aware that we are inviting you and your parent to participate in this study. However, you will decide whether or not to participate. If you choose not to participate, are not eligible to participate, or withdraw from this research study, this will not affect your present or future care at the institution.

In the next sections of this research consent form we will explain more about why this research study is being done, what is PediQUEST Response, what is involved in participating in this randomized controlled study, possible risks and benefits of

participation, alternatives to participation, and your rights as a research participant. A "participant" is a person who participates in a research study.

We encourage you to take some time to think this over and to discuss it with other people and to ask questions now and at any time in the future.

A. WHY IS THIS RESEARCH STUDY BEING DONE?

As we mentioned above, we know that children, teens and young adults experience physical and emotional symptoms that can bother them, even though there are available treatments for these symptoms that may help.

We developed a software called PediQUEST (which stands for Pediatric Quality of Life and Evaluation of Symptoms Technology). PediQUEST asks questions about a teen/young adult's symptoms and quality of life. The system is also able to create a report summarizing the answers.

In the PediQUEST Response Study we are testing a system of care which includes:

1) Receiving *PediQUEST Reports*, sent to families and providers each time you and/or your parent answer a PediQUEST Survey.

2) Meeting regularly with the *PediQUEST Response Team*, an interdisciplinary team made up of doctors, nurse practitioners, and social workers who specialize in symptom management and strategies to improve quality of life. At each institution, the teams working as the "PediQUEST Response Team" may be called differently. Here, at DF/HCC, it is called the *Pediatric Advanced Care Team (PACT)*. The Response team will also receive PediQUEST Reports and will be in close communication with your primary oncology team so that they can work together to help you feel better when needed.

We have evaluated PediQUEST Response in a small number of patients and are now at the point of doing a randomized trial to find out whether PediQUEST Response improves quality of life in a larger group of children, teens and young adults.

In this trial we will compare PediQUEST Response to the usual current care provided at the hospital where you receive your treatment.

B. WHAT OTHER OPTIONS ARE THERE?

Taking part in this research study is voluntary. Instead of being in this research study, you have the following options:

- Decide not to participate in this research study and receive usual care

C. WHAT IS INVOLVED IN THE RESEARCH STUDY?

If you and your parent decide to participate, both of you will be asked to fill out online PediQUEST surveys every week for a total of 18 weeks (see below for more on these Surveys). We will ask one of your parents to answer some other questionnaires as well. Over the next pages we will explain in detail what will happen during the study, starting with a **figure in the next page that summarizes study activities.**

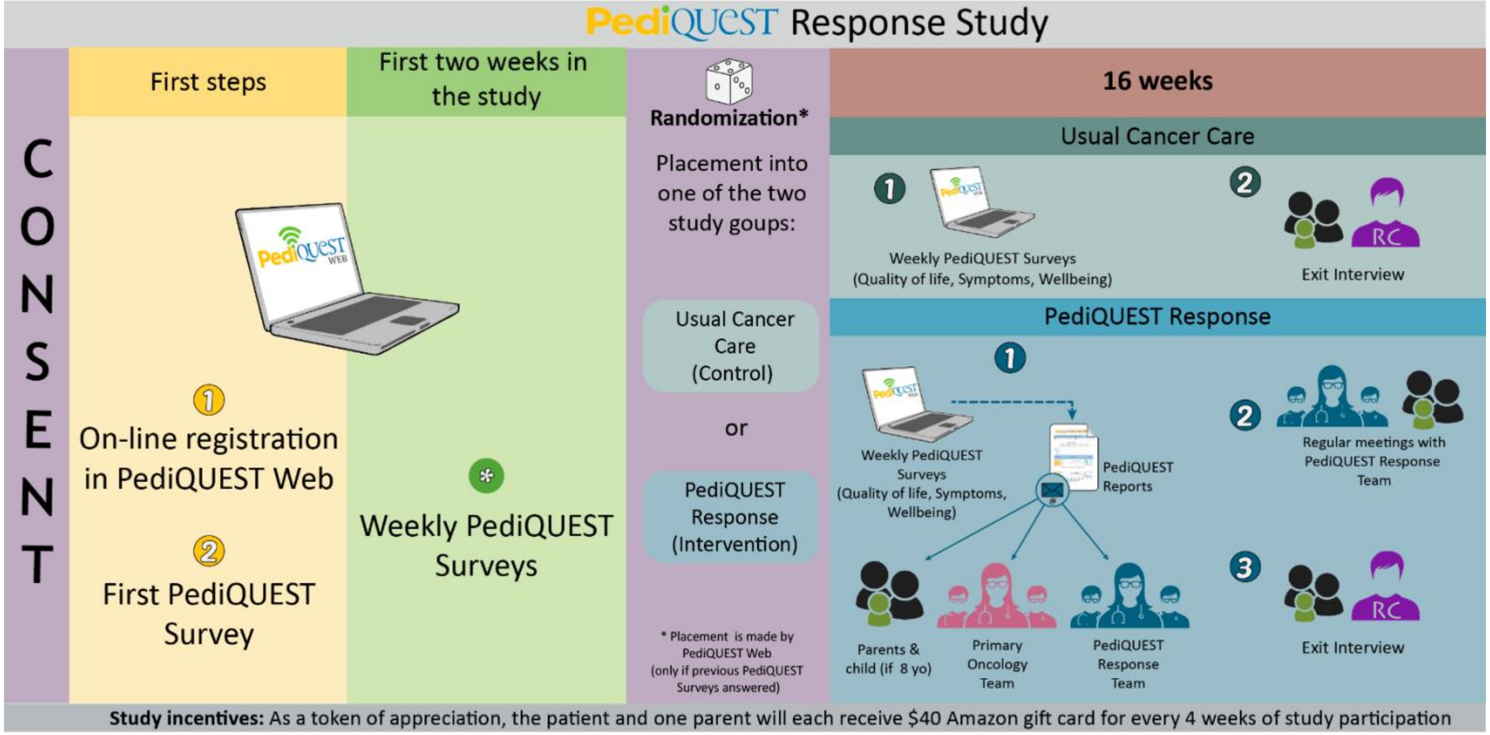

DFCI Protocol Number:	TBD	Approved Date (DFCI IRB Approval):	TBD
Date Posted for Use:	TBD	Expiration Date (Invalid for use on or after):	TBD
OnCore Version Date:	TBD	Content Last Revised with OHRS Review:	TBD

Right after signing the consent:

- The research coordinator will work with you to complete ***<u>on-line registration in PediQUEST web</u>***: you will be registered as a PediQUEST user. An email is required. Your parent will have a separate account. We can assist you or your parent in setting up a new email account if needed. You can access the PediQUEST system through a computer or a mobile App (for tablet or cell phones). A research coordinator can help you to set-up the app on your device if needed.
- You and your parent will be assigned the first ***<u>PediQUEST survey</u>***. The research coordinator will walk you through the survey and help you become familiar with it. More details about this survey are explained below.

First two weeks in the study:

- During the first two weeks, we ask that you answer ***<u>weekly PediQUEST Surveys</u>***.

PediQUEST Surveys ask how you have been feeling, for example if pain, nausea, or other symptoms are bothering you. It also asks about how you are feeling about other things in life, such as school and friends. PediQUEST Surveys can be answered on any electronic device from anywhere that you can connect to the internet. Whenever a new survey is available for you to answer, PediQUEST web will send an email with the link to the survey or a notification if you are using the App.

Who will answer a PediQUEST Survey?

- *<u>You and one of your parents</u>* will each be asked to answer a PediQUEST Survey. Each survey takes 10-15 minutes.

You can answer all the questions at once or you can pause the survey if you need a break, or stop filling out the survey at any point. None of the answers you provide will directly become part of your medical record.

We expect you to answer each survey within 2 days of receiving it. If you don't answer, an email reminder or notification will be sent to you daily. If after 2 days you

have not answered, that survey will become unavailable and a new survey will become available the next week.

Placement into one of the study groups (randomization)

- If you or your parent have answered at least two surveys during the first two weeks of the study you will be placed into one of two groups: the PediQUEST Response (intervention) or the usual care (control) group. The group assignment is randomly made by the PediQUEST web system (you cannot choose which group you will be in and nor can we). If you have not answered at least two surveys, you will not continue to be in the study.

From randomization up to end of study (16 weeks of follow-up)

- If you are in the ***intervention group***:
  - You will continue to receive and fill out *weekly PediQUEST Surveys* and will start receiving *PediQUEST Reports (by mail or through the App)*. PediQUEST Reports summarize your answers and that of your parent's and show them in a graphical manner. If you wish to look at the report from the computer, you will need to set up an account with the *send secure system*, a system that protects your privacy (this is done only once and we can show you how to do this).

    The primary oncology team (doctor, nurse practitioner, nurse and psychosocial clinician) and the PediQUEST Response Team clinicians will also receive the report via email or App. No one other than your clinicians and the research staff will have direct access to these reports.
  - You and your parents will also meet with the *PediQUEST Response Team* as soon as this can be scheduled. The research coordinator will let the Response Team know that you have been assigned to the intervention. Somebody from that team will contact you to *schedule your first meeting with the PediQUEST*

Response Team. After this first meeting, it is recommended that you continue to meet monthly until the end of the study. Follow-up meetings, however, may be different for different patients and will depend on what you, your parents, and the Response Team arrange. During these meetings the PediQUEST Response Team will focus on understanding more about how you are doing and what, if anything, might be bothering you, so that they can make recommendations to help you feel as well as possible.

As with any other medical consultation, the PediQUEST Response Team will be in close communication with your primary oncology team and together will figure out what may be done to help you feel better. If you are already feeling better by the time you meet with the PediQUEST Response team, they may be able to provide suggestions to prevent symptoms from coming back as strongly. Sometimes, they may recommend that you see another specialist.

If one of your reports shows that you are in distress, or if the clinical team made some recommendations to ease your distress, a PediQUEST Response team member may call you to check in about how you are feeling. All interactions with the Response Team will be documented in your medical record. Also, they may communicate with your oncology team face-to-face, by phone or by email.

- If you are in the ***control group***:
  - You will continue to receive _care as usual_
  - You and your parent will continue to receive and fill out _weekly PediQUEST surveys_, but neither you nor your parents or providers will receive reports. You will _not_ have contact with the PediQUEST Response team as part of the study.
- ***Regardless of the arm*** of the study in which you are in,
  - Members of the research staff will regularly review your medical chart to collect information about your illness and treatments during the study period and,
  - Parents will complete other surveys.

End of Study (Week 18)

- At the end of the study, we will ask you to participate in an exit interview, which will be audio-recorded, so that we can learn more about how the study went.

Token of Appreciation

- As a token of appreciation for your participation in the study, we will provide you with a $20 gift card (e.g. Amazon) at the time of enrollment, covering the first two weeks of participation and, from there on, a $40 gift card for every four weeks you remain in the study.

D. HOW LONG WILL I BE IN THIS RESEARCH STUDY?

You will be in this research study for about eighteen weeks.

You may be taken off the research study for reasons such as:

- It is considered to be in your best interest
- The study procedures are found to be unsafe or ineffective
- There is any problem with following study procedures (for example, if you do not answer PediQUEST Surveys during the first two weeks in the study)
- You have a stem cell transplant while you are enrolled in the study.
- There are any problems with research funding
- Or for any other reason

If you are removed from the research study, an Investigator will explain to you why you were removed.

In addition, you can stop participating in the research study at any time (see section G).

E. WHAT ARE THE RISKS OR DISCOMFORTS OF THE RESEARCH STUDY?

There are risks to taking part in any research study. There are no anticipated physical risks from participating. You may become upset by completing the questionnaires or meeting with the PediQUEST Response Team. If this happens, you can stop answering one or more surveys and/or meeting with the Response Team. There also are a number of support services available to you to discuss your feelings. You can talk with your physician, nurse, or psychosocial clinician. You can also reach the head of the study, Dr. Joanne Wolfe (617-632-5286) who can help arrange support from your clinician in the Pediatric Psychosocial Oncology Program. If you have not been in contact with one, Dr. Wolfe can arrange a consultation with someone in that program.

Another risk is that, because there are two groups in the study, you may not receive the most effective treatment. There is no way to know upfront which of the two approaches is more effective, which is why we are doing the study. If during the research study, there is new information that may affect your health or willingness to participate, we will let you know. You may be asked to sign a new consent form that shows that you have been informed of new information relating to this research study.

A third risk is loss of confidentiality. We will use several security measures to protect confidentiality. They are explained in detail in section K.

F. WHAT ARE THE BENEFITS OF THE RESEARCH STUDY?

Taking part in this research study may or may not benefit you. We hope the information learned from this research study will provide sound evidence about how to best care for children with cancer or tumors and their families.

G. CAN I STOP BEING IN THE RESEARCH STUDY AND WHAT ARE MY RIGHTS?

You have the right to choose not to sign this form. If you decide not to sign this form, you cannot participate in this research study.

You can stop being in the research study at any time. Tell the research doctor or research coordinator if you are thinking about stopping or decide to stop. Leaving the research study will not affect your medical care. You can still receive your medical care from your hospital as usual

If you choose to not participate, or if you are not eligible to participate, or if you withdraw from this research study, this will not affect your present or future care and will not cause any penalty or loss of benefits to which you are otherwise entitled.

H. WHAT ARE THE COSTS?

Taking part in this research study may or may not lead to added costs to you or your insurance company. You or your insurance company will be charged for portions of your care during this research study that are considered usual care, including the PediQUEST Response Team visits. You may be responsible for co-payments and deductibles that are standard for your insurance coverage.

You can use your own computer, tablet or phone with internet access to complete PediQUEST Surveys and look at reports. If you are at the hospital or clinic, a member of the study team can provide you with a tablet to answer a PediQUEST survey if you prefer. Let them know. If you do not have a computer or tablet at home, the study can provide you with temporary use, while you are enrolled in the study, of a tablet and internet access to complete PediQUEST questionnaires.

If you have questions about your insurance coverage, or the items you might be required to pay for, please call financial services for information. The contact information for financial services:

- Boston Children's Hospital: (617) 355-7188
- Dana-Farber Cancer Institute: (617) 632-3455

The National Cancer Institute provides an online resource to help people participating in cancer clinical trials understand which services their insurance company is required by law to pay. This can be found at the website below.

http://www.cancer.gov or 1-800-4-CANCER (1-800-422-6237)

I. WHAT HAPPENS IF I AM INJURED OR BECOME SICK BECAUSE I TOOK PART IN THIS RESEARCH STUDY?

If you think you have been injured as a result of taking part in this research study, tell the person in charge of this research study as soon as possible. The research principal investigator's name and phone number are listed in this consent form.

The treating hospital will offer you the care needed to treat injuries directly resulting from taking part in this research. These treatments will be billed to your insurance company. You will be responsible for deductibles and co-payments. There are no plans to pay you or give you other compensation for the injury. You do not give up your legal rights by signing this form.

J. FUTURE USE OF DATA AND SPECIMENS

Your personal information and/or data collected during this study may be stored and used for future research. Any personal identifiers will be removed, before the data are shared, so that the information cannot be linked back to you.

K. WHAT ABOUT CONFIDENTIALITY?

We will take a number of measures to protect the privacy and security of all your personal information, but we cannot guarantee complete confidentiality of study data.

Information created by this study will be securely stored in your study file which will be saved in a password protected space in the web specifically assigned to the study.

Paper study documents will be stored in locked cabinets at the Dana-Farber Cancer Institute. Audio files will only be identified by a unique study code with no links to your name, and when transcribed to paper all personal references will be removed. Your name and other identifying information will not be accessible to anyone outside of the study team (more details about how and with whom we will share information can be found in section N). Some pieces of the study will become part of your medical record (for example medical notes written by the PediQUEST Response team).

The results of this research study may be published. You will not be identified in publications without your permission.

A description of this clinical trial will be available on http//:www.ClinicalTrials.gov, as required by U.S. Law. This web site will not include information that can identify you. At most, the website will include a summary of the results. You can search this website at any time.

L. WHOM DO I CONTACT IF I HAVE QUESTIONS ABOUT THE RESEARCH STUDY?

If you have questions about the study, please contact the research Investigator or study staff as listed below:

Dana-Farber Cancer Institute

- Joanne Wolfe, MD, MPH: 617-632-5286 or page at 617-632-3352 (Principal Investigator)
- Madeline Bilodeau: 617-632-3248 (Study Project Manager)

For questions about your rights as a research participant, please contact a representative of the Office for Human Research Studies at DFCI (617) 632-3029. This can include questions about your participation in the study, concerns about the study, a research related injury, or if you feel/felt under pressure to enroll in this research study or to continue to participate in this research study.

M. CERTIFICATE OF CONFIDENTIALITY (COC)

To help protect your privacy, we have been issued a Confidentiality Certificate from the National Institutes of Health (NIH). With this Certificate, the researchers on this study cannot be forced (for example, by court subpoena) to disclose information that may identify you in federal, state, or local civil, criminal, administrative, legislative or other proceedings.

Disclosure will be necessary upon request of a United States federal or state government agency sponsoring the project that will be used for audit or program evaluation purposes or to meet the requirements of the federal Food and Drug Administration (FDA).

You should understand that a Certificate of Confidentiality does not prevent you or a member of your family or even the research doctor from voluntarily releasing information about yourself or your involvement in this research. If an insurer, medical care provider, or employer learns about your participation, and obtains your consent to receive research information, then we cannot use the Certificate of Confidentiality to withhold this information. This means that you and your family must actively protect your own privacy.

The Certificate of Confidentiality cannot be used to prevent disclosure to state or local authorities when there is a duty to report concerns of abuse, neglect, self-harm or a danger to others.

N. PRIVACY OF PROTECTED HEALTH INFORMATION

Federal law requires Dana-Farber/Harvard Cancer Center (DF/HCC) and its affiliated research doctors, health care providers, and physician network to protect the privacy of information that identifies you and relates to your past, present, and future physical and mental health conditions (“protected health information”). If you enroll in this research study, your “protected health information” will be used and shared with others as explained below.

1. **What protected health information about me will be used or shared with others during this research?**

   - Contact information (names, addresses, and phone numbers)
   - Patient and parent's date of birth.
   - Medical record numbers, existing medical records, including mental health records.
   - New health information created from PediQUEST Web questionnaires and PediQUEST Response visits.

2. **Why will protected information about me be used or shared with others?**

   The main reasons include the following:

   - To conduct and oversee the research described earlier in this form;
   - To ensure the research meets legal, institutional, and accreditation requirements
   - Other reasons may include for intervention, payment, or health care operations.

3. **Who will use or share protected health information about me?**

   - Clinicians participating in the intervention and, DF/HCC and its affiliated researchers and entities participating in the research will use and share your protected health information. In addition, other DF/HCC offices that deal with research oversight, billing or quality assurance will be able to use and share your protected health information.

4. With whom outside of DF/HCC may my protected health information be shared?

While all reasonable efforts will be made to protect the confidentiality of your protected health information, it may also be shared with the following entities:

- Outside individuals or entities that have a need to access this information to perform functions relating to the conduct of this research on behalf of DF/HCC and its affiliates (for example, data storage companies, insurers, or legal advisors).
- The sponsor(s) of this study, and its subcontractors.
- Other research doctors and medical centers participating in this research
- Federal and state agencies (for example, the Department of Health and Human Services, the Food and Drug Administration, the National Institutes of Health, and/or the Office for Human Research Protections), or other domestic or foreign government bodies if required by law and/or necessary for oversight purposes. A qualified representative of the FDA and the National Cancer Institute may review your medical records.
- Hospital accrediting agencies
- A data safety monitoring board organized to oversee this research

Some who may receive your protected health information may not have to satisfy the privacy rules and requirements. They, in fact, may share your information with others without your permission.

5. For how long will protected health information about me be used or shared with others?

- Because research is an ongoing process, there is no scheduled date at which your protected health information that is being used or shared for this research will be destroyed.

6. Statement of privacy rights:

- You have the right to withdraw your permission for the researchers and participating DF/HCC entities to use or share your protected health information. We will not be able to withdraw all the information that already has been used or shared with others to carry out related activities such as oversight, or that is needed to ensure quality of the study. To withdraw your permission, you must do so in writing by contacting the researcher listed above in the section: "Whom do I contact if I have questions about the research study?"
- You have the right to request access to your protected health information that is used or shared during this research and that is related to your treatment or payment for your treatment, but you may access this information only after the study is completed. To request this information, please contact the researcher listed above in the section: "Whom do I contact if I have questions about the research study?"

DFCI Protocol Number:	TBD	Approved Date (DFCI IRB Approval):	TBD
Date Posted for Use:	TBD	Expiration Date (Invalid for use on or after):	TBD
OnCore Version Date:	TBD	Content Last Revised with OHRS Review:	TBD

O. PERMISSION FOR FUTURE CONTACT:

Sometimes, after analyzing the results of a study, additional research questions come up. This is especially frequent in studies like this, where you and other participants share with us a lot of high quality and in-depth information. Should this happen, we may want to contact you later to find out how you are doing or ask you questions related to your participation in this study. Your participation in these new research studies is voluntary, and you will not be penalized or lose any benefits if you refuse to participate or decide to stop.

Please indicate whether you want to be contacted if future research questions came up:

☐ Yes Initials_____________ Date___/___/_____

☐ No Initials_____________ Date___/___/_____

P. DOCUMENTATION OF ASSENT

Signature of participant under age 18: The person doing this research study has explained what will happen to me if I take part in this research study. My signature below means that I want to be in this research study and agree to be audio-recorded if I participate in an exit interview. I can decide not to take part in this research study if I don't want to and nothing at all will happen if I decide I do not want to participate.

_____________________________________ ___________________

Signature of Participant Date

To be completed by person obtaining assent:

The assent discussion was initiated on ________________ (date).

☐ The information was presented in age-appropriate terms. The minor:

- ☐ Agreed to take part in the study
- ☐ Did not agree to take part in the study

☐ An assent discussion was not initiated with the minor for the following reason(s):

- ☐ Minor is incapacitated
- ☐ Minor is under 10 years of age
- ☐ Other____
  ____

Signature of Individual obtaining assent: ____

Printed name of above: ____

Date: _____________

Q. DOCUMENTATION OF CONSENT

Signature of participant over the age of 18:

My signature below indicates:

- I have had enough time to read the consent and think about participating in this study;
- I have had all of my questions answered to my satisfaction;
- I am willing to participate in this study;
- I have been told that my participation is voluntary and I can withdraw at any time
- I agree to be audio-recorded if participating in an exit interview.

_____________________________________ _______________________________

Signature of Participant (if over 18 years old) Date

R. DOCUMENTATION OF PERMISSION

Signature of parent/legally authorized representative of participants under the age of 18.

My signature below indicates:

- I have had enough time to read the consent and think about my child/teen participation in this study;
- I have had all my questions answered to my satisfaction;
- I permit my child/teen's participation in this study;
- I have been told that participation is voluntary and s/he can withdraw at any time
- I agree that my child/teen is audio-recorded if participating in an exit interview.

____________________________________ ______________________________

Signature of Legally Authorized Representative Date

____

Relationship of Legally Authorized Representative to Participant

(To be completed by person obtaining consent)

Adult Participants

The consent discussion was initiated on ___________________ (date).

Signature of individual obtaining consent: ____

Printed name of above: ____

Date: _______________

☐ A copy of this signed consent form will be given to the participant or legally authorized representative, or, where the participant is a minor, the participant's parent or legal guardian.

For Adult Participants

☐ 1) The participant is an adult and provided consent to participate.

☐ 1a) Participant (or legally authorized representative) is a non-English speaker and signed the translated Short Form in lieu of English consent document:

As someone who understands both English and the language spoken by the participant, I interpreted and/or witnessed, in the participant's language, the researcher's presentation of the English consent form. The participant was given the opportunity to ask questions.

Signature of Interpreter/Witness: ____

Printed Name of Interpreter/Witness: ____

Date: _______________

☐ 1b) Participant is physically unable to sign the consent form because:

☐ The participant is illiterate.

☐ The participant has a physical disability.

☐ Other (please describe): ________________________________

The consent form was read to the participant who was given the opportunity to ask questions and who communicated agreement to participate in the research.

Signature of Witness: ____

Printed Name of Witness: ____

Date: _______________

☐ 2) The participant is an adult who lacks capacity to provide consent and his/her legally authorized representative:

☐ 2a) gave permission for the adult participant to participate

☐ 2b) did not give permission for the adult participant to participate

Minor Participants

To be completed by person obtaining consent:

The consent discussion was initiated on ___________________ (date).

Signature of individual obtaining consent: ____

Printed name of above: ____

Date: _______________

☐ A copy of this signed consent form will be given to the participant or legally authorized representative, or, where the participant is a minor, the participant's parent or legal guardian.

☐ 1) The parent or legally authorized representative gave permission for the minor to participate.

☐ 1a) Parent or legally authorized representative is a non-English speaker and signed the translated Short Form in lieu of English consent document

As someone who understands both English and the language spoken by the participant, I interpreted and/or witnessed, in the participant's language, the researcher's presentation of the English consent form. The participant was given the opportunity to ask questions.

Signature of Interpreter/Witness: ____

Printed name of Interpreter/Witness: ____

Date: _______________

☐ 1b) Parent or legally authorized representative is physically unable to sign the consent form because:

☐ The participant is illiterate.

☐ The participant has a physical disability.

☐ Other (please describe): ____

The consent form was presented to the parent or legally authorized representative who was given the opportunity to ask questions and who communicated agreement for the minor to participate in the research.

Signature of Witness: ____

Printed Name of Witness: ____

Date: _______________

☐ The parent or legally authorized representative did not give permission for the minor to participate

DFCI Protocol Number: TBD	Approved Date (DFCI IRB Approval): TBD
Date Posted for Use: TBD	Expiration Date (Invalid for use on or after): TBD
OnCore Version Date: TBD	Content Last Revised with OHRS Review: TBD

Dear <name>,

You are receiving this letter because you and your parent are eligible for our research study, PediQUEST Response, and we would like to invite you to be part of it. We know from previous studies that children, teens, and young adults with cancer or tumors experience physical and emotional symptoms that can bother them, even though there are available treatments for these symptoms that may help. PediQUEST Response is a new way of providing care that aims to improve quality of life for young patients with cancer or tumors and their parents. We want to learn whether children, teens, and young adults receiving PediQUEST Response do in fact feel better than those receiving usual care.

Below you will find additional information about the study. If you do not wish to participate in the study or be further contacted about it, please complete the opt-out document attached and email it back to pediquestweb@dfci.harvard.edu. If we do not receive the opt-out email, a member of our study team will contact you via phone to provide additional details about the study and see if you and your parent are willing to participate. Your child's oncologist is aware that we are inviting you to be part of this study.
The decision to participate is yours!

Who is supporting this research?
The National Institute of Nursing Research (NINR) are supporting this research study by providing funding.

What is involved in this research study?
If you and your parent agree to participate, you will fill out online PediQUEST surveys (on a computer, tablet or phone) every week for a total of 18 weeks. The graphic below summarizes study activities, and additional information can be found below.

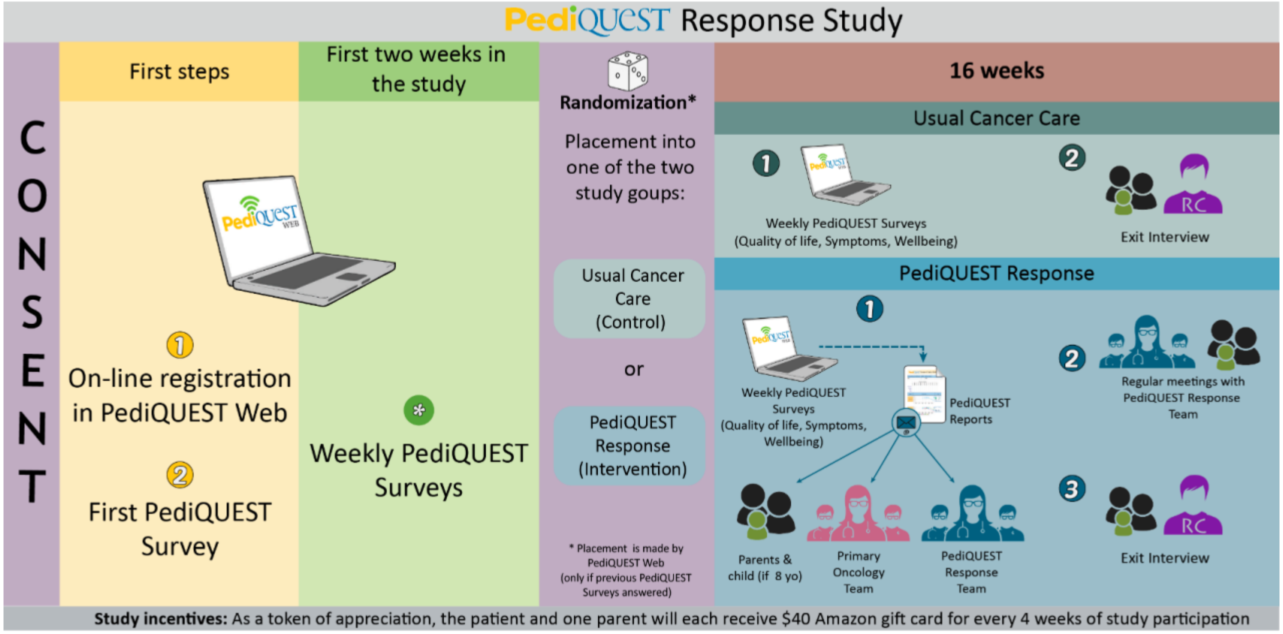

Registration: If you decide to participate, a research coordinator will work with you to complete online registration using PediQUEST web, the system where you will complete weekly surveys.

Completing weekly surveys: Each week, you and your parent will complete weekly surveys through either the PediQUEST website or mobile app.

- These surveys take about 10-15 minutes to complete. They ask about how you have been feeling and any symptoms you may be having. It also asks about other aspects of life such as school or friends.

Parent Surveys: Your parent will also receive a baseline survey and five additional monthly surveys throughout the entire study that ask about s/he has been feeling and how s/he feels about certain aspects of your care. These surveys take between 15 and 25 minutes to complete.

Placement into one of the study groups (randomization): After two weeks of completing weekly surveys, you and your parent will be placed into one of two groups, the PediQUEST Response (intervention) or the usual care (control) group. The group assignment is randomly made by the PediQUEST web system (you cannot choose which group you and your parent will be in and nor can we). If you have not answered at least two surveys, you will not continue to be in the study.

- *Intervention group:* If you are in the intervention group, you will continue to receive and fill out weekly PediQUEST Surveys and will start receiving PediQUEST reports via e-mail or the App. Your primary oncology team will also receive these reports. Reports summarize your and your parent's answers using graphs. You will also meet the Response Team, at the hospital or virtually. Response is an interdisciplinary team of doctors, nurse practitioners, and social workers who specialize in symptom management and strategies to optimize quality of life. They will also receive PediQUEST reports and work with you, your parent, and your oncology team to help you all feel better when needed.
- *Usual care group:* If you are in the usual care group, you and your parent will answer PediQUEST surveys and you will continue to receive care as usual, but neither you nor your parent, or your providers will receive PediQUEST reports. You will not have contact with the Response Team as part of the study.
- *For all participants:* Regardless of which group you are in, members of the research staff will regularly review your medical chart to collect information about your illness and treatments during the study period.

End of study: At the end of the study, we will invite you to participate in an audio-recorded exit interview so that we can learn more about your experiences in the study.

Token of appreciation: As a token of appreciation, you and your parent will each receive a $40 Amazon gift card for each month you remain in the study.

What are the benefits, risks, or discomforts of the research study?

Taking part in this research study may or may not benefit you and/or your parent. We hope the information learned from this research study will help us to understand the experience of children, adolescents, and young adults with cancer or tumors and their families. It is possible

that you may become upset answering the questionnaires or meeting with the Response Team. If this happens, you can skip any questions that you do not wish to answer or skip surveys, and/or stop meeting with the Response Team. You can also stop being on the study at any point. Support services are available to you if it would be helpful to discuss any feelings that arise due to your participation in this study. You can also talk with your physician, nurse or psychosocial clinician. Another risk is that, because there are two groups in the study, you may not receive the most effective treatment. There is no way to know upfront which of the two approaches is more effective and this why we are doing the study. If during the research study there is new information that may affect your health or your willingness to participate, we will let you know. A third risk is loss of confidentiality. We will use several security measures to protect confidentiality, as described below.

Confidentiality and Future Use of Data
We will take measures to protect the privacy and security of all your and your parent's personal information, but we cannot guarantee complete confidentiality of study data. Paper study documents will be stored in locked cabinets at the Dana-Farber Cancer Institute. Handling of electronic information will be protected by strong security measures (including the use of passwords and data encryption) and will be stored on secure servers. In the case that we record an interview, audio files will only be identified by a unique study code and no direct links to your or your parent's name will be used; when audio files are transcribed, all personal references will be removed. Study data including your names and other identifying information will not be accessible to anyone outside of the study team. Your personal information and/or data collected during this study may be stored and used for future research. Any personal identifiers will be removed, before they are shared, so that the information cannot be linked back to you.

Alternatives
Participation in this study is completely voluntary. If you choose to participate, you may stop participating at any time. Your decision to participate or not participate will not impact the quality or type of medical care you or your family receives at Dana-Farber or Boston Children's Hospital. If you decide to participate in this study, we may want to contact you again for future research.

This project has been approved by the institutional Review Board of Dana-Farber Cancer Institute. If you have further questions, you may contact Madeline Bilodeau, Project Manager, at 617-632-3248, or Joanne Wolfe, Principle Investigator, at 617-632-5286. If you have any questions regarding your rights as a research participant or any research-related questions or concerns, please contact the Office for Human Research Studies at Dana-Farber at 617-632-3029.

Thank you for considering participation in this study. We believe it will provide us with important insight into the experiences of children, adolescents, and young adults so that we can better care for patients like you in the future.

Research Consent Form

for Social and Behavioral Research

Dana-Farber/ Harvard Cancer Center

BIDMC/BCH/BWH /DFCI/MGH/Partners Network Affiliates

OHRS 09.23.16a

**Protocol Title:** The PediQUEST Response Intervention Study
**DF/HCC Principal Research Investigator / Institution:** Joanne Wolfe, DFCI
**DF/HCC Site-Responsible Research Investigator(s) / Institution(s):** Christina Ullrich, DFCI/BCH
**CHILD WRITTEN PERMISSION/ASSENT FORM**

If you are a parent or guardian of a child under 18 years old, the word "you" refers to your child. You, the parent, will be asked to read and sign this document to give permission for your child to participate.

INTRODUCTION AND KEY INFORMATION

We do research to learn how to take better care of patients like you. You can always choose if you want to take part in a research study or not. We would like you to think if you want to participate in this study or not. If you want to participate in this study, please sign at the end of this form. We will also ask your parents to sign and will give you a copy so you can look at it at any time.

We will start by giving you a short explanation about the study so you can quickly decide if you are interested. If you are interested, you can continue reading this document to learn more about the study.

1. **Why am I being invited to take part in a research study?**

We are asking you to be in a research study called "The PediQUEST Response Intervention Study." You are invited to participate because you are a child that is getting treatment for a type of cancer or tumor that may cause symptoms and discomfort.

2. **Why is this research being done?**

DFCI Protocol Number:	TBD	Approved Date (DFCI IRB Approval):	TBD
Date Posted for Use:	TBD	Expiration Date (Invalid for use on or after):	TBD
OnCore Version Date:	TBD	Content Last Revised with OHRS Review:	TBD

We are doing this study because we know that children can be bothered by pain, nausea, sadness, and other feelings. To try and make these feelings better, we created . PediQUEST Response which uses a software, and a team of doctors and nurses to improve quality of life for patients like you and their parents. We do this study to learn whether children, teens, and young adults that use PediQUEST Response do in fact feel better than those receiving usual care.

3. Who is supporting this research?

The National Institute of Nursing Research (NINR) from the National Institutes of Health is providing the funding for this study.

4. What does this research study involve and how long will it last?

You and one of your parents will answer some questions about how you are feeling. You may also meet a team of doctors and nurses, and when the study is over we may ask you some questions about how it was to be part of the study.

In the PediQUEST Response Study it is expected that about 400 people (200 children and 200 parents) from five different hospitals will take part.
Your doctor is aware that we are inviting you and your parent to participate in this study. However, you will decide whether to participate. If you choose not to participate, are not eligible to participate, or withdraw from this research study, this will not affect your present or future care at the institution.

In the next sections of this form we will explain more about why this research study is being done, what is PediQUEST Response, what it means to participate in this study, possible risks and benefits of participation, alternatives to participation, and your rights as a research participant. A “participant” is a person who participates in a research study.

We encourage you to take some time to think this over and to talk with other people and to ask questions now and at any time in the future.

A. WHY IS THIS RESEARCH STUDY BEING DONE?

As we mentioned above, we are doing this study because we know that children can be bothered by pain, nausea, sadness, and other feelings. To try and make these feelings better, we created a computer program called PediQUEST (which stands for Pediatric Quality of Life and Evaluation of Symptoms Technology). We use PediQUEST to ask children (and parents) some questions about how they have been feeling.

In the PediQUEST Response Study, we plan to:

1) Use PediQUEST to ask how you have been feeling every week. What you tell us will be emailed to you, your parents and primary team (doctors, nurses, and psychosocial clinician). You can also use the PediQUEST App to answer the questions and see the answers.

2) Connect families with the *PediQUEST Response Team*, a team made up of doctors, nurse practitioners, and social workers who specialize in treating pain and other feelings that can bother you. The PediQUEST Response team will also receive PediQUEST emails and will work with your primary team to help you feel better.

In this study we will compare PediQUEST Response to the care that is usually provided where you receive your treatment.

B. WHAT OTHER OPTIONS ARE THERE?

Taking part in this research study is for you to decide. Instead of being in this research study, you can:

- Decide not to participate in this research study and instead receive usual care

C. WHAT IS INVOLVED IN THE RESEARCH STUDY?

If you and your parent decide to participate, both of you will be asked to fill out online PediQUEST surveys every week for a total of 18 weeks (see below for more on these Surveys). We will ask one of your parents to answer some other questionnaires as well. In the next few pages we will explain in detail what will happen during the study, starting with a **figure in the next page that summarizes study activities.**

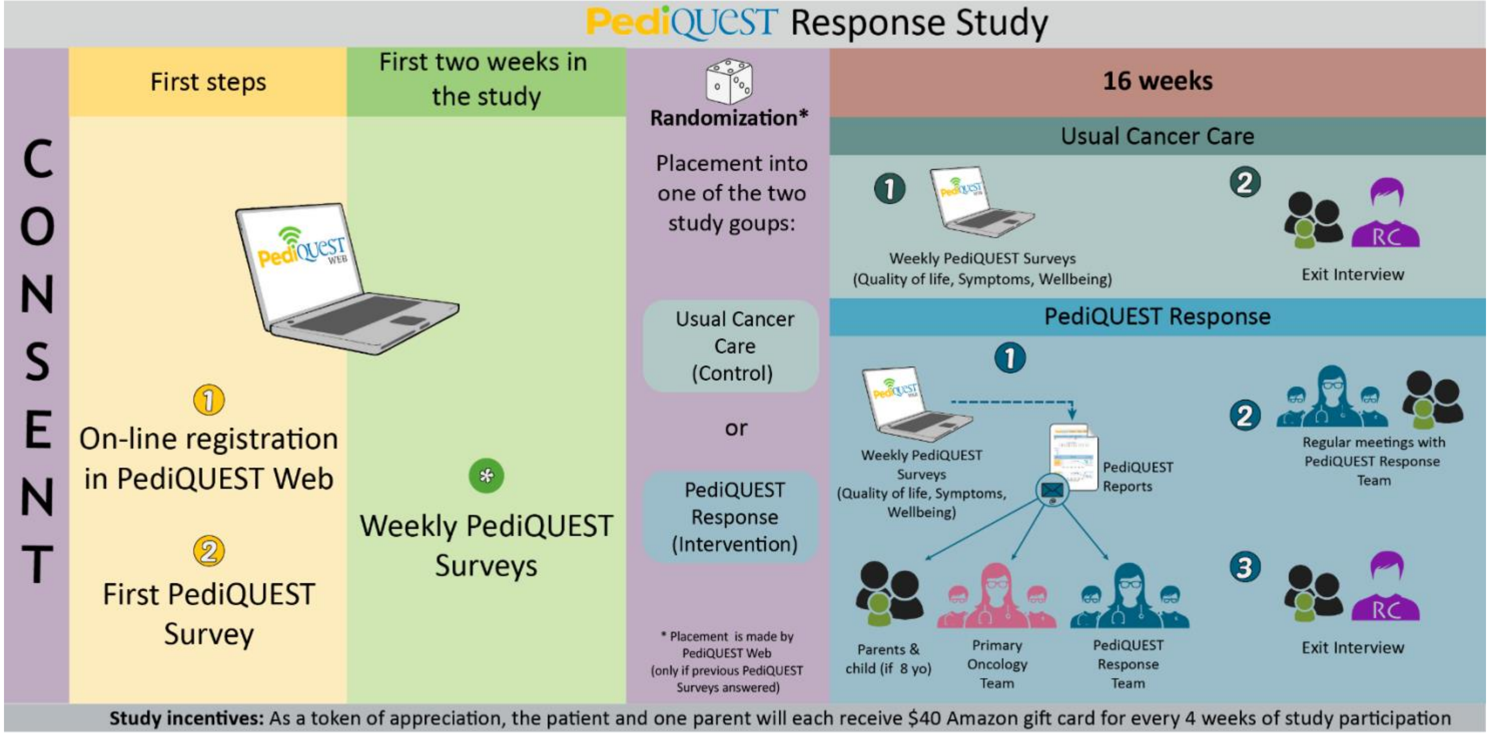
PediQUEST Response Study
CONSENT
First steps
1 On-line registration in PediQUEST Web
2 First PediQUEST Survey
First two weeks in the study
Weekly PediQUEST Surveys
Randomization*
Placement into one of the two study goups:
Usual Cancer Care (Control)
or
PediQUEST Response (Intervention)
* Placement is made by PediQUEST Web (only if previous PediQUEST Surveys answered)
16 weeks
Usual Cancer Care
1 Weekly PediQUEST Surveys (Quality of life, Symptoms, Wellbeing)
2 Exit Interview
PediQUEST Response
1 Weekly PediQUEST Surveys (Quality of life, Symptoms, Wellbeing)
PediQUEST Reports
Parents & child (if 8 yo)
Primary Oncology Team
PediQUEST Response Team
2 Regular meetings with PediQUEST Response Team
3 Exit Interview
Study incentives: As a token of appreciation, the patient and one parent will each receive $40 Amazon gift card for every 4 weeks of study participation

Right after signing the consent:

- The research coordinator will work with you to complete ***on-line registration in PediQUEST web***: You will be registered as a PediQUEST user. An email is required. Your parent will have a separate account. We can help to set up a new email account if needed. You can access the PediQUEST system through a computer or a mobile App (for cell phones and tablets). A research coordinator can help you to set-up the app on your device if needed.
- You and your parent will be assigned the first ***PediQUEST survey***. The research coordinator will walk you through the survey and help you become familiar with it. More details are explained below.

First two weeks in the study:

- During the first two weeks, we ask that you answer ***weekly PediQUEST Surveys***.

PediQUEST Surveys ask how you have been feeling, for example if pain, nausea, or other symptoms are bothering you. It also asks about how you are feeling about other things in life, such as school and friends. PediQUEST Surveys can be answered on any electronic device from anywhere that you can connect to the internet. Whenever a new survey is available for you to answer, PediQUEST web will send an email with the link to the survey or a notification if you are using the App.

Who will answer a PediQUEST Survey?

- *You and one of your parents* will each be asked to answer a PediQUEST Survey. Each survey takes 10-15 minutes.

You can answer the questions all at once or you can pause the survey at any point if you need a break, or want to stop filling it out.

We expect you to answer each survey within 2 days of receiving it. If you don't answer, an email reminder or notification will be sent to you daily. If after 2 days you have not answered, that survey will become unavailable and a new survey will become available the next week.

Placement into one of the study groups (randomization)

- If you or your parent have answered at least two surveys during the first two weeks of the study you will be placed into one of two groups: the PediQUEST Response (intervention) or the usual care (control) group. The group assignment is made by the PediQUEST web system (you cannot choose which group you will be in and nor can we). If you have not answered at least two surveys, you will not continue to be in the study.

From group placement up to end of study (16 weeks)

- If you are in the ***intervention group***:
  - You will continue to receive and fill out *weekly PediQUEST Surveys* and will start receiving *PediQUEST Reports (by email or through the App)*. PediQUEST Reports sum up your and your parent's answers using graphs. If you wish to look at the report from the computer, you will need to set up an account with the send secure system, this system protects your privacy (this is done only once and we will show you how to do this).

    The primary oncology team (doctor, nurse practitioner, nurse and psychosocial clinician) and the PediQUEST Response Team clinicians will also receive the report via email or App. No one other than your clinicians and the research staff will have direct access to these reports.
  - You and your parents will also meet with the *PediQUEST Response Team* as soon as this can be scheduled. After the first meeting it is recommended that you continue to meet monthly until the end of the study although this will depend on what you, your parents, and the Response Team arrange. During these meetings, the PediQUEST Response Team will focus on understanding more about how you are doing and what, if anything, might be bothering you, so that they can make recommendations to help you feel as well as possible. The

Response Team will be in close communication with your primary oncology team and together will figure out what may be done to help you feel better. They may be able to provide recommendations to prevent symptoms from coming back as strongly, or they may recommend that you see another specialist. In some cases, PediQUEST Response team members may call to check in about how you are feeling or how did the recommended treatments make you feel.

- If you are in the ***control group***:
  - You will continue to receive *care as usual*
  - You and your parent will continue to receive and fill out *weekly PediQUEST surveys*, but neither you nor your parents or providers will receive reports. You will *not* have contact with the PediQUEST Response team as part of this study.
- ***Regardless of the arm*** of the study in which you are in,
  - During the study period, research staff will regularly review your medical chart to collect information about your illness and treatments and,
  - Parents will complete other surveys.

End of Study (Week 18)

- At the end of the study, we will also ask you to participate in an *exit interview*, which will be audio-recorded, so that we can learn more about how the study went.

Token of Appreciation

- As a token of appreciation for your participation in the study, we will provide you with a $20 gift card (e.g. Amazon) when you enroll, and after the first two weeks, a $40 gift card for every four weeks you remain in the study.

D. HOW LONG WILL I BE IN THIS RESEARCH STUDY?

You will be in this research study for about eighteen weeks.

You may be taken off the research study for reasons such as:

- If we think it is best for you
- The study procedures are not safe or don't work
- There is any problem with following study procedures (for example, if you do not answer PediQUEST Surveys during the first two weeks in the study)
- You have a stem cell transplant while you are on the study
- There are any problems with research funding
- Or for any other reason

If you are removed from the research study, an Investigator will explain to you why you were removed.

In addition, you can stop participating in the research study at any time (see section G).

E. WHAT ARE THE RISKS OR DISCOMFORTS OF THE RESEARCH STUDY?

You may become upset by completing the questionnaires or meeting the PediQUEST Response Team. If this happens, you can stop answering one or more surveys and/or meeting with the Response Team. You will also be able to discuss your feelings with your doctor, nurse, or psychosocial clinician. You can also reach the head of the study, Dr. Joanne Wolfe (617-632-5286) who can help arrange support from your clinician in the Pediatric Psychosocial Oncology Program. If you have not been in contact with one, Dr. Wolfe can arrange a consultation with someone in that program.

If during the research study, there is new information that may affect your health or willingness to participate, we will let you know. You may be asked to sign a new consent form that shows that you have been informed of new information relating to this research study.

A third risk is loss of confidentiality, meaning your information doesn't remain private. We will use security measures to protect confidentiality. They are explained in detail in section K.

F. WHAT ARE THE BENEFITS OF THE RESEARCH STUDY?

Taking part in this research study may or may not benefit you. We hope the information learned from this research study will provide good information about how to best care for children with cancer or tumors and their families.

G. CAN I STOP BEING IN THE RESEARCH STUDY AND WHAT ARE MY RIGHTS?

You have the right to choose not to sign this form. If you decide not to sign this form, you cannot participate in this research study.

You can stop being in the research study at any time. Tell the research doctor or research coordinator if you are thinking about stopping or decide to stop. Leaving the research study will not affect your medical care. You can still receive your medical care from your program.

If you choose to not participate, or if you are not eligible to participate, or if you stop participating in this research study, this will not affect your present or future care and will not cause any problems in receiving care.

H. WHAT ARE THE COSTS?

Taking part in this research study may or may not lead to added costs to you or your insurance company.

You can use your own computer, tablet or phone with internet to complete PediQUEST Surveys and look at reports. If you are at the hospital or clinic, a member of the study

team can give you a tablet to answer a PediQUEST survey if you prefer. Let them know. If you do not have a computer or tablet at home, the study can provide you, while you are in the study, with a tablet and internet access to complete PediQUEST questionnaires.

I. WHAT HAPPENS IF I AM INJURED OR BECOME SICK BECAUSE I TOOK PART IN THIS RESEARCH STUDY?

If you think you have been injured as a result of taking part in this research study, tell the person in charge of this research study as soon as possible. The research principal investigator's name and phone number are listed in this consent form.

J. FUTURE USE OF DATA AND SPECIMENS

Your personal information and/or data collected during this study may be stored and used for future research. Any personal identifiers will be removed, before the data are shared, so that the information cannot be linked back to you.

K. WHAT ABOUT CONFIDENTIALITY?

To protect the privacy and security of all your personal information, we will do several things, however, we cannot guarantee complete confidentiality of study data.

A description of this clinical trial will be available on http//:www.ClinicalTrials.gov, as required by U.S. Law. This web site will not include information that can identify you. At most, the website will include a summary of the results. You can search this website at any time.

L. WHOM DO I CONTACT IF I HAVE QUESTIONS ABOUT THE RESEARCH STUDY?

If you have questions about the study, please contact the research Investigator or study staff as listed below:

Dana-Farber Cancer Institute

- Joanne Wolfe, MD, MPH: 617-632-5286 or page at 617-632-3352 (Principal Investigator)
- Madeline Bilodeau: 617-632-3248 (Study Project Manager)

For questions about your rights as a research participant, please contact a representative of the Office for Human Research Studies at DFCI (617) 632-3029. This can include questions about your participation in the study, concerns about the study, a research related injury, or if you feel/felt under pressure to enroll in this research study or to continue to participate in this research study.

M. CERTIFICATE OF CONFIDENTIALITY (COC)

To help protect your privacy, we have been issued a Confidentiality Certificate from the National Institutes of Health (NIH). With this Certificate, the researchers on this study cannot be forced (for example, by court subpoena) to disclose information that may identify you in federal, state, or local civil, criminal, administrative, legislative or other proceedings.

Disclosure will be necessary upon request of a United States federal or state government agency sponsoring the project that will be used for audit or program evaluation purposes or to meet the requirements of the federal Food and Drug Administration (FDA).

You should understand that a Certificate of Confidentiality does not prevent you or a member of your family or even the research doctor from voluntarily releasing information about yourself or your involvement in this research. If an insurer, medical care provider, or employer learns about your participation, and obtains your consent to receive research information, then we cannot use the Certificate of Confidentiality to withhold this information. This means that you and your family must actively protect your own privacy.

The Certificate of Confidentiality cannot be used to prevent disclosure to state or local authorities when there is a duty to report concerns of abuse, neglect, self-harm or a danger to others.

N. PRIVACY OF PROTECTED HEALTH INFORMATION

Federal law requires Dana-Farber/Harvard Cancer Center (DF/HCC) and its affiliated research doctors, health care providers, and physician network to protect the privacy of information that identifies you and relates to your past, present, and future physical and mental health conditions ("protected health information"). If you enroll in this research study, your "protected health information" will be used and shared with others as explained below.

1. What protected health information about me will be used or shared with others during this research?

- Contact information (names, addresses, and phone numbers)
- Patient and parent's date of birth.
- Medical record numbers, existing medical records, including mental health records.
- New health information created from PediQUEST Web questionnaires and PediQUEST Response visits.

2. Why will protected information about me be used or shared with others?

The main reasons include the following:

- To do the research described earlier in this form;
- To be sure the research meets legal, institutional, and accreditation requirements
- Other reasons may include for intervention, payment, or health care operations.

3. Who will use or share protected health information about me?

- Clinicians participating in the interventions and, DF/HCC and its affiliated researchers and entities participating in the research will use and share your protected health information. In addition, other DF/HCC offices that deal with research oversight, billing or quality assurance will be able to use and share your protected health information.

4. With whom outside of DF/HCC may my protected health information be shared?

While all reasonable efforts will be made to protect the confidentiality of your protected health information, it may also be shared with the following entities:

- Outside individuals or entities that have a need to access this information to perform functions relating to the conduct of this research on behalf of DF/HCC and its affiliates (for example, data storage companies, insurers, or legal advisors).
- The sponsor(s) of this study, and its subcontractors.
- Other research doctors and medical centers participating in this research.
- Federal and state agencies (for example, the Department of Health and Human Services, the Food and Drug Administration, the National Institutes of Health, and/or the Office for Human Research Protections), or other domestic or foreign government bodies if required by law and/or necessary for oversight purposes. A qualified representative of the FDA and the National Cancer Institute may review your medical records.
- Hospital accrediting agencies
- A data safety monitoring board organized to oversee this research

Some who may receive your protected health information may not have to satisfy the privacy rules and requirements. They, in fact, may share your information with others without your permission.

5. For how long will protected health information about me be used or shared with others?

- Because research is an ongoing process, there is no scheduled date at which your protected health information that is being used or shared for this research will be destroyed.

6. Statement of privacy rights:

- You have the right to withdraw your permission for the researchers and participating DF/HCC entities to use or share your protected health information. We will not be able to withdraw all the information that already has been used or shared with others to carry out related activities such as oversight, or that is needed to ensure quality of the study. To withdraw your permission, you must do so in writing by contacting the researcher listed above in the section: "Whom do I contact if I have questions about the research study?"
- You have the right to request access to your protected health information that is used or shared during this research and that is related to your treatment or payment for your treatment, but you may access this information only after the study is completed. To request this information, please contact the researcher listed above in the section: "Whom do I contact if I have questions about the research study?"

O. ASSENT FOR FUTURE CONTACT:

Sometimes, after analyzing the results of a study, additional research questions come up. This is especially frequent in studies like this, where you and other participants share with us a lot of information. Should this happen, we may want to contact you later to find out how you are doing or ask you questions related to your participation in this study.

Your participation in these new research studies is your choice, and you will not be penalized or lose any benefits if you refuse to participate or decide to stop.
Please indicate whether you want to be contacted if future research questions came up:

☐ Yes Initials_____________ Date___/___/_____

☐ No Initials_____________ Date___/___/_____

P. DOCUMENTATION OF ASSENT

Signature of participant under age 18: The person doing this research study has explained what will happen to me if I take part in this research study. My signature below means that I want to be in this research study and agree to be audio-recorded if I participate in an exit interview. I can decide not to take part in this research study if I don't want to and nothing at all will happen if I decide I do not want to participate.

______________________________________ ____________________

Signature of Participant Date

To be completed by person obtaining assent:

The assent discussion was initiated on _______________ (date).

☐ The information was presented in age-appropriate terms. The minor:

- ☐ Agreed to take part in the study
- ☐ Did not agree to take part in the study

☐ An assent discussion was not initiated with the minor for the following reason(s):

- ☐ Minor is incapacitated
- ☐ Minor is under 10 years of age
- ☐ Other____

  ____

Signature of Individual obtaining assent: _____________________________________

Printed name of above: ____

Date: _____________

Q. DOCUMENTATION OF PERMISSION

Signature of parent:

My signature below indicates:

- I have had enough time to read the consent and think about my child/teen participation in this study;
- I have had all my questions answered to my satisfaction;
- I permit my child/teen's participation in this study;
- I have been told that participation is voluntary and s/he can withdraw at any time
- I agree that my child/teen is audio-recorded if participating in an exit interview.

_____________________________________ _____________________________

Signature of Legally Authorized Representative Date

____

Relationship of Legally Authorized Representative to Participant

Adult Participants

To be completed by person obtaining consent:

The consent discussion was initiated on ___________________ (date).

Signature of individual obtaining consent: ____

Printed name of above: ____

Date: _______________

☐ A copy of this signed consent form will be given to the participant or legally authorized representative, or, where the participant is a minor, the participant's parent or legal guardian.

For Adult Participants

☐ 1) The participant is an adult and provided consent to participate.

☐ 1a) Participant (or legally authorized representative) is a non-English speaker and signed the translated Short Form in lieu of English consent document:

As someone who understands both English and the language spoken by the participant, I interpreted and/or witnessed, in the participant's language, the researcher's presentation of the English consent form. The participant was given the opportunity to ask questions.

Signature of Interpreter/Witness: ____

Printed Name of Interpreter/Witness: ____

Date: _______________

☐ 1b) Participant is physically unable to sign the consent form because:

☐ The participant is illiterate.

☐ The participant has a physical disability.

☐ Other (please describe): ___________________________________

The consent form was read to the participant who was given the opportunity to ask questions and who communicated agreement to participate in the research.

Signature of Witness: ____

Printed Name of Witness: ____

Date: _______________

☐ 2) The participant is an adult who lacks capacity to provide consent and his/her legally authorized representative:

☐ 2a) gave permission for the adult participant to participate

☐ 2b) did not give permission for the adult participant to participate

Minor Participants

To be completed by person obtaining consent:

The consent discussion was initiated on ____________________ (date).

Signature of individual obtaining consent: ____

Printed name of above: ____

Date: _______________

☐ A copy of this signed consent form will be given to the participant or legally authorized representative, or, where the participant is a minor, the participant's parent or legal guardian.

☐ 1) The parent or legally authorized representative gave permission for the minor to participate.

☐ 1a) Parent or legally authorized representative is a non-English speaker and signed the translated Short Form in lieu of English consent document

As someone who understands both English and the language spoken by the participant, I interpreted and/or witnessed, in the participant's language, the researcher's presentation of the English consent form. The participant was given the opportunity to ask questions.

Signature of Interpreter/Witness: ____

Printed name of Interpreter/Witness: ____

Date: _______________

☐ 1b) Parent or legally authorized representative is physically unable to sign the consent form because:

☐ The participant is illiterate.

☐ The participant has a physical disability.

☐ Other (please describe): ____

The consent form was presented to the parent or legally authorized representative who was given the opportunity to ask questions and who communicated agreement for the minor to participate in the research.

Signature of Witness: ____

Printed Name of Witness: ____

Date: _______________

☐ The parent or legally authorized representative did not give permission for the minor to participate

DFCI Protocol Number: TBD	Approved Date (DFCI IRB Approval): TBD
Date Posted for Use: TBD	Expiration Date (Invalid for use on or after): TBD
OnCore Version Date: TBD	Content Last Revised with OHRS Review: TBD

Helping Other Kids and Teens Like You...

By taking part, you'll help us learn about how *to better care for kids and teens like you.*

We really appreciate you telling us about your symptoms and quality of life. Remember, you can always choose not to take part or stop taking part in the study at any time. Your clinical team will still treat you the same way they always have.

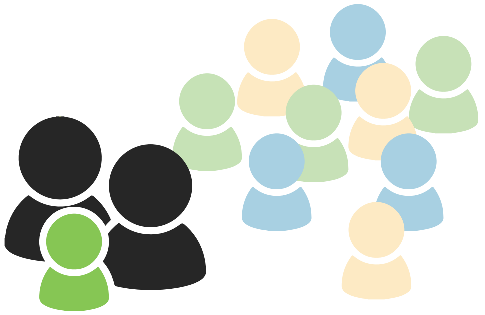

PediQUEST Response

Do You Have Any Questions?

You or your parents can contact us:

Joanne Wolfe, MD, MPH
Principal Investigator
Dana-Farber Cancer Institute
Boston Children's Hospital
(617) 632-5286
Joanne_Wolfe@dfci.harvard.edu

Madeline Bilodeau
Research Project Manager
Dana-Farber Cancer Institute
Boston Children's Hospital
(617) 632-3248
Madeline_Bilodeau@dfci.harvard.edu

Christina Ullrich, MD, MPH
Site Principal Investigator
Dana-Farber Cancer Institute
Boston Children's Hospital
(617) 632-4997
Christina_Ullrich@dfci.harvard.edu

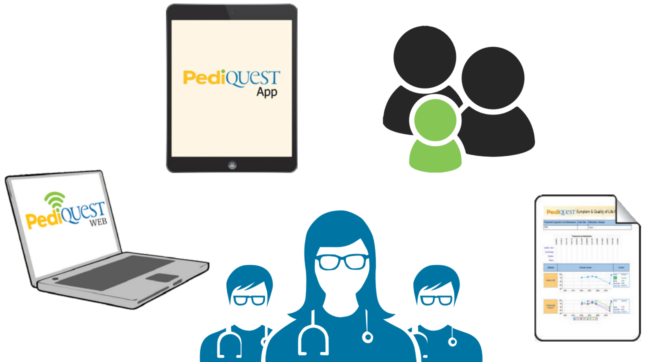

The PediQUEST Response Intervention Study

A study about improving quality of life...

Child / Teen / Young Adult Brochure

DANA-FARBER Boston Children's
CANCER AND BLOOD DISORDERS CENTER

PediQUEST

We invite you and your parent to take part in "The PediQUEST Response Intervention Study".

You are eligible for this study because you are receiving treatment for cancer or a tumor. In dealing with your illness, there may be times when you don't feel so well because of things such as pain, nausea, or sadness. We are exploring a new way of providing care to try to help patients like you feel better. We called this new care model the *PediQUEST Response Intervention* and it consists of:

1

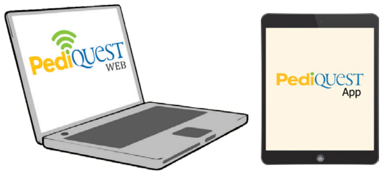

A website (PediQUEST Web) and a mobile application (PediQUEST App), where you answer questions about how you are feeling.

2

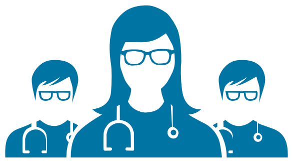

Regular meetings with the Response Team, a team made up of doctors, nurses, and social workers who are experts in managing symptoms and helping to improve quality of life.

Through this research study we hope to learn whether children and teens receiving the PediQUEST Response Intervention feel better than those who receive usual care.

If you decide to take part in this study...

... we will ask you to answer an online questionnaire once a week for about 4 and a half months. The PediQUEST Survey (PQ-Survey) will ask how you have been feeling. This usually takes about 10-15 minutes to finish and can be answered on a smart phone, computer or tablet. At some point during the study, you will be placed into one of two groups: The PediQUEST Response group or the usual care group.

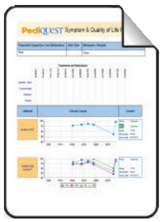

If you are in the PediQUEST Response group, you, your parents, and your providers will receive a report after every PQ survey. We will explain how to read these reports. You will also meet with the Response Team providers who will work with you, your family, and your oncology team to improve your quality of life.

If you are in the usual care group, we will ask you to answer PQ-Surveys and you will receive the same care as you have been receiving.

At the end of the study, we may want to talk with you and your parents about your experiences with the study.

As a token of our appreciation for your participation, you will receive $40 gift cards (e.g. Amazon) for each month of study participation

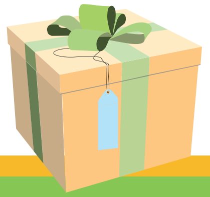

Supplement: S2 Protocol — (PDF) [file pone.0277212.s003.pdf]
