# Supplementary material for: A multisite randomized controlled trial of an early palliative care intervention in children with advanced cancer: The PediQUEST Response Study Protocol
Source: PLoS One. 2022 Nov 8;17(11):e0277212. doi: 10.1371/journal.pone.0277212 (PMC9642881; doi:10.1371/journal.pone.0277212)
Supplement: S3 Protocol — (PDF) [file pone.0277212.s004.pdf]

Dana-Farber Cancer Institute

Office for Human Research Studies

Notification of IRB Outcome - Approval

Date: April 21, 2022
To: Joanne Wolfe, MD
From: The Office for Human Research Studies (OHRS)

On 04/21/2022 the IRB reviewed the following protocol:

**IRB Protocol Number:**	17-102
**Type of Review:**	Submission Correction for Amendment
**Title:**	A multisite, parallel, randomized controlled trial to compare the effectiveness of an early palliative care intervention, the Pediatric Quality of Life and Evaluation of Symptoms Technology Response to Pediatric Oncology Symptom Experience (PediQUEST Response), versus Usual Cancer Care in children and adolescents with advanced cancer.
**Principal Investigator:**	Wolfe, Joanne, MD
**iRIS Reference Number:**	432321
**Review Process:**	Expedite
**Sponsor(s):**	DF/HCC Investigator, Dana-Farber/Harvard Cancer Center, National Institute of Nursing Research/NIH/DHHS
**Submission Description:**	We are submitting this amendment to add our statistical team at Deakin University as a formal site on the protocol.
**Participating Sites:**	Boston Children's Hospital (BCH) Dana-Farber Cancer Institute (DFCI)
**Documents Revised:**	Submission Components Approved (see below)

Submission Components Approved

Document Type	Version
Submission-Amendment	Version 17.1
Document-Protocol Clean 4.14	Version 1.0

The submitted protocol has been approved in its entirety.

Current state of additional determinations (made previously or on this submission):

**Risk Level:**	Not Greater than Minimal Risk under 45 CFR 46 / 21 CFR 56
**Expedited Review Category:**	N/A<br>Category 7: Research on individual or group characteristics or behavior (including, but not limited to, research on perception, cognition, motivation, identity, language, communication, cultural beliefs or practices, and social behavior) or research employing survey, interview, oral history, focus group, program evaluation, human factors evaluation, or quality assurance methodologies

**Documentation of Consent:**	Written consent in accordance with 45 CFR 46.117/ 21 CFR 50.27<br>Partial waiver approved under 45 CFR 46.117 (c) 1 or 2/ 21 CFR 56.109 (c)1
**HIPAA:**	HIPAA Authorization for research approved under 45 CFR 164.508 (a) (1)<br>Partial waiver of HIPAA Authorization for Research approved under 45 CFR164.512 (i) (2) (ii)
**Child Risk Assessment:**	45 CFR 46.404/ 21 CFR 50.51): Not greater than minimal risk
**Parental Permission:**	One parent signature

The IRB approved the protocol from 04/21/2022 to 11/07/2022 inclusive. Within 60 days prior to the expiration date you must submit a continuing review or study completion and any required attachments. If continuing review approval or study completion is not granted before the expiration date, your protocol will be put on hold.

As Principal Investigator you are responsible for the following:

1. Submission in writing of any and all changes to this protocol (e.g., protocol, recruitment materials, consent form, study completion) to the IRB for review and approval prior to initiation of the change(s), except where necessary to eliminate apparent immediate hazards to the subject(s). Changes made to eliminate apparent immediate hazards to subjects must be reported to the IRB within 24 hours.
2. Submission in writing of any and all serious adverse event(s) that occur during the course of this protocol in accordance with the IRB's policy on adverse event reporting.
3. Submission in writing of any and all unanticipated problems involving risks to subjects or others.
4. Use of only IRB approved copies of the protocol, consent form(s), questionnaire(s), letter(s), and advertisement(s) in your research. Do not use expired consent forms.
5. Informing all investigators listed on the protocol of changes, adverse events, and unanticipated problems.

If you have any questions, please contact OHRS at OHRS@dfci.harvard.edu.

cc:
Christina K Ullrich, MD, MPH, Hasan Al-Sayegh, Veronica Dussel, MD, MPH, Alexandra Merz, Deborah Feifer, Erika Tsuchiyose, Opeyemi Awofeso, Madeline Bilodeau

Reviewed/Revised Submission Components

Study Document	
Title	Version Date
Protocol Clean 4.14	04/14/2022
