# Supplementary material for: A multisite randomized controlled trial of an early palliative care intervention in children with advanced cancer: The PediQUEST Response Study Protocol
Source: PLoS One. 2022 Nov 8;17(11):e0277212. doi: 10.1371/journal.pone.0277212 (PMC9642881; doi:10.1371/journal.pone.0277212)
Supplement: S4 Protocol — (PDF) [file pone.0277212.s005.pdf]

DANA-FARBER
CANCER INSTITUTE

Office for Human Research Studies
Dana-Farber Cancer Institute
Dana-Farber/Harvard Cancer Center
450 Brookline Ave. (OS-229)
Boston, MA 02215
Tel: (617) 632-3029
Fax: (617) 632-2686

New Application: Notification of IRB Approval

DFCI Protocol No.: 17-102

Date: 05/31/2017

To: Joanne Wolfe, MD

From: OHRS

Title of Protocol:	A multisite, parallel, randomized controlled trial to compare the effectiveness of an early palliative care intervention, the Pediatric Quality of Life and Evaluation of Symptoms Technology Response to Pediatric Oncology Symptom Experience (PediQUEST Response), versus Usual Cancer Care in children and adolescents with advanced cancer.
Submit Date:	03/02/2017
Regulatory Sponsor:	DF/HCC Investigator
Funding Sponsor:	Dana-Farber/Harvard Cancer Center; National Institute of Nursing Research/NIH/DHHS (Pending)
Protocol Version Date:	02/21/2017
Review Type:	Full
Note:	HIPAA authorization waived.<br>The grant was reviewed and determined to be consistent with the protocol.
Cond. Approval Date:	05/08/2017
Approval Date:	05/30/2017
Expiration Date:	05/08/2018

This protocol has been reviewed and approved by the Dana-Farber Cancer Institute (DFCI) IRB.

There may be further administrative/activation sign-off conditions that must be met prior to enrolling participants on this protocol. The administrative and/or activation requirements will be communicated to the study team as appropriate. A separate notification will be sent when the protocol has been activated and posted to OncPro.

As Principal Investigator you are responsible for the following:

1. Submission in writing of any and all changes to this protocol (e.g., protocol, recruitment materials, consent form, study completion) to the IRB for review and approval prior to initiation of the change(s), except where necessary to eliminate apparent immediate hazards to the subject(s). Changes made to eliminate apparent immediate hazards to subjects must be reported to the IRB within 24 hours.
2. Submission in writing of any and all serious adverse event(s) that occur during the course of this protocol in accordance with the IRB's policy on adverse event reporting.
3. Submission in writing of any and all unanticipated problems involving risks to subjects or others.
4. Use of only IRB approved copies of the protocol, consent form(s), questionnaire(s), letter(s), and advertisement(s) in your research. Do not use expired consent forms.

5. Informing all investigators listed on the protocol of changes, adverse events, and unanticipated problems.

If you have any questions, please contact OHRS at (617) 632-3029.

cc:

Veronica Dussel, MD, MPH, Dana-Farber Cancer Institute

Hasan Al-Sayegh, Dana-Farber Cancer Institute

Madeline Bilodeau, Dana-Farber Cancer Institute

Christina Ullrich, MD, MPH, Dana-Farber Cancer Institute

Anne Reed-Weston, Dana-Farber Cancer Institute

CTO DFCI Clinical Trials Office, Dana-Farber Cancer Institute
