# Supplementary material for: A multisite randomized controlled trial of an early palliative care intervention in children with advanced cancer: The PediQUEST Response Study Protocol
Source: PLoS One. 2022 Nov 8;17(11):e0277212. doi: 10.1371/journal.pone.0277212 (PMC9642881; doi:10.1371/journal.pone.0277212)
Supplement: Attachment — Submitted filename: Plos One Review October 2022.docx [file pone.0277212.s006.docx]

**Manuscript Number:** PONE-D-22-16245
**Manuscript Title:** A multisite randomized controlled trial of an early palliative care intervention in children with advanced cancer: the PediQUEST Response Study Protocol

This study protocol for the PediQUEST Response Study is exemplary in its description of the study protocol. There is a clear, comprehensive, and sophisticated data analytic plan. A good rationale is provided for how to handle multiple informant information. Likewise, a reasonable discussion of contamination risk and the plan to address it.

I have only a few minor suggestions to make.

Page 8 line 161 Please name the 5 study sites.

Page 20 line 450 Mention Certificate of Confidentiality from NIH as another protection against breaches to confidentiality.

Page 23, line 526, please update manuscript in preparation if applicable.
